# Palliative Video Consultation and Symptom Distress Among Rural Inpatients: A Randomized Clinical Trial

**DOI:** 10.1001/jamanetworkopen.2025.19426

**Published:** 2025-07-09

**Authors:** Marie A. Bakitas, Shena Gazaway, Felicia Underwood, Christiana Ekelem, Vantrice T. Heard, Richard Kennedy, Andres Azuero, Rodney Tucker, Susan McCammon, Joshua M. Hauser, Lucas McElwain, Ronit Elk

**Affiliations:** 1 Acute, Chronic and Continuing Care, School of Nursing, University of Alabama at Birmingham; 2 Division of Geriatrics, Gerontology, and Palliative Medicine, Department of Medicine, Heersink School of Medicine, University of Alabama at Birmingham; 3 Center for Palliative and Supportive Care, University of Alabama at Birmingham; 4 Family, Community, and Health Systems, School of Nursing, University of Alabama at Birmingham; 5 Center for Patient Flow, University of Alabama at Birmingham Health System; 6 Center for Palliative and Supportive Care, Heersink School of Medicine, Division of Geriatrics, Gerontology, and Palliative Medicine, University of Alabama at Birmingham; 7 Northwestern Medicine Feinberg School of Medicine, Northwestern University, Chicago, Illinois; 8 Geriatrics, Memory Care, and Home Palliative Care, North Mississippi Regional Medical Center, Tupelo

## Abstract

**Importance:**

Palliative care has been shown to improve important patient outcomes but is rarely available in small or rural hospitals.

**Objective:**

To assess whether culturally based palliative care video consultation could improve symptom distress compared with usual inpatient care without palliative care.

**Design, Setting, and Participants:**

Recruitment for this multisite, single-blind randomized clinical trial occurred from July 20, 2020, to December 20, 2023; data collection was completed on January 15, 2024. A community-aided approach was used to recruit inpatients from 3 rural hospitals lacking palliative care services in Alabama, Mississippi, and South Carolina. Self-identified non-Hispanic Black or African American and non-Hispanic White adults aged 55 years or older admitted with a serious chronic illness and a willing caregiver were invited to participate. Randomization was stratified by site and race.

**Interventions:**

Participants were randomized 1:1 to Community Tele-Pal, culturally based video consultation by a palliative care specialist followed by research coordinator (RC) contacts 3 and 6 days after video consultation (intervention arm), or to routine hospital care (control arm).

**Main Outcomes and Measures:**

The primary outcome was the between-group difference in the change in patient-reported symptom distress from baseline to day 7, assessed by the Edmonton Symptom Assessment Scale (ESAS; range, 0-90; lower scores indicate lower symptom distress). Secondary outcomes were quality of life (QOL; Patient-Reported Outcomes Measurement Information System global physical and mental health mean *T* scores), resource use (emergency department visits and hospital readmissions), and an exploratory outcome of feeling heard and understood.

**Results:**

A total of 209 patients were randomized (104 to usual care; 105 to the intervention); mean (SD) age was 73.3 (8.3) years, 120 (57.4%) were female, 58 (27.8%) were Black or African American, 157 (75.1%) were retired, and 75 (35.9%) had a Palliative Performance Scale score less than 70% (indicating need for functional assistance). On day 7, the mean (SE) ESAS score change from baseline was −11.4 (1.5) points in the intervention group and −7.3 (1.5) points in the control group; the between-group difference in change in ESAS scores was not statistically significant (Westfall *d*, −0.28; 95% CI, −0.56 to 0.01; *P* = .055). The mean (SE) day 7 between-group difference in ESAS score of −4.0 (1.8) points met the criteria for a minimal clinically important difference of 3 to 4 points. No between-group differences were observed for QOL, resource use, or feeling heard and understood.

**Conclusions and Relevance:**

In this randomized clinical trial, the intervention was not associated with reduced symptom distress at day 7 vs baseline or with improved QOL or reduced resource use compared with usual care. However, the between-group difference in the ESAS score met the criteria for a minimal clinically important difference of 3 to 4 points. Palliative video consultation to reduce health care inequities for hospitalized rural-dwelling individuals may warrant further investigation.

**Trial Registration:**

ClinicalTrials.gov Identifier: NCT03767517

## Key Points

**Question:**

Is community-developed, culturally based palliative care video consultation in rural hospitals without palliative care services superior to usual care in improving symptom distress in Black or African American and White inpatients admitted with serious illnesses?

**Findings:**

In this randomized clinical trial of 209 inpatients, mean symptom distress score change at day 7 was −11.4 points for video consultation vs −7.3 points for usual care. The between-group difference in scores was not statistically significant but met the criteria for a minimal clinically important difference (3-4 points).

**Meaning:**

Palliative video consultation to reduce health care inequities for hospitalized rural-dwelling individuals may warrant further investigation.

## Teaser

This randomized clinical trial evaluates whether culturally based palliative care video consultation in rural hospitals without palliative care services improves symptom distress among inpatients with serious illnesses compared with usual care.

## Introduction

The triple threat of rural geography, racial inequities, and older age has hindered access to high-quality palliative care for many people in the US.^1,2,3,4,5,6,7,8^ The rural southeastern US has few facilities offering palliative care. Only 70% of the deep South vs 85% to 94% of the rest of the US has palliative care^7^ despite the deep South having the greatest needs due to suboptimal health care access and elevated morbidity and mortality.^6,8^

Telehealth has been effective in providing specialty palliative care to rural and frontier areas,^2,9,10,11,12^ homes,^13^ and clinics.^13,14,15^ A prior study by our team, ENABLE (Educate, Nurture, Before Life Ends), pioneered delivery of early palliative care via telehealth to rural outpatients with cancer^14,15^ and heart failure^16^ and family caregivers.^17,18,19^ However, unlike rural hospital emergency medicine and intensive care,^20,21^ inpatient palliative care via telehealth is understudied. A retrospective study,^22^ case series,^23,24^ and reviews^25,26,27^ reported positive experiences with inpatient palliative care via telehealth, especially for the pandemic-induced surge of critically ill inpatients needing goals-of-care discussions. However, expanding inpatient palliative care via telehealth to areas with unique cultures, such as rural hospitals in the southeastern US, requires more than a technological connection.

Cultural considerations, highlighted by the National Consensus Project (NCP) guidelines, are essential to providing high-quality palliative care.^28^ Since initial palliative care studies were conducted in urban academic centers serving largely White, middle-class, educated populations,^29,30,31^ culturally diverse individuals with unique health and illness beliefs and historical mistrust of the medical system have been less receptive to palliative care.^1,32,33,34^ For example, despite some recent improvement,^35^ Black or African American persons historically have had the lowest hospice and palliative care use.^3,36,37^ Use of community-engaged methods, such as community-based participatory research (CBPR), has resulted in culturally responsive care strategies that may reduce health disparities in underrepresented populations.^1,38,39,40,41,42^

While dimensions of rurality,^2,9^ culture,^29,32^ and telehealth^9,11,13^ have been individually addressed to improve palliative care access and acceptability, we aimed to synergize these efforts by implementing a community-developed video consultation strategy for rural hospitals without palliative care,^43^ especially for groups that traditionally have underused palliative care.^1,3,28,34,43^ First, using a CBPR approach, a prior study by our team partnered with southern, rural Black and White community members and conducted focus groups for caregivers of hospitalized seriously ill patients to culturally and linguistically elevate their voices about practices that respected their cultural values and beliefs.^28,43^ Each group’s beliefs and communication preferences were incorporated into the NCP-recommended standard palliative care consultation.^29^ Second, community experts were leveraged to provide our team with a cultural immersion experience to provide a deep understanding of and insights on cultural values and communication preferences.^43^ The community experts proposed simple acts like addressing patients respectfully as “Mr” or “Mrs,” establishing rapport by conversing about the local community, avoiding medical jargon, and respecting how cultural and religious lenses impacted patients’ health and illness beliefs. Third, community advisory boards (CABs) were established at each site to gain community-informed insights about acceptable study procedures. Fourth, CAB members were engaged as recruitment ambassadors^38^ and were provided a stipend for each recruitment visit.

The purpose of this multisite randomized clinical trial (RCT) was to evaluate whether community-developed, culturally based palliative care video consultation could improve symptom distress, quality of life (QOL), and resource use ^44^ compared with usual care in hospitals without palliative care; an exploratory outcome of feeling heard and understood was also assessed. We hypothesized that culturally based video consultation would be superior to usual hospital care in reducing symptom distress and improving resource use and QOL.

## Methods

### Design

In accordance with the Consolidated Standards of Reporting Trials (CONSORT) reporting guideline for RCTs,^45^ Community Tele-Pal was a 3-site, single-blind RCT with 1:1 allocation to the video consultation intervention^43,46^ or usual care among seriously ill older adults. The University of Alabama at Birmingham, Russell Medical Center, Aiken Medical Center, and Anderson Medical Center institutional review boards approved the study protocol^46^ and analytic plan (Supplement 1). The trial was registered at ClinicalTrials.gov (NCT03767517) prior to startup. Patients and caregivers individually provided written informed consent.

### Participants

Eligible patients self-identified as non-Hispanic Black or African American or non-Hispanic White because the original intervention development study included only Black and White non-Hispanic individuals. The intervention was only designed for cultural considerations of non-Hispanic Black or White patients, and during screening, those self-identifying as another race or as Hispanic ethnicity were not eligible and were not approached to participate. Eligible patients were also aged 55 years or older, had cancer or predefined noncancer serious chronic illness^47^ (eAppendix 1 in Supplement 2), had a Callahan cognitive screen score of 3 or higher,^48^ had a participating caregiver (defined as someone who knew the patient well and was involved in their care),^18,19^ and completed baseline measures. Patient exclusion criteria were receiving hospice care; not home dwelling; self-identified race other than Black or African American or White or ethnicity other than non-Hispanic; currently using recreational drugs; and having schizophrenia, bipolar disorder, or alcohol misuse. Eligible caregivers were willing to participate and complete baseline measures. Patients and caregivers received $40 ($10 at baseline, $10 at day 7, and $20 at day 30) for completing outcome measures.

### Settings and Recruitment

Recruitment occurred between July 20, 2020, and December 20, 2023; data collection was completed on January 15, 2024. The 3 rural-serving hospitals (in Alabama, South Carolina, and Mississippi) lacked palliative care and had sufficient non–intensive care unit admissions to meet recruitment goals (eTable 1 in Supplement 2). Site teams comprised a local hospitalist, blinded and unblinded research coordinators, an 8-member CAB (comprising health care professionals, community leaders, patients and family members, and faith leaders), and remote culturally trained board-certified palliative care clinicians.

Local research coordinators screened medical admissions daily and, with hospitalist agreement, used a community-informed recruitment approach wherein a racially concordant (when available) CAB member introduced themself and the study goals to eligible patients.^38^ (Until May 31, 2021, due to COVID-19 visitor restrictions, CAB members’ prerecorded videos were substituted for in-person approaches.^38^) After CAB introduction, research coordinators reviewed the completed informed consent and baseline questionnaires from patients and caregivers agreeable to the study.

### Randomization and Blinding

The study statistician (R.K.) developed a computer-generated block randomization schema stratified by study site and self-identified (Black or African American, White) race. Following consent and completion of baseline questionnaires, the research coordinator notified the University of Alabama at Birmingham–based program manager (F.U.), who provided the local unblinded research coordinator with the patient’s group assignment. The unblinded research coordinator notified the patient and, within 48 hours, organized a mutually convenient time for the video consultation with the remote palliative care physician.

### Conditions

#### Community Tele-Pal Intervention

The video consultation intervention protocol, which recommends culturally tailored approaches based on participants’ race (eTable 2 in Supplement 2),^43,46^ was conducted by a remote palliative care specialist with the patient, caregiver (if available), and unblinded research coordinator using a secure telehealth platform and equipment. Within 24 hours, the remote palliative care clinician entered their consultation note and recommendations on a standardized template (eAppendix 2 in Supplement 2) into the patients’ electronic medical record, which was immediately available to the attending hospitalist. The unblinded research coordinator then followed up in person or by telephone 3 and 6 days following the video consultation with the patient and caregiver to address recommendations or notify the hospitalist about recommendations that needed attention. Research coordinators also documented whether postconsultation recommendations were being followed in the secure REDCap database.^49^

#### Video Consultation Fidelity Monitoring

Trained research coordinators and study staff (F.U., C.E.) completed a fidelity checklist (eAppendix 3 in Supplement 2) indicating whether the audiorecorded video consultation visit followed protocol. Fidelity monitoring was completed on each clinician’s first 3 video consultations and a 10% random sample thereafter. Protocol fidelity was excellent (mean [SD] of 89% [0.32%] adherence).

#### Usual Care

Usual care included all standard hospital procedures, medical care, and referrals (eg, social work) that would be present in a hospital that lacked palliative care services. Usual care was determined at the discretion of the attending hospitalist.

### Data Collection and Outcome Measures

An unblinded local research coordinator collected self-reported baseline sociodemographic characteristics and outcome measures prior to randomization. Immediately following the video consultation, patients completed a 10-item telehealth satisfaction questionnaire with a 6-point Likert scale (1, “strongly agree”; 6, “strongly disagree”). Thereafter, a blinded research coordinator collected day 7 and day 30 outcome measures by telephone.

#### Primary Outcome

The primary outcome was the between-group difference in the change in patient-reported symptom distress from baseline to day 7, measured by the 9-item Edmonton Symptom Assessment Scale (ESAS) (score range, 0-90; lower scores indicate less symptom distress).^50^ A change of 3 to 4 points in ESAS score from baseline was considered a minimal clinically important difference.^51^

#### Secondary Outcomes

Secondary outcomes were patient and caregiver QOL, assessed via the 10-item Patient-Reported Outcomes Measurement Information System (PROMIS) Global Health-10 instrument, version 1.2, which measures physical and mental health.^52^ Summed raw PROMIS scores are converted to *T* scores, where 50 is the mean and 10 is the SD (higher scores indicate better health).^52^ Resource use was measured as self-reported readmissions or emergency visits from discharge until day 30.

Additional caregiver outcomes were caregiver burden, measured by the 14-item Montgomery Borgatta Caregiver Burden Scale with 3 subscales: objective burden (score range, 6-30), demand burden (score range, 4-20), and stress burden (score range, 4-20); higher scores indicate higher burden.^53^ Satisfaction was measured by the 20-item Family Satisfaction With End-of-Life Care (FAMCARE) Scale on a 5-point Likert scale (1, “very satisfied”; 5, “very dissatisfied”), with a total score range of 20 to 100 (higher scores indicate greater satisfaction).^54^

#### Exploratory Outcome

Feeling heard and understood was a single-item measure assessed by the question, “During your hospitalization, how much have you felt/did you feel heard and understood by the doctors, nurses, and hospital staff?” Responses were on a 5-point Likert scale (“completely,” “quite a bit,” “moderately,” “slightly,” or “not at all”).^44^

### Statistical Analysis

#### Sample Size

We targeted an enrollment of 352 patients (176 per treatment group), resulting in 80% power at α = .05 to detect a standardized effect of 0.3 for the primary outcome and Westfall *d* (Cohen *d* with Westfall adjustment,^55^ used for mixed-effects models) of 0.33 at α = .025 for the secondary and exploratory outcomes (Westfall *d* of 0.20 was considered small; 0.50, medium; and 0.80, large).^56^ COVID-19 slowed recruitment rates, so in July 2022, using prior assumptions, the projected recruitment of 250 patients (125 per treatment group) resulted in 80% power at α = .05 to detect a Cohen *d* of 0.36 for the primary outcome and of 0.40 at α = .025 for the secondary and exploratory outcomes.

#### Data Analysis

Consistent with an intent-to-treat analysis, all participants were included regardless of participation. We used descriptive statistics (*t* tests for continuous variables and χ^2^ tests for categorical variables). For study outcomes, we used mixed models^57^ with fixed effects of group, time, time × group interaction, and participant random effects to model longitudinal changes. For continuous outcomes, we used a linear mixed model with a normal link function.^57^ For categorical outcomes, we used a multinomial logistic link function.^58^ We used a covariance pattern structure^57^ to model the correlation among repeated measures, with time (day) treated as a categorical measure. We estimated intervention effects by calculating between-group contrasts of the estimated marginal means for the score (for continuous outcomes) or the proportion of patients (for categorical outcomes) at day 7 and day 30, subtracting the baseline value from each. All *P* values were from 2-sided tests. Results for the primary outcome were deemed statistically significant at *P* = .05. We also performed a sensitivity analysis using an alternate model to estimate the between-group differences across the follow-up time points, including the baseline value as a covariate for the adjustment as a fixed effect. Data were analyzed using R, version 4.42 (R Project for Statistical Computing).

## Results

### Participants and Sample Characteristics

**Figure 1.**
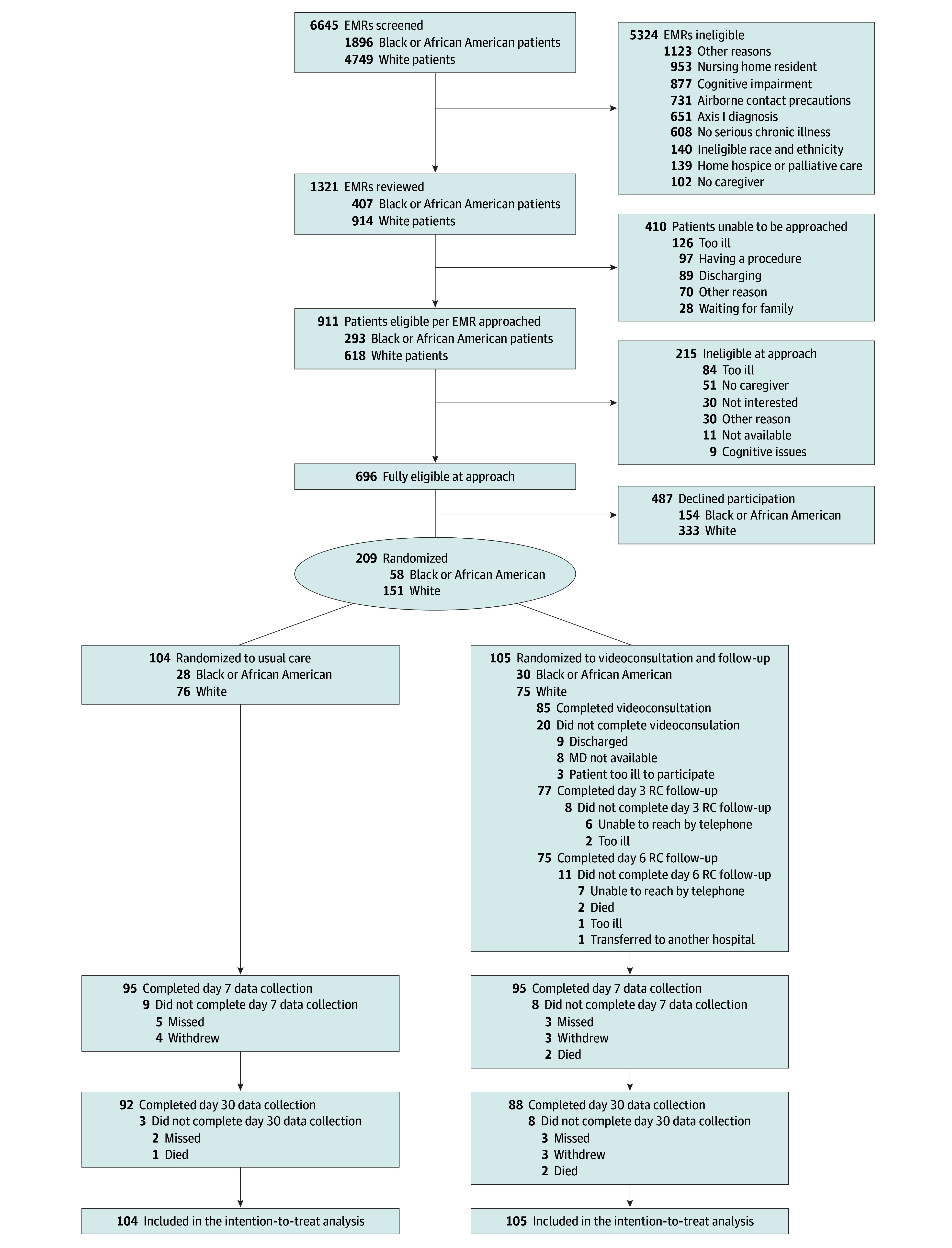
Participant Flow EMR indicates electronic medical record; RC, research coordinator.

Between July 20, 2020, and December 20, 2023, of the 696 patients approached, 487 declined and 209 were randomized (30.0% acceptance rate): 105 to the intervention and 104 to usual care (Figure 1). There were no differences in baseline characteristics between those who consented and those who declined (eTable 3 in Supplement 2). Participant characteristics and patient-reported outcomes were similar between treatment groups (Table 1). Patient participants’ mean (SD) age was 73.3 (8.3) years; 120 (57.4%) were female, and 89 (42.6%) were male. A total of 58 patients (27.8%) self-identified as Black or African American and 151 (72.2%) as White; 157 (75.1%) were retired. Seventy-five (35.9%) had a Palliative Performance Scale (PPS) score less than 70%, indicating reduced function requiring assistance,^59^ and the mean (SD) Charleson Comorbidity Index score was 4.6 (2.7), indicating moderate to high severity of illness.^60,61^ Enrolled caregivers (N = 209) had a mean (SD) age of 60.1 (15.1) years; 155 (74.2%) were female, 144 (68.9%) were married, 119 (56.9%) had some college education, and 135 (64.6%) lived with the patient. Baseline caregiver measures were balanced between groups (eTable 4 in Supplement 2).

**Table 1.** Patient Baseline Characteristics

Characteristic	Participants^a^		
	All (N = 209)	Intervention (n = 105)	Usual care (n = 104)
Age, mean (SD), y	73.3 (8.3)	72.9 (8.7)	73.7 (7.7)
Gender			
Female	120 (57.4)	53 (50.5)	67 (64.4)
Male	89 (42.6)	52 (49.5)	37 (35.6)
Race^b^			
Black or African American	58 (27.8)	30 (28.6)	28 (26.9)
White	151 (72.2)	75 (71.4)	76 (73.1)
Marital status			
Married	104 (49.8)	60 (57.1)	44 (42.3)
Widowed	55 (26.3)	24 (22.9)	31 (29.8)
Divorced	30 (14.4)	11 (10.5)	19 (18.3)
Never married	10 (4.8)	6 (5.7)	4 (3.8)
Living with a partner	5 (2.4)	3 (2.9)	2 (1.9)
Separated	5 (2.4)	1 (1.0)	4 (3.8)
Educational level			
≤Eighth grade	8 (3.8)	2 (1.9)	6 (5.8)
Some high school	46 (22.0)	29 (27.6)	17 (16.3)
High school graduate or GED	62 (29.7)	30 (28.6)	32 (30.8)
Some college or technical school	57 (27.3)	25 (23.8)	32 (30.8)
College graduate	28 (13.4)	14 (13.3)	14 (13.5)
Graduate degree	8 (3.8)	5 (4.8)	3 (2.9)
Employment			
Retired	157 (75.1)	74 (70.5)	83 (79.8)
Unemployed (disability)	33 (15.8)	18 (17.1)	15 (14.4)
Full time	9 (4.3)	7 (6.7)	2 (1.9)
Part time	7 (3.3)	5 (4.8)	2 (1.9)
Homemaker	2 (1.0)	1 (1.0)	1 (1.0)
Other	1 (0.5)	0	1 (1.0)
Religious preference			
Protestant	174 (83.3)	87.0 (82.9)	87.0 (83.7)
Catholic	10 (4.8)	6.0 (5.7)	4.0 (3.8)
Jewish	1 (0.5)	1.0 (1.0)	0
Other	13 (6.2)	7.0 (6.7)	6.0 (5.8)
None	11 (5.3)	4.0 (3.8)	7.0 (6.7)
Insurance status			
Commercial, alone or with additional insurance plan	25 (12.0)	17.0 (16.2)	8.0 (7.7)
Medicare, alone or with additional insurance plan	129 (61.7)	61.0 (58.1)	68.0 (65.4)
Medicaid, alone or with additional insurance plan	36 (17.2)	18.0 (17.1)	18.0 (17.3)
Military, alone or with additional insurance plan	19 (9.1)	9.0 (8.6)	10.0 (9.6)
Medical condition			
Cardiac disease	169 (80.9)	81 (77.1)	88 (84.6)
Kidney disease	100 (47.8)	50 (47.6)	50 (48.1)
Pulmonary disease	81 (38.8)	35 (33.3)	46 (44.2)
Cancer	60 (28.7)	32 (30.5)	28 (26.9)
Stroke	29 (13.9)	13 (12.4)	16 (15.4)
Sepsis	19 (9.1)	7 (6.7)	12 (11.5)
Hepatic disease	11 (5.3)	7 (6.7)	4 (3.8)
Neurodegenerative disease	11 (5.3)	5 (4.8)	6 (5.8)
Other	26 (12.4)	12 (11.4)	14 (13.5)
Charlson Comorbidity Index score, mean (SD)	4.6 (2.7)	4.6 (2.7)	4.5 (2.7)
Palliative Performance Scale score, %			
100	6 (2.9)	3 (2.9)	3 (2.9)
90	17 (8.1)	10 (9.5)	7 (6.7)
80	42 (20.1)	24 (22.9)	18 (17.3)
70	69 (33.0)	37 (35.2)	32 (30.8)
60	36 (17.2)	16 (15.2)	20 (19.2)
50	33 (15.8)	10 (9.5)	23 (22.1)
40	4 (1.9)	3 (2.9)	1 (1.0)
30	1 (0.5)	1 (1.0)	0
20	1 (0.5)	1 (1.0)	0
Length of stay			
Overall			
Total, median (IQR), d	4.0 (2.0-6.0)	4.0 (2.0-6.0)	4.0 (2.0-6.0)
Missing, No.	3	1	2
Before consultation			
Total, median (IQR), d	NA	2.0 (2.0-3.0)	NA
Missing, No.	NA	20	NA
After consultation			
Total, median (IQR), d	NA	1.0 (0.0-3.0)	NA
Missing, No.	NA	21	NA
Patient-reported outcomes			
Primary: ESAS Total Symptom Distress score, mean (SD)^c^	36.0 (16.1)	36.2 (16.8)	35.9 (15.4)
Missing, No.	4	2	2
Secondary: PROMIS Global Health-10 score^d^			
Physical health *T* score, mean (SD)	34.9 (8.3)	35.1 (8.0)	34.6 (8.5)
Mental health *T* score, mean (SD)	44.6 (8.0)	45.5 (7.7)	43.7 (8.2)
Missing, No.	1	1	0
Exploratory: feeling heard and understood^e^			
Completely	110 (52.6)	54 (51.4)	56 (53.8)
Quite a bit	50 (23.9)	26 (24.8)	24 (23.1)
Moderately	31 (14.8)	15 (14.3)	16 (15.4)
Slightly	10 (4.8)	7 (6.7)	3 (2.9)
Not at all	7 (3.3)	2 (1.9)	5 (4.8)
NA	1 (0.5)	1 (1)	0

Abbreviations: ESAS, Edmonton Symptom Assessment Scale; GED, General Educational Development; NA, not applicable; PROMIS, Patient-Reported Outcomes Measurement System.

^a^ Data are presented as number (percentage) of participants unless otherwise indicated.

^b^ Patients self-identified race using the categories listed. Patients were only eligible if they self-identified as not Hispanic or Latino; thus, this category totals to 100%.

^c^ Score ranges from 0 to 90; lower scores indicate less symptom distress.

^d^ Scores range from 0 to 100, with a mean (SD) score of 50 (10); higher scores indicate better health.

^e^ Single-item measure: “During your hospitalization, how much have you felt/did you feel heard and understood by the doctors, nurses, and hospital staff?”

Of the 105 participants randomized to the intervention, 85 (81.0%) completed the video consultation (Figure 1); there were no demographic differences between those who did and did not receive the video consultation (eTable 5 in Supplement 2). Patient satisfaction with the video consultation technology was 92.7% (eTable 6 and eFigure 1 in Supplement 2).

### Study End Points

#### Primary Outcome

**Figure 2.**
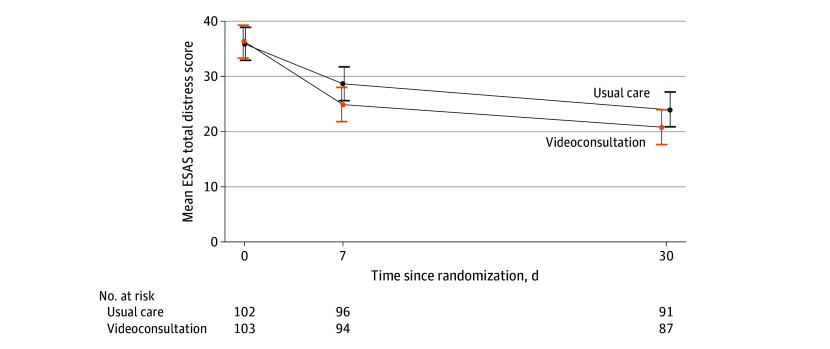
Primary Outcome of Total Symptom Distress Measured by the Edmonton Symptom Assessment Scale (ESAS) Mean scores were estimated using linear mixed effects models. The ESAS score ranges from 0 to 90; lower scores indicate less symptom distress. Whiskers indicate 95% CIs.

Figure 2 and Table 2 show intervention and usual care mean (SE) ESAS total distress scores at baseline, day 7, and day 30. On day 7, the mean (SE) ESAS score change from baseline was −11.4 (1.5) points in the intervention group and −7.3 (1.5) points in the control group; the between-group difference in change in ESAS scores was not statistically significant (Westfall *d*, −0.28; 95% CI, −0.56 to 0.01; *P* = .055). The intervention group’s mean (SE) day 30 ESAS score was not significantly different from that of the usual care group’s (20.8 [1.6] points vs 24.1 [1.6] points; between-group difference in change: Westfall *d*, −0.24 [95% CI, −0.53 to 0.05]; *P* = .09); from baseline to day 7 compared with baseline to day 30, there was a greater decrease in the intervention group than in the usual care group. The mean (SE) day 7 between-group difference in ESAS score of −4.2 (2.2) points exceeded the criteria for a minimal clinically important difference of more than 3 to 4 points,^51^ and the day 30 difference of −3.7 (2.2) points met these criteria. In a sensitivity analysis in which baseline covariates were included in the model (eTable 7 in Supplement 2), the intervention group’s mean (SE) ESAS score at day 7 was significantly different from that of the usual care group’s score (24.5 [1.3] points vs 28.5 [1.3] points; mean [SE] between-group difference in change, −4.0 [1.8] points; Westfall *d*, 0.33 [95% CI, 0.04-0.62]; *P* = .02). The mean (SE) day 7 between-group difference in ESAS score of −4.0 (1.8) points met the criteria for a minimal clinically important difference of 3 to 4 points.

**Table 2.** Change in Symptom Distress and Quality-of-Life Outcomes From Baseline to Day 30 in the Intervention Group vs Usual Care Group

Days after baseline	Intervention			Usual care			Between-group difference in change from baseline^a^		
	Participants, No.	Score, mean (SE)	Change from baseline, mean (SE)	Participants, No.	Score, mean (SE)	Change from baseline, mean (SE)	Mean (SE)	Effect size, Westfall *d* (95% CI)^b^	*P* value^c^
**ESAS total distress score^d^**									
0	103	36.3 (1.5)	NA	102	35.9 (1.5)	NA	NA	NA	NA
7	94	24.9 (1.5)	−11.4 (1.5)	96	28.7 (1.5)	−7.3 (1.5)	−4.2 (2.2)	−0.28 (−0.56 to 0.01)	.055
30	87	20.8 (1.6)	15.5 (1.6)	91	24.1 (1.6)	−11.9 (1.6)	−3.7 (2.2)	−0.24 (−0.53 to 0.05)	.09
**PROMIS global physical health *T* score^e^**									
0	105	35.1 (0.9)	NA	104	34.6 (0.9)	NA	NA	NA	NA
7	94	38.6 (0.9)	3.5 (0.8)	95	38 (0.9)	3.3 (0.8)	0.2 (1.2)	0.02 (−0.24 to 0.28)	.87
30	88	40.5 (0.9)	5.4 (0.9)	90	39.4 (0.9)	4.7 (0.8)	0.6 (1.2)	0.07 (−0.19 to 0.34)	.59
**PROMIS global mental health *T* score^e^**									
0	104	45.5 (0.8)	NA	104	43.7 (0.8)	NA	NA	NA	NA
7	95	45.7 (0.8)	0.1 (0.7)	95	44 (0.8)	0.3 (0.7)	−0.1 (1.0)	−0.02 (−0.28 to 0.24)	.88
30	87	45.8 (0.8)	0.2 (0.8)	93	43.9 (0.8)	0.2 (0.7)	0.0 (1.1)	0.00 (−0.26 to 0.27)	.98

Abbreviations: ESAS, Edmonton Symptom Assessment Scale; NA, not applicable; PROMIS, Patient-Reported Outcomes Measurement System.

^a^ Intervention group minus usual care group; change between groups was calculated as the least-squares mean difference for follow-up (days 7 and 30) minus baseline.

^b^ Effect size was calculated as Westfall *d* for the time × group interaction, and for categorical variables, the Westfall *d* equivalent was calculated as a transformation of the *z* statistic and sample size (0.20, small; 0.50, medium; and 0.80, large).

^c^ *P* values are from the time × group interaction term in mixed models.

^d^ Score range, 0-90; lower scores indicate lower symptom distress.

^e^ The PROMIS Global Health-10 physical and mental health mean (SD) score is 50 (10), and range is 0 to 100; higher scores indicate better health.

#### Secondary and Exploratory Outcomes

Table 2 shows patient participants’ PROMIS physical and mental health mean (SE) *T* scores. Baseline intervention vs usual care mean (SE) physical health scores (35.1 [0.9] vs 34.6 [0.9]) were below the adult population mean (SD) of 50 (10) and improved slightly by day 30 (intervention: 40.5 [0.9] points; mean [SE] change, 5.4 [0.9] points; usual care: 39.4 [0.9] points; mean [SE] change, 4.7 [0.8] points), although there was no significant between-group difference in change from baseline (Westfall *d*, 0.07; 95% CI, −0.19 to 0.34; *P* = .59). Baseline intervention vs usual care mental health mean (SE) *T* scores were below the population mean (45.5 [0.8] vs 43.7 [0.8]) and were essentially unchanged by day 30 (45.8 [0.8] vs 43.9 [0.8]; between-group difference in change from baseline: Westfall *d*, 0.00 [95% CI, −0.26 to 0.27]; *P* = .98). Caregiver participants’ QOL, FAMCARE, and burden scores were not significantly different between the groups (eTable 8 in Supplement 2).

There were no significant differences between the intervention vs usual care groups at day 30 in median length of stay (4.0 days [IQR, 2.0-6.0 days] for both groups) (Table 1) or mean (SE) number of hospital readmissions (0.20 [0.06] vs 0.14 [0.04]; *P* = .44) or emergency visits (0.18 [0.06] vs 0.17 [0.05]; *P* = .84) (Table 3). Video consultation participants had a median length of stay of 2.0 days (IQR, 2.0-3.0 days) before and 1.0 days (IQR, 0.0-3.0 days) after consultation (Table 1).

**Table 3.** Resource Use From Discharge to Day 30 in the Intervention Group vs Usual Care Group

Outcome	Time after baseline, d	Intervention		Usual care		Relative rate (95% CI)^a^	*P* value^b^
		Participants, No.	Mean (SE), No.	Participants, No.	Mean (SE), No.		
Emergency visits	30	88	0.18 (0.06)	90	0.17 (0.05)	1.02 (0.87-1.18)	.84
Hospital readmissions	30	90	0.20 (0.06)	91	0.14 (0.04)	1.06 (0.91-1.23)	.44

^a^ The usual care group was the reference category.

^b^ *P* values are from the group term in zero-inflated Poisson models.

A mean (SE) of 77.7% (4.3%) of patients at baseline and day 30 and of caregivers at day 30 reported feeling completely or quite a bit heard and understood. However, at baseline, a mean (SE) of only 65.1% (5.1%) of caregivers felt completely or quite a bit heard and understood (eTable 9 and eFigure 2 in Supplement 2).

## Discussion

In this RCT among Black or African American and White chronically ill hospitalized adults, culturally based specialist palliative care video consultation was not associated with statistically significant reduced symptom distress compared with usual care, but there was a clinically meaningful difference (ESAS score change of 3-4 points) between groups.^51^ Contrary to our hypotheses, intervention participants’ QOL and resource use (secondary outcomes) also were not improved. To our knowledge, this is one of the first culturally based specialty palliative care video consultation interventions for inpatients in small, rural hospitals in the deep South, few of which have access to palliative care services.^7^ Increasing rural outpatients’ access to palliative care via telehealth is not new^2,9,13^; however, telehealth for inpatients has been underdeveloped and understudied.^22^ The COVID-19 pandemic surge motivated inpatient palliative care via telehealth to conserve personal protective equipment and to promote goals-of-care conversations for intensive care unit patients near death.^11,22,25^ In contrast, our study, conceived prior to the pandemic, tested culturally based video consultation by palliative care specialists to increase both acceptability and access for rural hospitalized older adults. Our goal was to eliminate disparities due to rurality.^7^ While our finding of a clinically meaningful reduction in symptom distress is promising, future implementation can only be realized if accompanied by changes in telehealth infrastructure, policy, clinical, administrative, and financial (payment) incentives.^20,62^ A positive step in that direction is that as of March 2025, the Full-Year Continuing Appropriations and Extensions Act, 2025^63^ passed and will continue through September 30, 2025, extending the flexible telehealth regulations and reimbursement for rural areas with health professional shortages that were instituted during the pandemic.

However, increasing palliative care access via technology alone is insufficient to gain acceptance in rural areas.^28,64^ Small, rural communities, especially those with predominantly racial and ethnic minority populations, have unique cultures.^3^ Leveraging multiple community-engaged strategies to develop our intervention and to partner with local CAB members as advisors and recruitment ambassadors^34,38,41,46^ was essential to getting local community buy-in when introducing potentially beneficial but unfamiliar services such as palliative care.

Our secondary patient QOL and resource use outcomes did not differ significantly at days 7 or 30 compared with usual care, unlike the conclusions from numerous reviews of palliative care studies in cancer^65^ and noncancer^66^ diseases.^31^ There are several plausible explanations for these differences. First, we investigated the short-term effects of a relatively brief inpatient consultation intervention, which was not as robust as the longitudinal outpatient interventions that demonstrated QOL improvements 3 to 6 months after enrollment.^31^ Second, our intentionally broad eligibility criteria may have identified a heterogenous sample that was not sick enough to show improved QOL or reduced resource use and short-term improved symptom distress. However, an American Hospital Association report^67^ and a study on rural hospitals^68^ identified that rural inpatients tended to be older, sicker, and poorer compared with national averages and that most older patients preferred to remain in the local rural hospital rather than be transferred. Furthermore, acute care patients transferred from rural hospitals to larger tertiary centers,^69^ where palliative care teams are common, tended to be younger and healthier.^70,71^ Our sample’s baseline characteristics were consistent with moderate to severe illness severity. The mean (SD) Charlson Comorbidity Index score of 4.6 (2.7) corresponds to a moderate to high illness severity, and 35.9% of patients had a PPS score less than 70%. A PPS score of less than 70% has a positive predictive value of 60% to 80% for 6-month mortality (depending on disease), and patients with these scores are generally considered hospice eligible.^59^ Hence, further research is warranted to evaluate the active ingredients of inpatient palliative care necessary to impact QOL and resource use.^70,72^

### Strengths and Limitations

Important strengths of this study include that the substitution of virtual options to include CAB members as recruitment ambassadors maintained the spirit of engaging community members vs researchers^38^ as first contact. This approach may have contributed to the overall 30% participation rate and a higher proportion of Black or African American patients relative to the local hospitals’ census.^73,74^ Additionally, intervention fidelity was excellent, supporting future intervention reproducibility.

Our study also has numerous limitations. First, the potential for selection bias, as the abundance of patients with COVID-19 limited the admissions of typical patient types and led to visitor restrictions, limited our in-person CAB member recruitment approach and contributed to our inability to reach our original recruitment goal. Second, our results may not be generalizable beyond southeastern US non-Hispanic Black or African American or White populations. Third, without an attention control group, we cannot rule out that the additional 1 to 2 hours of attention that the intervention participants received during the video consultation could explain the clinically meaningful reduced symptom distress.

## Conclusions

In this RCT, possibly the first prospective, multisite, culturally based palliative care video consultation intervention for hospitalized rural Black or African American and White older adults in the US was associated with clinically meaningful but not statistically significant between-group differences in symptom distress, and QOL and resource use were not affected compared with usual care control participants. These findings warrant further investigation of the effectiveness of culturally based palliative care via video consultation in reducing care disparities for inpatients treated in small rural hospitals without access to palliative care services.

## Notes

Supplement 1.
Trial Protocol

Supplement 2. **eAppendix 1.** EMR Screening for Medical Conditions **eTable 1.** Study Sites **eTable 2.** Culturally Based Consultation Guidelines Linked to NCP Consultation Guidelines **eAppendix 2.** Palliative Care Consultation Documentation Template **eAppendix 3.** Community Tele-Pal Videoconsultation Fidelity Monitoring Process **eTable 3.** Characteristics of Patients Who Consented vs Declined **eTable 4.** Caregiver Baseline Demographics and Outcomes **eTable 5.** Comparison of Demographics of Intervention Patients Who Completed and Did Not Complete Videoconsultation **eTable 6.** Patient Satisfaction With Videoconsultation Technology and Process **eTable 7.** ESAS Total Symptom Distress, Intervention vs Usual Care, Adjusted for Baseline **eTable 8.** Caregiver Burden, QOL, and FAMCARE From Baseline to 30 Days, Intervention vs Usual Care **eTable 9.** Caregiver Heard and Understood From Baseline to 30 Days, Intervention vs Usual Care **eFigure 1.** Patient Satisfaction With Technology **eFigure 2.** Patients Feeling Heard and Understood

Supplement 3.
Data Sharing Statement
