# Supplementary material for: Palliative Video Consultation and Symptom Distress Among Rural Inpatients: A Randomized Clinical Trial
Source: JAMA Netw Open. 2025 Jul 9;8(7):e2519426. doi: 10.1001/jamanetworkopen.2025.19426 (PMC12242707; doi:10.1001/jamanetworkopen.2025.19426)
Supplement: Supplement 1. — Trial Protocol [file jamanetwopen-e2519426-s001.pdf]

Protocol Change Amendments for A Community Developed, Culturally-Based Palliative Care Tele-Consult Program for African American and White Rural Southern Elders with A Life Limiting Illness

UAB IRB-300002420

Note. Grant funding began in 2018, trial began enrolling in 6/2020, and the protocol paper was published in 7/2020)

Protocol Version	Protocol Date	Summary of Changes
1	1/30/19	Not applicable- original protocol
2	4/12/19	Add Individual Authorization Agreement for Aiken Regional Medical Center (including copy of FWA)
3	10/4/19	Updated recruitment procedures at University of Alabama at Birmingham: Added breast research specialists who are embedded within the breast cancer clinical team
4	5/17/19	Adding external IRB approval (Russell Medical Center); included copy of approval letter from RMC
5	6/24/19	Add Individual Authorization Agreement for Highland Community Hospital
6	7/3/19	Add external IRB approval (Northwestern University); included copy of approval letter from NU
7	7/23/19	Add Knowledge of Attitudes, Beliefs, Context, Decision-Making Style and Environmental Resources of Southern, Rural African-American Patients with Serious Illness and their Families (ABCDE) and Confidence in Changing Practice
8	9/28/19	Amendment and sent to IRB (9/28/19) – included CG/PT consent forms; two bereavement surveys; addition of six study coordinators and Drs. Susan McCammon/Veronica Tassin
9	10/24/19	Adding Forrest General and Highland Community hospitals as "performance sites)
10	2/3/20	Add REALM SF as data collection instrument<br>Extend caregiver eligibility age to 18+ years vs. current 21+ years of age
11	2/14/20	Added Dr. Rodney Tucker back to protocol with updated CITI GCP initial training
12	3/10/20	Added Melissa Mayo (Highland) and Shella McCormick (Aiken) as coordinating study coordinators; add Telehealth Technology Acceptance Survey
13	4/14/20	Add Shena Gazaway (Augusta University), SMART IRB Reliance between UAB and Augusta University
14	6/8/20	Add NIH/NINR Diversity Supplement
15	6/9/20	Add Caregiver telephone consent
16	6/12/20	Uploading Russell Medical Center Approval Memo
17	7/27/20	Add Marlee Duffie (RMC) as study coordinator
18	8/14/20	Remove the "surprise question" as an eligibility question
19	1/25/21	Uploaded DSMC Chair (Dr. Markland) letter after the first DSMC meeting
20	2/18/21	Add Felicia Underwood (UAB study manager)
21	3/3/21	Add signature line option on Patient and Caregiver consent forms for participants to indicate whether or not they agree to be contacted for additional studies related to the parent study (i.e. Dr. Gazaway's diversity supplement); also revised the Caregiver telephone script to include this language.
22	5/5/21	Removal of Augusta University as a relying site

23	6/2/21	Add Christiana Ekelem (Research Intern)
24	8/23/21	Add Anderson Regional Medical Center and MS study site
25	11/23/21	Add Dr. Lindsey Prewitt (MS Site PI) and Lisa Massey (MS CSC)
26	6/30/22	Add Dr. McElwain and Lori Blansett, NP to the protocol; remove Emily Malone as personnel
27	8/4/22	Reduce age criteria to 55 years
28	9/14/22	Add Christiana Ekelem as UAB employee
29	1/17/24	Add Vantrice Heard and Heather Shelton to the protocol in IRAP

- You are applying for IRB review of the research described in this form.
- To avoid delay, respond to all items in order and include all required approvals and documents. For more tips, see the UAB IRB website.
- To complete the form, click the underlined areas and type or paste in your text; double-click checkboxes to check/uncheck.
- All responses should be Times New Roman, Bold, and Underlined.
- Submit all materials to AB 470, 701 20th Street South, Birmingham, AL 35294-0104.

Indicate the type of review you are applying for:

☒ Convened (Full) IRB ***-OR-***

☐ Expedited - See the Expedited Category Review Sheet, and indicate the category(ies) here:

☐1 ☐2 ☐3 ☐4 ☐5 ☐6 ☐7

1. IRB Protocol Title: A Community Developed, Culturally-Based Palliative Care Tele-Consult Program for African American and White Rural Southern Elders with a Life Limiting Illness.

2. Investigator and Contact Person

a. Name of Principal Investigator: **Ronit Elk**

Degree(s)/Title: **PhD** BlazerID: **elkronit**

Dept/Div: **Medicine/Geriatrics, Gerontology and Palliative Care**

Mailing Address: **1720 2nd Avenue South; Birmingham, AL** UAB ZIP: **35294-2041**

Phone: **205-996-1702** Fax: **205-975-8173** E-mail: **relk@uabmc.edu**

b. Name of Contact Person: **Kristen Allen-Watts** Title: **Post-doctoral employee**

Phone: **205-975-0864**

E-mail: **krallen@uab.edu** Fax: **205-975-8173**

INVESTIGATOR ASSURANCE STATEMENT & SIGNATURE

By my signature as Principal Investigator, I acknowledge my responsibilities for this Human Subjects Protocol, including:

- Certifying that I and all key personnel comply with reporting requirements of the UAB Conflict of Interest Review Board;
- Certifying that the information, data, and/or specimens collected for the research will be used, disclosed and maintained in accordance with this protocol and UAB policies;
- Following this protocol without modification unless (a) the IRB has approved changes prior to implementation or (b) it is necessary to eliminate an apparent, immediate hazard to a participant(s);
- Verifying that all key personnel listed on the protocol have completed initial IRB training and will complete continuing IRB training as required;
- Verifying that all personnel are licensed/credentialed for the procedures they will be performing, if applicable;
- Certifying that I and all key personnel have read the *UAB Policy/Procedure to Ensure Prompt Reporting of Unanticipated Problems Involving Risks to Subjects or Others to the IRB, Institutional Officials, and Regulatory Agencies* and understand the procedures for reporting;
- Applying for continuing review of the protocol at least annually unless directed by the IRB to apply more frequently;
- Conducting the protocol as represented here and in compliance with IRB determinations and all applicable local, state, and federal law and regulations; providing the IRB with all information necessary to review the protocol; refraining from protocol activities until receipt of initial and continuing formal IRB approval.

Signature of Investigator: Ronit Elk **Date:** 1.30.19

3. Protocol Personnel

Including the PI, list all key personnel (each individual involved in the design and conduct of this protocol). See the Key Personnel Flowchart.

Complete the UAB (3.a.) and non-UAB (3.b) tables, as applicable. Use the checkboxes to show each individual's role, whether the individual has financial interests as defined by the UAB CIRB, and briefly describe the individual's protocol responsibilities and qualifications to perform those responsibilities. **Insert additional rows as needed.**

FDA: For studies involving investigational drugs, list all investigators who will be listed on FDA Form 1572 and include a copy of the 1572. Send the IRB a copy of Form 1572 any time you update the form with the FDA.

a. UAB Personnel (includes UAB affiliates and Children's of Alabama personnel)

Name, Degree, and Dept.	Blazer ID	Role	Financial Interest?*	Protocol Responsibilities and Qualifications (indicate if this person obtains consent)
Name: **Ronit Elk**<br>Degree: **PhD**<br>Department: **Medicine**	**elkronit**	Principal Investigator (Multiple PIs)	☒No<br>☐Yes	**Professor, Dept. of Geriatrics, Gerontology and Palliative Care.**<br>• **Responsible for all facets of this research, ranging from fidelity to the research design to fiscal management.**<br>• **Will keep the effort focused and on schedule, and facilitate bridging between investigators, PC physicians, and study staff, including training of CAG, staff and PC physicians**<br>• **Will also ensure fidelity to protocol, collaborate with the hospitals, report to the community, present study findings, and write final results.**
Name: **Marie Bakitas**<br>Degree: **DNSc, CRNP,NP-C, AOCN,ACHPN,FAAN**<br>Department: **Nursing**	**mbakitas**	Principal Investigator (Multiple PIs)	☒No<br>☐Yes	**Professor & Marie L. O' Koren Endowed Chair, UAB School of Nursing Associate Director, UAB Center for Palliative and Supportive Care.**<br>• **Will ensure that all aspects of the study design are implemented accurately in all 3 sites, particularly focusing on the implementation of an RCT.**<br>• **Will participate in the trainings provided to the PC physicians and to the Community Advisory Board members, and in the monthly team meetings.**<br>• **Drs. Elk and Bakitas will serve as primary authors on their joint publications.**
Name: **Rodney Tucker**<br>Degree: **MD**<br>Department: **Medicine**	**rtucker**	☐Sub-Investigator<br>☐Other	☒No<br>☐Yes	**Associate Professor of Medicine and Director of the UAB Center for Palliative and Supportive Care (CPSC).**<br>• **Will serve as the PC consultant for study patients in Russell Medical Center in Alabama.**<br>• **Will be the study's physician liaison to the hospital and the patients' treatment team.**<br>• **Will also participate in**

				regular team meetings and participate in Community Advisory Group meetings.
Name: Richard Kennedy<br>Degree: MD, PhD<br>Department: Medicine	rekenned	☒Sub-Investigator<br>☐Other	☒No<br>☐Yes	Assistant Professor at UAB Division of Gerontology, Geriatrics, and Palliative Care.<br>• Will develop the study database (REDCap) ensuring ease of data entry and data analysis.<br>• Will also supervise the data analyst who will participate in data entry and analysis.
Name: Kristen A Watts<br>Degree: PhD<br>Department: Medicine	krallen	☐Sub-Investigator<br>☒Other	☒No<br>☐Yes	Postdoctoral Associate/Project Coordinator, Dept. of Medicine.<br>• Will be responsible for implementing the study according to study design and protocol.<br>• Will ensure implementation and running of the study at each of the study sites.<br>• Will supervise the 6 site coordinators, set up monthly team meetings, prepare reports for each meeting, and problem-solve daily issues.
Name: Emily Malone<br>Degree: MPH<br>Department: Medicine	emalone	☐Sub-Investigator<br>☒Other	☒No<br>☐Yes	Project Coordinator, Dept. of Medicine<br>• Will assist in REDCap database development

b. Non-UAB Personnel Relying on UAB IRB - If you are requesting that the UAB IRB serve as the IRB of record for anyone not affiliated with UAB, list these individuals below.

Name and Degree	From Institution with or without own IRB?	Financial Interest?*	Protocol Responsibilities and Qualifications (indicate if this person obtains consent)
Name: Sidney Rhoades<br>Degree: MD<br>Institution: Aiken Regional Medical Center<br>Email: sfrhoadesmd@yahoo.com	☐Has own IRB but requests that UAB IRB serve as IRB of record?- *OR-*<br><br>☒Does not have own IRB and needs to rely on UAB IRB.	☒No<br>☐Yes	Chief Hospitalist, Aiken Regional Medical Center, Aiken, SC.<br>• Will serve as Consortium PI for Aiken Regional Medical Center.<br>• He and his team will refer patients, participate in the consults, and follow up visits.<br>• He will also participate in regular study team meetings (remotely).
Name: Michele Goldhagen<br>Degree: MD<br>Institution: Russell Medical Center<br>Email: mgoldhagen@russellmedcenter.com	☐Has own IRB but requests that UAB IRB serve as IRB of record?- *OR-*<br><br>☒Does not have own IRB and needs to rely on UAB IRB.	☒No<br>☐Yes	Chief Hospitalist, Russell Medical Center, AL.<br>• Will serve as Consortium PI (Chief Hospitalist) of Russell Medical Center.<br>• She and his team will refer patients, participate in the consults, and follow up visits.<br>• She will also participate in regular study team meetings (remotely).

***Financial Interest** – for each individual listed above, answer **Yes** or **No** as to whether the individual or an immediate family member has any of the following:
- An ownership interest, stock options, or other equity interest related to the investigator's institutional responsibilities of any value.
- Compensation greater than $5,000 in the previous two years when aggregated for the immediate family
- Proprietary interest including, but not limited to, a patent, trademark, copyright, or licensing agreement.
- Board of executive relationship, regardless of compensation.
- Any other Financial Interest as defined by the UAB CIRB.

UAB Personnel: If the individual or his/her spouse or dependent child has a Financial Interest, a disclosure has to be made to the UAB CIRB. A completed CIRB evaluation has to be available before the IRB can complete its review.

**Non-UAB Personnel:** If the individual has a Financial Interest, ***include a copy of the report*** ***from his/her own institution's conflict of interest review with this submission to the UAB IRB.***	
**c. Do the investigators listed above include any students using this research for their thesis or dissertation?**<br>☐ No, continue with Item 3.d.<br>☐ Yes, complete the following	
**Student Name**	**Thesis/Dissertation Title**
_____	_____

d. Is the principal investigator a student, fellow, or resident? ☐Yes ☒No

If Yes, complete items below and obtain signature of faculty advisor or supervisor:

Supervisor's Name: _____

Degree(s) / Job Title: _____

Additional Qualifications pertinent to the protocol: _____

Telephone: _____

E-Mail: _____

Signature: ______________________________

e. Describe the principal investigator's activities related to this protocol and provisions made by the PI to devote sufficient time to conduct the protocol: **Dr. Elk will devote 4.0 calendar months/year for 5 years to work on the grant. Dr. Bakitas will devote 2.4 calendar months/year for 5 years to work on this grant.**

f. Is medical supervision required for this research? ☐Yes ☒No

If Yes, who will provide the medical supervision?

☐ PI will provide ***-OR-***

☐ Other:

Name: _____ Telephone: _____

If other than PI, obtain signature of person providing medical supervision:

Signature ______________________________

g. Describe your process for ensuring all key personnel are adequately informed about the protocol and their research-related duties and functions: **All study team members will receive a copy of the study protocol. The roles and responsibilities of each team member will be outlined in the protocol and discussed at team meetings, and the research team will meet regularly to discuss study-related progress.**

4. Funding

Is this protocol funded? ☒Yes ☐No

If No, specify that costs of the protocol will be covered by funds from the UAB department or other source named: _____

If Yes, attach one copy of completed application or request for funding sent to sponsor, and complete a-d.

a. Title of Grant, Contract, or Agreement: **A Community Developed, Culturally-Based Palliative Care Consult Program for African American and White Rural Southern Elders with a Life Limiting Illness.**

b. UAB PI of Grant, Contract, or Agreement: **Ronit Elk, PhD**

c. Office of Sponsored Programs (OSP) Assigned Number: **000521619**

(If not yet available, enter "Pending" and provide upon receipt from OSP.)

d. Sponsor, Funding Route:

(Check and describe all that apply)

(If subaward, list both the funding source and the institution receiving the direct award)

☒ Gov't Agency or Agencies—Agency name(s): **NIH- National Institute of Nursing Research (NINR)**
☐ Department of Defense (DoD): Identify DoD component: _____
☐ Department of Energy (DOE)
☐ Department of Justice (DOJ)
☐ Department of Education
☐ NIH Cooperative Group Trial - Group name: _____
☐ Private Nonprofit (e.g., Foundation) - Name: _____
☐ Industry, investigator-initiated - Name: _____
Describe the funding arrangement: _____
NOTE: The UAB IRB typically only reviews industry-sponsored protocols that are investigator initiated or when the protocol qualifies for expedited review or involves gene therapy.
☐ UAB Departmental/Division Funds—Specify: _____

5. Locations Involved

a. Indicate all performance sites that will provide space, services, or facilities for the conduct of this protocol.
☐ UAB Hospital
☐ UAB Hospital - Highlands
☐ The Kirklin Clinic of UAB Hospital
☐ The Kirklin Clinic at Acton Road
☐ UAB Callahan Eye Hospital
☐ UAB Clinical Research Unit
☐ Children's of Alabama
☐ Birmingham Veterans Affairs Medical Center
☐ Jefferson County Department of Health
☒ Other (i.e., any performance site not listed above, including those covered by subawards related to this protocol) - Describe: **This study will take place in 2 hospitals (Aiken Regional Hospital [SC] and Russell Medical Center [AL]). For this study, all two hospitalists work at the 2 sites (i.e., Aiken Regional Hospital and Russell Medical Center). Whereas thepalliative care specialist (who provide the tele-consult intervention) work at different locations (i.e., UAB).**

NOTE: Documentation of IRB approvals from sites receiving subawards must be received by the UAB OIRB before funding will be released for that subaward.

b. Describe the space, service, or facilities available for the conduct of the research in the performance sites listed in Item 5.a (For research on UAB campus, include building names):
NOTE: Letters of support from each of the hospitals are included in IRB submission (see Miscellaneous Documents).

i. Aiken Regional Hospital; Aiken, SC: Aiken Regional Medical Centers, located in Aiken, South Carolina, is a 245-bed acute care facility offering a comprehensive range of specialties and services. The medical staff includes over 900 skilled healthcare/support professionals, a medical staff of more than 120 multi- specialty physicians, and a team of 230 volunteers. The center provides nearly 50 specialty services through its acute care facility, behavioral healthcare hospital and the cancer care institutes of Carolina. Its services reached in Aiken and its surrounding communities. There are different types of the community outreach in Aiken Regional Medical center, including classes, seminars, support groups, or information about new services.

iii. Russell Medical Center, Alexander City, AL: Russell Medical Center is a not-for-profit, acute care facility serving the needs of east Central Alabama. It is a general medical and surgical hospital in Alexander City, AL, with 80 beds. Survey data for the latest year available shows that 24,827 patients visited the hospital's emergency room. There are 67 physicians affiliated with the hospital, and the hospital had a total of 3,245 admissions. Its physicians performed 835 inpatient and 3,264 outpatient surgeries. Russell Medical Center also expounds upon community health

education services through health screenings, support groups, childbirth classes, self-help programs, and athletic trainers to multiple sports teams in its service area.

c. Is this protocol a clinical trial requiring clinical services at one of the performance sites listed in Item 5.a above? ☐Yes ☒No

If Yes, will any of the services be billed to either participants/their insurance or to the study account through the Hospital Billing Office (PFS) or the HSF Billing Office (MSO)? ☐Yes ☒No

If Yes, submit a Full Fiscal Approval Process (FAP)-designated unit submission to s complete a FAP submission and send to fap@uab.edu. For more on the UAB FAP requirements, go to FAP - SiteMinder Processes.

d. Is this a field study? ☒Yes ☐No

If Yes, describe the community and include information about how the community will be involved in the design, implementation and analysis of the research. This would include focus groups, training local facilitators/community health advisors: **CAG have not been identified yet; however, they will be added via Project Revision/Amendment Form (PRAF) when available. CAGs will be comprised of AA and W (8, with equal numbers of AA and W) community members at each study hospital (one each in South Carolina and Alabama).**

e. Has this protocol been rejected or disapproved by another review board (another IRB, similar review board, or departmental review committee(s)) that authorizes the use of its patient populations? ☐Yes ☒No

If Yes, provide name(s) of the review board(s) and reason(s) not approved: ______

Attach copies of the disapprovals.

NOTE: If this protocol is subsequently rejected or disapproved by another review board, promptly notify UAB IRB.

f. Will the protocol be conducted at or recruit participants from the Birmingham Veterans Affairs Medical Center (BVAMC)? ☐Yes ☒No

If Yes, describe the involvement of the BVAMC: ______

Attach the VA IRB approval and VA IRB-stamped consent form(s), if applicable.

NOTE: See the BVAMC section of the IRB Guidebook for more information.

g. Will the protocol be conducted at or recruit participants from the Jefferson County Department of Health (JCDH)? ☐Yes ☒No

If Yes, describe the involvement of the JCDH and list the JCDH clinics being used: ______

Attach the JCDH Research Review Panel approval, if applicable.

NOTE: Human subjects research conducted at certain JCDH clinics requires review by the JCDH Research Review Panel. See the JCDH section of the IRB Guidebook for more information.

6. Clinical Trial

Does this protocol meet the following definition of a clinical trial? ☒Yes ☐No

**A research study in which one or more human subjects are prospectively assigned to one or more interventions (which may include placebo or other control) to evaluate the effects of those interventions on health-related biomedical or behavioral outcomes. For more information, see the full definition of clinical trial here.*

If Yes, you will need to fulfill the following requirements (regardless of funding):

a. All key personnel must complete the Good Clinical Practices (GCP) training. For information on this requirement, visit the IRB website here.

b. This protocol must be registered on ClinicalTrials.gov. Provide the National Clinical Trial (NCT) identifier number: NCT03767517

If you have any questions regarding registering a study on ClinicalTrials.gov, email the UAB Center for Clinical and Translational Science at ccts@uab.edu.

7. Multi-Site Studies

a. Is this a multi-site study with the UAB investigator as the lead investigator? ☒Yes ☐No

b. Is this a multi-site study with UAB as a coordinating site? ☒Yes ☐No

c. If Yes to a or b, describe the management of information obtained in multi-site research that might be relevant to the protection of participants. Include, at a minimum, how the following items are managed:

- IRB approvals from other sites
- Unanticipated problems involving risks to participants or others. (For example, if there is an unanticipated problem involving risks to participants or others, which site is responsible for reporting it?)
- Interim results
- Protocol modifications

UAB IRB will serve as the IRB of record for both hospitals. Risk to patients in this study is minimal (see consent form). Regular meetings will take place between the PIs, the palliative care physicians and the hospitalists throughout the process. The protocols will be modified only following discussion with all groups and the modifications submitted to UAB IRB for Review.

8. Drugs

Will any drugs or supplements be *used or studied* in this protocol? ☐Yes ☒No

If Yes, attach the completed Drug Review Sheet.

9. Devices

a. Will any devices be *studied* in this protocol? ☐Yes ☒No

b. Will any *not FDA-approved* devices be *used or studied* in this protocol? ☐Yes ☒No

If Yes to a or b, attach the completed Device Review Sheet.

10. Special Approvals

a. Does this protocol involve the use of radioisotopes? ☐Yes ☒No

If Yes, attach documentation of approval from the Radiation Safety Division.

b. Does this protocol include patients with contagious infections (e.g., mumps, measles, chickenpox, TB, meningitis)? ☐Yes ☒No

If Yes, attach documentation of approval from the Infection Control Committee of the appropriate facilities.

c. Does this protocol involve obtaining remnant biopsy or surgical material from the Department of Pathology or any other source? ☐Yes ☒No

If Yes, attach documentation of approval from the entity or individual providing the materials (e.g., the UAB Division of Anatomic Pathology Release of Pathologic Materials).

d. Does this protocol require obtaining any remnant clinical laboratory specimens, body fluids, or microbiological isolates from the Department of Pathology or any other source? ☐Yes ☒No

If Yes, attach documentation of approval from the entity or individual providing the materials (e.g., the UAB Division of Laboratory Medicine Release of Pathologic Materials).

e. Does this protocol use stored (existing) specimens from a repository? ☐Yes ☒No

If Yes, attach documentation of approval for use of specimens, and describe how existing specimens are labeled: ______

11. Use of Specimens

Does this protocol involve the collection of specimens? ☐Yes ☒No

If Yes, complete 11.a-11.h.

If No, skip to Item 12.

a. How will specimens be obtained, processed, distributed, and stored? _____

b. How will specimens be labeled (e.g., unique identifier, medical record number, Social Security number, name, date of birth)? _____

c. How will clinical data associated with the specimens be collected and stored? _____

d. What participant-identifying information will be collected and linked to the specimens? _____

e. What steps will be taken to maximize the confidentiality of linked identifiers? For example, procedures could include using a password-protected computer database to link identifiers, with limited personnel knowledgeable of the password, or coded identifiers released without the ability to link to clinical data (also called “stripped” or “anonymized” specimens). _____

f. Is genetic testing planned as part of this protocol? ☐Yes ☐No

If Yes, describe the planned genetic testing here. _____

g. Will specimens be stored for future use? ☐Yes ☐No

If Yes, indicate whether they will be used for the disease under study in this protocol or research on other diseases. _____

h. Will specimens be shared with other investigators in the future? ☐Yes ☐No

If Yes, answer i. and ii.

i. What identifiers, clinical information and demographic information will be shared; or will the specimens be stripped of identifiers (i.e., anonymized)? _____

ii. Outline your procedure for assuring IRB approval for release and use prior to release of specimens. _____

NOTE: Investigators who receive and/or use these specimens must document approval from the appropriate IRB(s) before the specimens may be released.

12. Gene Therapy

Does this protocol involve gene therapy or administering recombinant materials to humans? ☐Yes ☒No

If Yes, submit the Gene Therapy Project Review Panel Report ***-OR-*** the Protocol Oversight Review Form For Clinical Vaccine Trials, as applicable.

13. HIPAA Privacy and Security

Will the PI or others obtain, review, or make other use of participants' “protected health information” (i.e., information, whether oral or recorded in any form or medium that (a) is created or received by a health care provider and (b) relates to past, present, or future physical or mental health or condition of an individual; or provision of health care; or payment for provision of heath care)? ☒Yes ☐No

If Yes, complete Items 13.a-13.f.

If No, skip to 14.

a. Will the data/information be stored or managed electronically (on a computer)? ☒Yes ☐No

b. Is the principal investigator requesting that the UAB IRB waive patient HIPAA authorization from another institution or entity (e.g., insurance company, collaborating institution)? ☐Yes ☒No

If Yes, attach copies of the privacy notices from each institution/entity, and provide the name of each institution/entity: _____

c. Indicate which of the entities would provide health information for this protocol, maintain health information as it was collected for this protocol, and/or store health information after it has been collected for this protocol.

☐ UAB Hospital or UAB Hospital - Highlands
☐ The Kirklin Clinic of UAB Hospital or Acton Road (and/or associated clinics)
☐ UAB Callahan Eye Hospital
☐ Children's of Alabama
☐ Jefferson County Department of Health
☐ School of Dentistry
☐ School of Health Professions
☒ School of Medicine
☐ School of Nursing
☐ School of Optometry
☐ University of Alabama Health Services Foundation
☐ UAB Health Centers
☐ Viva Health
☐ Ophthalmology Services Foundation
☐ Valley Foundation
☐ Medical West - UAB Health System Affiliate
☐ None - **If None, skip to Item 14.**

d. Indicate any information systems that will be the sources of information used for the protocol.

☐ A system maintained centrally by UAB Health System (these include the following: HealthQuest for registration, billing, and patient administration; PowerInsight (clinical data warehouse); Cerner IMPACT for PowerNotes for meds, Lab, Radiology, UED, Surgery

NOTE: If a researcher needs information in a specified format or a specified time, the researcher must confirm with the unit who can supply the information/service that the request can be met before writing the information/service into the research protocol. In addition, the researcher must be aware that these services may have a cost attached that should be considered in the research budget.

To request access to clinical systems for research purposes, visit https://www.oneuabmedicine.org/web/hsis/technical-support, click "Accounts Request" and complete the form indicating access for research purposed.

☒ Another system on a UAB server - Describe: **UAB has Cisco based infrastructure for teleconferencing.**

e. Indicate which of the listed identifiers will be accessed, associated and/or linked with the protected health information (PHI) used for this protocol.

☒ Names
☒ Geographic subdivisions smaller than a state
☒ Elements of dates (except year) related to an individual
☒ Telephone numbers
☐ Fax numbers
☐ Email addresses
☐ Social security numbers
☒ Medical record numbers
☐ Health plan beneficiary numbers
☐ Account numbers
☐ Certificate/license numbers

☐ Vehicle identifiers and serial numbers
☐ Device identifiers and serial numbers
☐ Biometric identifiers
☐ Web universal resource locators (URLs)
☐ Internet protocol address numbers
☐ Full-face photographic images
☐ Any other unique identifying number - Describe: ______
NOTE: Codes are not identifying as long as the researcher cannot link the data to an individual
☐ None - **If None, skip to Item 14.**

f. Choose one plan to describe your use of the personal health information:
☐ The data collected meet the specifications for a "limited data set" (LDS)
-If the LDS will leave the covered entity or will be received from another covered entity you will need a Data Use Agreement

☒ Research staff will obtain authorization from each participant to use the information
-Include the HIPAA Authorization form, complete except for participant name and IRB protocol number, as the final page of the consent form

☐ PI requests waiver of authorization to use the information
-Attach Waiver of Authorization and Informed Consent form

PROPOSED RESEARCH

- The IRB will not accept grant applications and/or sponsor's protocols in lieu of the items as outlined below.
- Do not separate responses from items. Instead, insert your response to each item below the item, keeping the information in the order of this form.

14. Purpose - in nontechnical, lay language

a. Summarize the purpose and objectives of this protocol in one short paragraph. **Rural patients with life-limiting illness are at very high risk of not receiving appropriate care due to a lack of health professionals, long distances to treatment centers, and limited PC clinical expertise. Secondly, although culture strongly influences people's response to diagnosis, illness and treatment preferences, culturally-based care models are not currently available for most seriously-ill rural patients and their family caregivers. Lack of sensitivity to cultural differences may compromise PC for minority patients. The purpose of this study is to compare a culturally-based Tele-consult program to usual hospital care to determine whether a culturally- based PC Tele-consult program leads to lower symptom burden in hospitalized African American and White older adults with a life-limiting illness.**

b. Describe how outcomes will be measured for this protocol. **Patient and caregiver symptom burden and quality of life will be measured by the study coordinator using standardized questionnaires.**

15. Background - in nontechnical, lay language

Summarize in 2-3 paragraphs past experimental and/or clinical findings leading to the design of this protocol. Include any relevant past or current research by the PI. For drug and device studies, summarize the previous results (i.e., Phase I/II or III studies).

Background and Scientific Premise of the Study

Palliative care consultations for inpatients have successfully identified unrecognized symptoms and unmet needs,[41-45] have been associated with lower ICU use,[22,24] fewer ICU deaths,[46] improved care processes, and higher rates of goals of care documentation.[47-49] The triple threat of rural geography, racial inequities, and older age hinders access to high quality palliative care for a

significant proportion of Americans. In a state-by-state report card,[50] the Southeastern US, where a significant proportion of the population is rural and African American (AA), palliative care access was ranked the lowest in the nation. Rural patients with life-limiting illness are at very high risk of not receiving appropriate care due to a lack of health professionals (nearly two thirds of rural US counties are designated health professional shortage areas),[2] long distances to treatment centers,[3] and limited palliative care clinical expertise.[4] Even when palliative and hospice services are available, AA, compared to W are more likely to receive medically-ineffective, poor quality and high cost care, due to general mistrust of health care providers and a fragmented health care system that is generally insensitive to cultural differences that can guide treatment choices.[51-56]

AA and Ws end-of-life (EOL) cultural values differ. Despite proven effectiveness, numerous studies have shown that AAs underutilize palliative and hospice care.[48,49,57-60] Three reasons have been suggested: 1) A lack of exposure to hospice or palliative care information,[61-63] 2) AAs' values of EOL care differ. Historically EOL care has been rooted in W middle class cultural and religious values;[1,62] a very different frame of reference, value system, and life experience compared with many AA.[64] Where middle class W may emphasize individual choice; AA values support family-centered decisionmaking.[65] AAs' often value faith, spiritual beliefs and guidance of a spiritual leader,[62] especially as they cope with illness and make treatment decisions.[66,67] Yet physicians rarely ask patients about their spirituality.[68] AA's reliance on hope[69] and faith in God's healing power,[70] can be at odds with physicians' felt need to share a terminal prognosis.[57] 3) Historical and social factors including slavery, racism, medical experimentation and exploitation,[57,71] and ongoing racism and microaggression,[72] have left a deep seated legacy of mistrust in the AA community.[73-75] A recent report [76] found that, AAs and Ws are "worlds apart" in their perceptions of racial equality and gaps in household income. This is even more strongly felt in the "Deep South" where slavery was promoted.[77] A recent study[78] found AAs are more likely than other racial groups to believe physicians don't care about them as individuals, and are less likely to trust their physicians' judgment and personal competence.

A community-developed, culturally-based intervention can promote palliative care acceptability. Culture fundamentally shapes how individuals make meaning out of illness, suffering, and dying,[79] and strongly influences their responses to diagnosis, illness, and treatment preferences.[79-81] Consideration of the patient and their family's culture is essential in palliative care; lack of sensitivity to; lack of respect for cultural differences may compromise EOL care for minority patients.[1] Yet culturally appropriate models of care that take into consideration the diverse cultural preferences of seriously-ill rural patients and their family caregivers are not currently available in the US. There is an urgent need for research that emphasizes varying EOL care cultural preferences.[1,6,7,65,82-84] Community-based participatory research (CBPR), a public health method in which academia and the community form a joint partnership to address community issues, has been found to address health disparities [85,86] and result in demonstrable positive health outcomes.[87] CBPR builds on community strengths. The community is integral to all phases of the research, for the mutual benefit of all partners involved in the process, and in disseminating findings and knowledge to all partners.[88] Consumer input has been demonstrated to enhance both the quality and acceptability of interventions.[89] We believe that this proposal is the first to employ this methodology to develop and test the efficacy of a culturally-based palliative care tele-consult program for hospitalized AA and W rural elders and their families.

A Tele-consult program for hospitalized AA and W can reduce symptom burden by improving access to high quality palliative care in rural, underserved areas. Over the last 20 years, tele-health, the remote delivery of health care and sharing of medical knowledge using telecommunication has been used to deliver health care to remote areas for a variety of illnesses.[90-96] Our teams were among the first to conduct large trials of palliative care by tele-health that reduced symptom and caregiver burden and improved quality of life of seriously-ill patients in rural northern New England [22,24,26,27] and in the Deep South states of Alabama, Mississippi,

Georgia, South Carolina.[9,10,13,20,97-99] There have been a few successful, locally-based initiatives to expand palliative expertise to rural areas through community partnerships,[100,101] our team has conducted the majority of these community-based partnership initiatives to create this evidence-base in the US.

The triple threat of rural geography, racial inequities, and older age hinders access to high quality palliative care (PC) for a significant proportion of Americans.[1] Rural patients with life-limiting illness are at very high risk of not receiving appropriate care due to a lack of health professionals,[2] long distances to treatment centers,[3] and limited PC clinical expertise.[4] Although culture strongly influences people's response to diagnosis, illness and treatment preferences,[5] culturally-based care models are not currently available for most seriously-ill rural patients and their family caregivers. Lack of sensitivity to cultural differences may compromise PC for minority patients. The two major public health consequences of these problems are: 1) Access-Rural patients have sub-optimal or no access to PC. Despite significant nationwide growth, access to PC is grossly inadequate for the 60 million US citizens who live in rural or non-metropolitan areas.[6] There is low PC use in rural and minority populations. As a result, rural patients experience significant suffering from uncontrolled symptoms that PC expertise could alleviate. 2) Acceptability-Even when palliative and hospice services are available, African Americans (AA), compared to Whites (W) are more likely to receive medically-ineffective, poor quality care due to a culturally-insensitive health care system and mistrust of health care providers. Making culturally competent PC available for diverse underserved and rural Americans is a national priority.[7] This proposal responds to the urgent need to address racial, ethnic and cultural diversity. Our community-developed, culturally based Teleconsult Intervention, specifically targets the gaps of PC access and acceptability and one of the first culturally-based PC Teleconsult programs developed by and for the rural southern AA and W populations it serves.

Our team has over a decade of experience creating, testing, and developing culturally-based, patient-centered palliative care telehealth interventions, including an NIH-funded pilot study (1R21AG046772-01) that underlies this Tele-consult program. Our pilot project systematically incorporated diverse community stakeholder input on how to best design and deliver culturally-based PC for rural communities. This included:

(1) Engaging a Community Advisory Group and using Community Based Participatory Research for intervention development.
(2) Developing a Culturally-Based Palliative Care Teleconsult Program.
(3) Conducting Palliative care via telehealth.

This background and pilot work showed feasibility and acceptability and served as the basis for the current study. This project can potentially improve the current standard of care in that most rural hospitals do not have access to palliative care and a culturally-based approach; especially for the older African American population. Thus, we will test the efficacy of adding this approach to half of the patients randomized to the intervention vs receiving usual care.

REFERENCES:

1. Krakauer EL, Crenner C, Fox K. Barriers to optimum end-of-life care for minority patients. J Am Geriatr Soc. 2002;50(1):182-190.
2. Bennett KJ, Olatosi B, Probst JC. Health disparities: a rural-urban chartbook. South Carolina: Rural Health Research Center; June 2008 2008.
3. Goodlin S. Palliative care in congestive heart failure. Journal of the American College of Cardiology. 2009;54(5):386-396.
4. Buck HG, Riegel B. The impact of frailty on health related quality of life in heart failure. Eur J Cardiovasc Nurs. 2011;10(3):159-166.
5. Cain C, A S, Elk R, Kagawa-Singer M. Unpacking Culture, Race and Ethnicity: Quality Palliative Care for All. Journal of Pain and Symptom Management. 2017;Under Review.
6. U.S. Census Bureau. 2010 Census Demographic Profiles. 2010. http://www.census.gov/2010census/popmap/. Accessed August 17, 2017.
7. Periyakoil VS, Neri E, Kraemer H. Patient-Reported Barriers to High-Quality, End-of-Life Care: A Multiethnic, Multilingual, Mixed-Methods Study. J Palliat Med. 2016.

8. Elk R. A Community-Developed, Culturally-Based Palliative Care Program for African American and White Rural Elders with a Life-Limiting Illness: A Program By The Community for the Community. Narrat Inq Bioeth. 2017;7(1):36-40.
9. Elk R, Hauser J, Reparaz L, Emanuel L, Levkoff S. Developing a Culturally Tailored Palliative Care Program with Guidance from Rural African American and White Community Members: A Program by the Community for the Community (FR461B). Journal of Pain and Symptom Management. 2016;51(2):372-373.
10. Elk R, Johnson K, Campbell C, Kennard C, Quest T. Innovative Strategies to Address the Unique Cultural Beliefs and Spiritual Perspectives of African American Patients and Families at the End of Life. Journal of Pain and Symptom Management. 2015;49(2):335.
11. Elk R, Emanuel L, Kelly S, et al. A Community Generated Palliative Care Telemedicine Program for Rural African American and White Elders. Step 1: Understanding the Perspectives and Preferences towards End of Life Care of Family Caregivers. (Poster). Paper presented at: Aging and Technology, Palmetto Health2014; Columbia, SC.
12. Bagcivan G, Tucker R, Kvale E, et al. Looking Back, Moving Forward: A Retrospective Review of Care Trends in an Academic Palliative and Supportive Care Program from 2004-2015 (S744). Journal of Pain and Symptom Management. 2017;53(2):434.
13. Gelfman LP, Bakitas M, Warner SL, Kirkpatrick JN, E. GN, Group I-HW. The State of the Science on Integrating Palliative Care in Heart Failure. Journal of Palliative Medicine. 2017;20(6):592-603.
14. Hay CM, Lefkowits C, Crowley-Matoka M, et al. Gynecologic Oncologist Views Influencing Referral to Outpatient Specialty Palliative Care. Int J Gynecol Cancer. 2017;27(3):588-596.
15. Hay CM, Lefkowits C, Crowley-Matoka M, et al. Strategies for Introducing Outpatient Specialty Palliative Care in Gynecologic Oncology. J Oncol Pract. 2017;13(9):e712-e720.
16. Iyer AS, Bakitas M. Early Palliative Care in Advanced Illness: Do Right by Mama. JAMA Intern Med. 2017;177(6):761-762.
17. Sharif R, Zurko JC, Azuero A, et al. Factors Associated With Lower Cost And Healthcare Utilization Among Critically Ill Patients With History Of Chronic Lung Disease Who Received Early Palliative Care Consultation. American Journal of Respiratory and Critical Care Medicine. 2017:A7092.
18. Sipples R, Taylor R, Kirk-Walker D, Bagcivan G, Dionne-Odom JN, Bakitas M. Perioperative Palliative Care Considerations for Surgical Oncology Nurses. Semin Oncol Nurs. 2017;33(1):9-22.
19. Walker DK, Edwards RL, Bagcivan G, Bakitas MA. Cancer and Palliative Care in the United States, Turkey, and Malawi: Developing Global Collaborations. Asia Pac J Oncol Nurs. 2017;4(3):209-219.
20. Wells R, Ejem D, Dionne-Odom J, et al. What's in the "Black Box"? Describing the Focus of Early, Outpatient Palliative Care Consultation Evaluations and Treatment Recommendations for Individuals with Advanced Heart Failure (TH321B). Journal of Pain and Symptom Management. 2017;53(2):327-328.
21. Zubkoff L, Dionne-Odom JN, Pisu M, et al. Developing a "toolkit" to measure implementation of concurrent palliative care in rural community cancer centers. Palliat Support Care. 2017:1-13.
22. Bakitas M, Stevens M, Ahles T, et al. Project ENABLE: a palliative care demonstration project for advanced cancer patients in three settings. J Palliat Med. 2004;7(2):363-372.
23. Bakitas M, Lyons KD, Hegel MT, et al. Effects of a palliative care intervention on clinical outcomes in patients with advanced cancer: the Project ENABLE II randomized controlled trial. Jama. 2009;302(7):741-749.
24. Bakitas M, Lyons KD, Hegel MT, et al. The project ENABLE II randomized controlled trial to improve palliative care for rural patients with advanced cancer: baseline findings, methodological challenges, and solutions. Palliat Support Care. 2009;7(1):75-86.
25. Bakitas M, Lyons KD, Hegel MT, Ahles T. Oncologists' perspectives on concurrent palliative care in a National Cancer Institute-designated comprehensive cancer center. Palliat Support Care. 2013;11(5):415-423.
26. Bakitas M, Dionne-Odom JN, Pamboukian SV, et al. Concurrent Palliative Care for Advanced Heart Failure Patients and Caregivers: Results of the ENABLE CHF-PC Pilot Clinical Trial. In:2014.
27. Bakitas MA, Tosteson TD, Li Z, et al. Early Versus Delayed Initiation of Concurrent Palliative Oncology Care: Patient Outcomes in the ENABLE III Randomized Controlled Trial. J Clin Oncol. 2015;33(13):1438-1445.
28. Marie B, Nicholas D-OJ, V. PS, et al. Engaging patients and families to create a feasible clinical trial integrating palliative and heart failure care: results of the ENABLE CHF-PC pilot clinical trial. BMC Palliative Care. 2017;16(45).
29. Bakitas M, Dionne-Odom JN, Jackson L, Frost J, Bishop MF, Li Z. "There were more decisions and more options than just yes or no": Evaluating a decision aid for advanced cancer patients and their family caregivers. Palliat Support Care. 2017;15(1):44-56.
30. Bakitas MA. On the Road Less Traveled: Journey of an Oncology Palliative Care Researcher. Oncol Nurs Forum. 2017;44(1):87-95.
31. Bakitas M, Dionne-Odom JN. When it comes to death, there is no place like home... Or is there? Palliat Med. 2017;31(5):391-393.
32. Dionne-Odom JN, Kono A, Frost J, et al. Translating and testing the ENABLE: CHF-PC concurrent palliative care model for older adults with heart failure and their family caregivers. J Palliat Med. 2014;17(9):995-1004.
33. Dionne-Odom JN, Azuero A, Lyons KD, et al. Benefits of Early Versus Delayed Palliative Care to Informal Family Caregivers of Patients With Advanced Cancer: Outcomes From the ENABLE III Randomized Controlled Trial. J Clin Oncol. 2015;33(13):1446-1452.
34. Dionne-Odom JN, Hull JG, Martin MY, et al. Associations between advanced cancer patients' survival and family caregiver presence and burden. Cancer Med. 2016.
35. JN D-O, A A, KD L, et al. Family Caregiver Depressive Symptom and Grief Outcomes From the ENABLE III Randomized Controlled Trial. Journal of Pain and Symptom Management. 2016;52(3):378-385.
36. Dionne-Odom JN, Bakitas MA, Zubkoff L. Highlighting Implementation Findings in Early Palliative Care. J Oncol Pract. 2017;13(9):599-600.
37. Dionne-Odom JN, Demark-Wahnefried W, Taylor RA, et al. The self-care practices of family caregivers of persons with poor prognosis cancer: differences by varying levels of caregiver wellbeing and preparedness. Support Care Cancer. 2017;25(8):2437-2444.
38. Clinical Practice Guidelines for Quality Pallative Care, 3rd Edition. National Consensus Project for Quality Palliative Care; 2013.

39. Medicine Io. Approaching Death: Improving Care at the End of Life. Washington, DC: The National Academies Press; 1997.
40. Medicine Io. Dying in America: Improving Quality and Honoring Individual Preferences Near the End of Life. Washington, DC: The National Academies Press; 2015.
41. Abrahm JL, Callahan J, Rossetti K, Pierre L. The impact of a hospice consultation team on the care of veterans with advanced cancer. J Pain Symptom Manage. 1996;12(1):23-31.
42. Bailey FA, Burgio KL, Woodby LL, et al. Improving processes of hospital care during the last hours of life. Arch Intern Med. 2005;165(15):1722-1727.
43. Bascom PB. A hospital-based comfort care team: consultation for seriously ill and dying patients. Am J Hosp Palliat Care. 1997;14(2):57-60.
44. Kuin A, Courtens AM, Deliens L, et al. Palliative care consultation in The Netherlands: a nationwide evaluation study. J Pain Symptom Manage. 2004;27(1):53-60.
45. Manfredi PL, Morrison RS, Morris J, Goldhirsch SL, Carter JM, Meier DE. Palliative care consultations: how do they impact the care of hospitalized patients? J Pain Symptom Manage. 2000;20(3):166-173.
46. Elsayem A, Smith ML, Parmley L, et al. Impact of a palliative care service on in-hospital mortality in a comprehensive cancer center. J Palliat Med. 2006;9(4):894-902.
47. Elliott AM, Alexander SC, Mescher CA, Mohan D, Barnato AE. Differences in Physicians' Verbal and Nonverbal Communication With Black and White Patients at the End of Life. J Pain Symptom Manage. 2016;51(1):1-8.
48. Cohen LL. Racial/ethnic disparities in hospice care: a systematic review. J Palliat Med. 2008;11(5):763-768.
49. LoPresti MA, Dement F, Gold HT. End-of-Life Care for People With Cancer From Ethnic Minority Groups: A Systematic Review. Am J Hosp Palliat Care. 2014.
50. University of Wisconsin Population Health Institute. County Health Rankings 2012. Accessible at www.countyhealthrankings.org. http://www.census.gov/content/dam/Census/library/publications/2015/demo/p60-253.pdf. Accessed August 29, 2016.
51. Puri S. Unequal Lives, Unequal Deaths. In. The End: The New York Times; 2016.
52. Torke AM, Garas NS, Sexson W, Branch WT. Medical care at the end of life: views of African American patients in an urban hospital. J Palliat Med. 2005;8(3):593-602.
53. Boucher NA, Raghavan M, Smith A, Arnold R, Johnson KS. Palliative Care in the African American Community #204. J Palliat Med. 2016;19(2):228-230.
54. Check DK, Samuel CA, Rosenstein DL, Dusetzina SB. Investigation of Racial Disparities in Early Supportive Medication Use and End-of-Life Care Among Medicare Beneficiaries With Stage IV Breast Cancer. J Clin Oncol. 2016.
55. Elk R. The First Step Is Recognizing, Acknowledging, and Respecting the Inequity, Disrespect, and Disregard Our African American Patients Have Experienced. J Palliat Med. 2016;19(2):124-125.
56. Abunafeesa H, Elsayem AF. Cultural diversity and barriers to high-quality end of life care. Ann Palliat Med. 2017;6(2):183-186.
57. Payne R. Racially Associated Disparities in Hospice and Palliative Care Access: Acknowledging the Facts While Addressing the Opportunities to Improve. Journal of Palliative Medicine. 2015;In Press.
58. Rhodes RL, Teno JM, Welch LC. Access to hospice for African Americans: are they informed about the option of hospice? J Palliat Med. 2006;9(2):268-272.
59. Colon M, Lyke J. Comparison of hospice use and demographics among European Americans, African Americans, and Latinos. Am J Hosp Palliat Care. 2003;20(3):182-190.
60. Ludke RL, Smucker DR. Racial differences in the willingness to use hospice services. J Palliat Med. 2007;10(6):1329-1337.
61. Johnson KS, Kuchibhatla M, Tulsky JA. Racial differences in self-reported exposure to information about hospice care. J Palliat Med. 2009;12(10):921-927.
62. Wicher CP, Meeker MA. What influences African American end-of-life preferences? J Health Care Poor Underserved. 2012;23(1):28-58.
63. Hazin R, Giles CA. Is there a color line in death? An examination of end-of-life care in the African American community. J Natl Med Assoc. 2011;103(7):609-613.
64. Baker M. Cultural differences in the use of advance directives: a review of the literature. Afr Am Res Perspect. 2000;6:35-40.
65. Mazanec PM, Daly BJ, Townsend A. Hospice utilization and end-of-life care decision making of African Americans. Am J Hosp Palliat Care. 2010;27(8):560-566.
66. Johnson KS, Elbert-Avila KI, Tulsky JA. The influence of spiritual beliefs and practices on the treatment preferences of African Americans: a review of the literature. J Am Geriatr Soc. 2005;53(4):711-719.
67. True G, Phipps EJ, Braitman LE, Harralson T, Harris D, Tester W. Treatment preferences and advance care planning at end of life: the role of ethnicity and spiritual coping in cancer patients. Ann Behav Med. 2005;30(2):174-179.
68. Ernecoff NC, Curlin FA, Buddadhumaruk P, White DB. Health Care Professionals' Responses to Religious or Spiritual Statements by Surrogate Decision Makers During Goals-of-Care Discussions. Jama Intern Med. 2015;175(10):1662-1669.
69. Kennard C. Undying Hope. Journal of Palliative Medicine. 2015;In Press.
70. Mansfield CJ, Mitchell J, King DE. The doctor as god's mechanic? Beliefs in the Southeastern United States. Social Science & Medicine. 2002;54(3):399-409.
71. Halloway K. Their Bodies, Our Conduct: How Society and Medicine Produce Persons Standing in Need of End of Life Care. Journal of Palliative Medicine.
72. Sue D. Microaggressions in Everyday Life: Race, Gender and Sexual Orientation. Hoboken, New Jersey: John Wiley and Sons; 2010.
73. Freeman HP, Payne R. Racial injustice in health care. N Engl J Med. 2000;342(14):1045-1047.
74. Shrank WH, Kutner JS, Richardson T, Mularski RA, Fischer S, Kagawa-Singer M. Focus group findings about the influence of culture on communication preferences in end-of-life care. J Gen Intern Med. 2005;20(8):703-709.
75. Taxis JC. Attitudes, values, and questions of African Americans regarding participation in hospice programs. J Hospice and Palliat Nursing. 2006;2(2):77-85.
76. Goldberg L. Poll: Doctors Want to Discuss End-of-Life Issues, but Barriers Remain. In. Research & Analysis. http://www.pewtrusts.org/: The Pew Charitable Trusts; 2016.

77. Webster Dictionary.org. "Deep South". 2016; http://www.websterdictionary.org/definition/Deep%20South. Accessed August 17, 2016.
78. Sewell AA. Disaggregating ethnoracial disparities in physician trust. Soc Sci Res. 2015;54:1-20.
79. Kagawa-Singer M, Blackhall LJ. Negotiating cross-cultural issues at the end of life: "You got to go where he lives". JAMA. 2001;286(23):2993-3001.
80. Searight HR, Gafford J. Cultural diversity at the end of life: issues and guidelines for family physicians. Am Fam Physician. 2005;71(3):515-522.
81. Ersek M, Kagawa-Singer M, Barnes D, Blackhall L, Koenig BA. Multicultural considerations in the use of advance directives. Oncol Nurs Forum. 1998;25(10):1683-1690.
82. Lynch S. Hospice and palliative care access issues in rural areas. Am J Hosp Palliat Care. 2013;30(2):172-177.
83. Kwak J, Haley WE. Current research findings on end-of-life decision making among racially or ethnically diverse groups. Gerontologist. 2005;45(5):634-641.
84. Mitchell BL, Mitchell LC. Review of the literature on cultural competence and end-of-life treatment decisions: the role of the hospitalist. J Natl Med Assoc. 2009;101(9):920-926.
85. Chau TS, Islam N, Tandon D, Ho-Asjoe H, Rey M. Using community-based participatory research as a guiding framework for health disparities research centers. Prog Community Health Partnersh. 2007;1(2):195-205.
86. Wallerstein NB, Duran B. Using community-based participatory research to address health disparities. Health Promot Pract. 2006;7(3):312-323.
87. Laveaux D, Christopher S. Contextualizing CBPR: key principles of CBPR meet the indigenous research context. Pimatisiwin. 2009;7(1):1.
88. Israel BA, Eng E, Schulz AJ, Parker EA, Satcher D. Methods in community-based participatory research for health. 1st ed: Jossey-Bass; 1 edition; 2005.
89. Halcomb EJ, Gholizadeh L, DiGiacomo M, Phillips J, Davidson PM. Literature review: considerations in undertaking focus group research with culturally and linguistically diverse groups. J Clin Nurs. 2007;16(6):1000-1011.
90. Thrall JH, Boland G. Telemedicine in practice. Semin Nucl Med. 1998;28(2):145-157.
91. Bjorn P. Rural teletrauma: applications, opportunities, and challenges. Adv Emerg Nurs J. 2012;34(3):232-237.
92. Hess DC, Wang S, Hamilton W, et al. REACH: clinical feasibility of a rural telestroke network. Stroke. 2005;36(9):2018-2020.
93. Steventon A, Bardsley M, Billings J, et al. Effect of telehealth on use of secondary care and mortality: findings from the Whole System Demonstrator cluster randomised trial. BMJ. 2012;344:e3874.
94. Stradling DA. Telestroke: state of the science and steps for implementation. Crit Care Nurs Clin North Am. 2009;21(4):541-548.
95. Taylor DM, Stone SD, Huijbregts MP. Remote participants' experiences with a group-based stroke self-management program using videoconference technology. Rural Remote Health. 2012;12:1947.
96. Forum NQ. Creating a Framework to Support Measure Development for Telehealth. 2017.
97. Elk R, Quest, T Johson, K, Campbell, C, Kennard, C. Innovative Strategies to Address the Unique Cultural Beliefs and Spiritual Perspectives of African American Patients and Families at End of Life. American Association of Hospice and Palliative Medicine; 2015; Philadelphia, Pennsylvania.
98. Elk R, Hauser J, Reparaz L, Emanuel L, Levkoff S. Developing a Culturally Tailored Palliative Care Program with Guidance from Rural African American and White Community Members: A Program by the Community, for the Community (Presentation). March 12-16, 2016, 2016; AAHPM Annual Assembly.
99. JN D-O, A A, KD L, et al. Family Caregiver Depressive Symptom and Grief Outcomes From the ENABLE III Randomized Controlled Trial. Journal of Pain and Symptom Management. 2016;52(3):378-385.
100. Bakitas M, Clifford K, Dionne-Odom J, al e. Rural Palliative Care. In: Ferrell BR, Coyle N, Paice J, eds. Oxford Textbook of Palliative Nursing, Fourth Edition. Oxford: Oxford University Press; 2015:812-822.
101. Kaasalainen S, Brazil K, Kelley ML. Building capacity in palliative care for personal support workers in long-term care through experiential learning. Int J Older People Nurs. 2012(23051621).

16. Participants (Screening and Selection)

a. How many participants are to be enrolled at UAB (if other sites relying on UAB IRB, list the number for each site)?

If multi-site study, total number at all sites/institutions:

We anticipate a total enrollment of 297 participants in the study:
Aiken Regional Medical Center: 188
Russell Medical Center: 109

b. Describe the characteristics of anticipated or planned participants (if multiple groups, repeat list for each group).

Sex: **both**
Race/Ethnicity: **AA and White**
Age: **65+**
Health status: **has at least one of the following health conditions: cancer, cardiac disease, pulmonary disease, neuro-degenerative disease, renal disease, stroke, sepsis, hepatic disease.**

c. From what population(s) will the participants be derived? **Participants for this study will include adults (aged 65+) who are hospitalized. The triple threat of rural geography, racial inequities, and older age hinders access to high quality palliative care for a significant proportion of Americans.**

Rural patients with life-limiting illness are at very high risk of not receiving appropriate care due to a lack of health professionals, long distances to treatment centers, and limited PC clinical expertise.

Describe your ability to obtain access to the proposed population that will allow recruitment of the necessary number of participants: **(1) We have examined the number of patients that meet these criteria in each of the hospitals and have determined that this can easily be achieved; (2) we have an agreement with each hospital and their chief hospitalist to refer the patients to this study.**

d. Describe the inclusion/exclusion criteria: ***Inclusion*****: 65+, AA or W, has an illness that is either cancer, chronic progressive illness or frailty, the physician would not be surprised if they died within the year, and has family member/caregiver. *Exclusion*: Unable to complete baseline interviews, currently receiving hospice care, has no family member/caregiver, or does not meet the aforementioned inclusion criteria.**

e. If participants will comprise more than one group or stratification, describe each group (e.g., treatment/intervention, placebo, controls, sham treatment) **and** provide the number of participants anticipated in each group. **For this study we anticipate a total enrollment of 297 participants. Half of the patient participants will be randomized to receive usual care appropriate to their illness (including: assessment and treatment by the admitting physician, along with any subspecialists that are consulted).**

The other half will receive the culturally-based Tele-consult in addition to their usual care (including: standardized palliative care assessment, community-developed, culturally-based communication approaches, and follow up palliative care on day 3 and 6 post consult).

f. Indicate which, if any, of the special populations listed below will be involved in the protocol. Include the Special Populations Review Form (SPRF) if indicated.

☐ Pregnant Women: Attach SPRF—Pregnant Women, Fetuses, Neonates/Nonviable Neonates
☐ Fetuses: Attach SPRF—Pregnant Women, Fetuses, Neonates/Nonviable Neonates
☐ Neonates/Nonviable Neonates: SPRF—Pregnant Women, Fetuses, Neonates/Nonviable Neonates
☐ Prisoners: Attach SPRF—Prisoners
☐ Minors (<18 years old): Attach SPRF—Minors
☐ Employees or students at institution where research conducted
☐ Persons who are temporarily decisionally impaired
☐ Persons who are permanently decisionally impaired
☐ Non-English Speakers

For each box checked, describe why the group is included **and** the additional protections provided to protect the rights and welfare of these participants who are vulnerable to coercion: _____

g. List any persons other than those directly involved in the protocol who will be at risk. If none, enter "None": **None.**

h. Describe the recruitment process (e.g., medical record review, referrals, letter of invitation, existing patients) that will be used to seek potential participants (e.g., individuals, records, specimens). Research recruitment by non-treating physicians/staff may require completion of Partial Waiver of Authorization for Recruitment/Screening. **We will employ methods that were successful in our prior pilot studies and RCTs. Key to successful recruitment is engagement of the referring clinicians in the study from the outset. The hospitalists have been actively involved in the development of study procedures and are fully acquainted and in agreement with the palliative care physicians, study team, and study goals. Each site will also have two part-time study coordinators: a) Coordinating Study Coordinator (CSC) will review the electronic medical records at his/her respective site to screen, recruit, obtain consent, and organize the tele-consult. CSCs will remain in daily contact with hospital staff and participate in team meetings to remind clinicians about eligibility criteria. Team meeting attendance will allow the CSC to clarify the study goals and protocol and to**

strategize solutions if challenges arise. b) Blinded Study Coordinator (BSC) -The blinded study coordinator will collect all data following patient randomization.

CAG members will have three key roles:
1. Informing study team about Community Culture: CAG members will meet with and be trained by the southern rural AA and W Beaufort, SC CAG members who developed this protocol. We anticipate that the local CAG members will appreciate and recognize how their involvement will be instrumental in creating a program that could benefit their community.
2. Enlisting CAG Participation in study design: Local (AL and SC) CAG members will meet face-to-face with the study team initially and every three months to receive orientation and training in all proposed study procedures. Their input will be incorporated and the study team will regularly report on progress, and challenges, and will seek advice when problems arise. (No patient information will be discussed.)
3. Introducing Patients to the Study: In R21, patients and families (especially AAs) appreciated being greeted by CAG members who explained the study and the community's role in developing it. Therefore, for this study, CAG members will be the first to meet potential study (but will NOT consent the patient). NOTE: This step will depend on (a) whether all two hospitals will permit non-hospital staff to approach potential patients and (b) If all two hospitals do permit this, CAG members will have to go through specific training for volunteers.

i. If you will use recruitment materials (e.g., advertisements, flyers, letters) to reach potential participants, attach a copy of each item. If not, identify the source (e.g., IRB Protocol Number for approved databases) from which you will recruit participants. **There are no recruitment materials for this study. Only referrals from hospitalists.**

j. Describe the screening process/procedures for potential participants.

i. The CSC will screen admissions each weekday and will only approach potential study participants after confirming eligibility (i.e., clinician answer "no" to question: "would you be surprised if this person died in the next 12 months?" aka "surprise question") and getting approval from the treating clinician.

ii. CAG and CSC will meet with patient and family: Following approval, the CSC will contact the CAG member "on-call" for the day to speak with the patient.

iii. The CAG member and CSC will approach the patient. The CAG member will introduce themselves and the CSC, and describe the study to the patient. The CAG member will emphasize their role in developing the study and how this study was designed to help the community. If the patient indicates that he/she would like to participate, the CSC will provide an overview of the study, including a description of palliative care, the tele-consult equipment and process, (if randomized to that group), and randomization using a simple visual diagram.

iv. The CSC will then read the consent form to all patients (assuming no or limited literacy) unless the patient prefers to read it themselves. The family member will be invited to participate and will sign the consent form agreeing to complete questionnaires regardless of group assignment.

17. Protocol Procedures, Methods, and Duration - in nontechnical, lay language

a. Describe the procedures for all aspects of your protocol. Tell us what you are doing.

The study consists of 2 phases:

Phase I Convening & Soliciting CAG Participation in study design. This phase will consist of assembling CAG members to review study procedures and some CAG members will be trained to approach potential study participants. Potential CAG members (8, with equal numbers of AA and W) will be identified by the hospital staff and community leaders of each of the two sites, and invited to participate in the CAG. Potential CAG members will include respected members of the community, those who have recently lost a loved one and understand end of life care, and community leaders. Potential members will be discussed by the recruiters with the study PIs to ensure there is socio-demographic balance, and equal representation of African American and

White members, to ensure all will feel comfortable participating and contributing, and to ensure that they are not all from the same family/church/social group. Following this step, a formal written invitation will be extended to those selected, followed by a phone call by the Study Manager.

Phase II Conducting the RCT. In Phase II we will conduct a randomized controlled trial (RCT) at the 2 sites to test the effectiveness of this innovative program. We will recruit 297 hospitalized AA and W adults over age 65 whose treating physician would not be surprised if they died within the next year and the patients' family/friend caregiver. Half of the patient participants will be randomized to receive usual care appropriate to their illness. Patients randomized to usual care in each of the two participating hospitals will receive inpatient care appropriate to their illness. This includes assessment and treatment by the admitting physician, along with any subspecialists that are consulted. The formulation and sharing of prognostic information and the engagement of patients and families in establishing goals of care will be according to the standards of the admitting physician and subspecialists. Treating clinicians may include hospitalists, nurse practitioners, social workers, pastoral care, and occasionally, the patient's primary care practitioners who have hospital privileges.

The other half of the patient participants will receive the culturally-based palliative care consult. Patients in the intervention group will receive usual care as described above, and, in addition, will participate in a standardized, comprehensive palliative care Tele-consult assessment (based both on National Consensus Project [NCP] guidelines and on Culturally-based interactions community in the R21) as well as a follow up by the palliative care physician on Day 3 and 6 following the initial consult.

- Contact #1: the Coordinated Study Coordinator [CSC] will work closely with the patient/family, local team, and remote palliative care physician to determine a time suitable (within 48 hours on weekdays) to perform the Tele-consult. The CSC and the hospitalist/a member of the treatment team will be in the room with the patient and family. The CSC will set up the HIPAA protected telehealth laptop next to the patient's bed, introduce the patient to the remote palliative physician, and the tele-consult will proceed. All Tele-Consults are digitally-recorded within the secure portal. Most consults take 45-60 minutes. To ensure a comfortable conversation with minimal interruptions we have instituted several steps including checking with floor nurse, putting up signs on the door, and tasking the CSC with running interference in case of interruptions.
  - Within 24 hours of conducting the Tele-consult, the remote palliative physician will seek an interdisciplinary team review, develop the plan, and make recommendations, which may include immediate patient care (e.g., symptom relief) and continuing care, related to the hospital stay and following discharge. Recommendations may also include referrals to other supportive resources such as a hospital social worker or pastor. The written consult will be documented using a standardized note template that corresponds to the 8 NCP palliative care domains and will be provided via a secure HIPAA-compliant portal that will be part of the patients' inpatient medical record. As needed, verbal contact will also occur between the palliative care physician and the hospitalist staff.
- Contact #2 Day 3 (approximately 24-48 hours after first contact). The CSC will arrange a follow up videoconference between the remote palliative care physician and patient if in hospital or via phone if the patient has been discharged. The purpose of this second contact is to determine how patient is progressing, if the plan was implemented, if hospital staff/patient has encountered any challenges or questions, and to determine who will be

assuming the patient's care in the community. Documentation of the contact will be provided as described above.

- **Contact #3 Day 6 (post consult) (via videoconference if patient is in hospital, via phone if discharged.) To determine if patient is experiencing new or ongoing challenges, to confirm availability of community or hospice care as appropriate.**

In the usual care group and the intervention group, a study coordinator at each site will administer questionnaires to the patient and their designated family/friend caregiver, in person or by telephone (if patients are discharged during the study period) at baseline (T1), Day 7 (T2) and Day 30 after hospital discharge (T3). (Study coordinators administering T2/T3 assessments will be blinded to study group- aka Blinded Study Coordinators [BSC]).

UAB has Cisco based infrastructure for teleconferencing. The primary teleconferencing equipment to be used by providers at UAB will be Cisco Jabber with a high definition webcam as well as a Cisco SX10. This infrastructure will utilize a Cisco based call manager located at UAB for videoconferencing. This infrastructure will allow for a flexible set up to allow for videoconferencing to a mobile device or full telemedicine cart and is fully HIPAA compliant. UAB also has a process to privilege providers external to UAB. Once privileged at UAB external providers will have access to UAB videoconferencing capabilities.

Telehealth infrastructure in South Carolina can be used by multiple institutions. Medical University of South Carolina (MUSC) deploys telehealth technologies, which are capable of being accessed by standards-based video applications. While multiple video clients and other technologies are in use throughout South Carolina, MUSC utilizes a largely Cisco based infrastructure which is compatible with the UAB technologies. In addition, MUSC provides gatekeeper capabilities to link other standards-based video clients. Lastly, the federally subsidized Palmetto State Provider Network provides many health care facilities in South Carolina with broadband and infrastructure, which is redundant to MUSC by design, allowing for a truly open-access network for the state and beyond.

b. What is the probable length of time required for the entire protocol (i.e., recruitment through data analysis to study closure)? **5 years (see timeline of Study Activities below)**

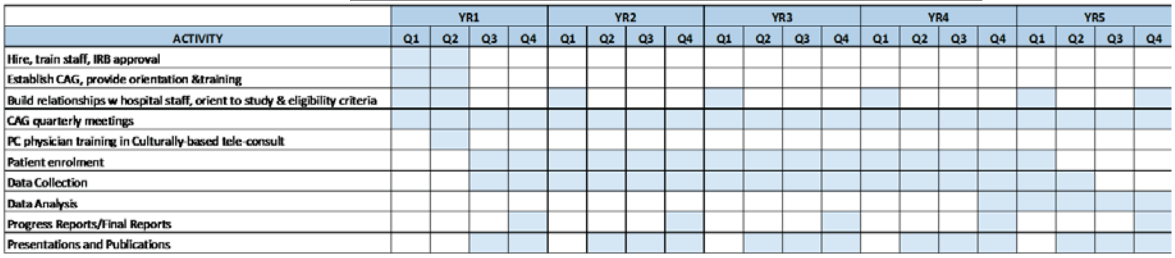

c. What is the total amount of time each participant will be involved?
Usual Care: 30 days after initial enrollment.
Tele-consult intervention: 30 days after initial enrollment

d. If different phases are involved, what is the duration of each phase in which the participants will be involved? If no phases are involved, enter "None." **In the first phase of the study, Community Advisory Groups (CAGs) will be involved (there will be no study patients involved during this phase). In the second phase of the study, patients will be involved 30 days after initial enrollment.**

e. List the procedures, the length of time the procedure takes, the total # of times the procedure is performed, and indicate whether each is performed solely for research or would already be performed for treatment or diagnostic purposes (routine care) for the population.
-Insert additional table rows as needed.

-If procedure is sometimes research and sometimes routine care, include on separate lines with number of times as each.

Procedure	Length of Time Required of Participants	Total # of Times the Procedure is Performed	Research (Res) –OR- Routine Care
**Patient Demographics**<br><br>***(Usual care [control] and palliative care tele-consult [intervention])***	**Demographic questionnaire- age, gender, race/ethnicity, marital status, religion, education, occupation, insurance status, financial status. This should take approximately 25 minutes**	**Once**	☒Res ☐Routine
**Family Member (caregiver) Demographics**<br><br>***(Usual care [control] and palliative care tele-consult [intervention])***	**Demographic questionnaire- age, gender, race/ethnicity, marital status, religion, education, occupation, insurance status, financial status. This should take approximately 25 minutes**	**Once**	☒Res ☐Routine
**Patient Baseline and Day 7 Post Baseline Survey(s)**<br><br>***(Usual care [control] and palliative care tele-consult [intervention])***	**At baseline a study coordinator will ask the patient questions related to symptom burden (Edmonton Symptom Assessment Scale [ESAS]), patient Quality of Life (Patient-Reported Outcomes Measurement Information System [PROMIS Global Health-10], patient satisfaction with care (Feeling Heard and Understood questionnaire). Each survey should take approximately 30 minutes). Collectively, the questionnaires should take no approximately an hour and 30 minutes**	**Twice (baseline and Day 7)**	☒Res ☐Routine
**Caregiver Baseline and Day 7 Post Baseline Survey(s)**<br><br>***(Usual care [control] and palliative care tele-consult [intervention])***	**At baseline a study coordinator will ask the patient questions related to caregiver Quality of Life (PROMIS Global Health-10), family satisfaction with care (FamCare), and caregiver burden (Montgomery Borgatta Caregiver Burden Scale [MBCB]). Each survey should take approximately 30 minutes). Collectively, the questionnaires should take no approximately an hour and 30 minutes**	**Twice (baseline and Day 7)**	☒Res ☐Routine
**Resource Use (Day 30 post baseline)**	**Number of hospital readmissions, number of hospital days, number of ICU days, number of emergency department (ED) visits, and hospice days during the 30 days following discharge will be collected via electronic health record and patient/family report.**	**Once (Day 30 post baseline)**	☒Res ☐Routine

	**Patient/Family report should take no longer than 25 minutes**		
**Palliative Care Tele-consult (intervention)**	**As soon after randomization, patients will participate in the first tele-consult (approximately 1 hour). At Day 3, the patient will participate in a follow up consult (approximately 45-60 minutes). At day 6, the patient will participate in a second follow up consultation (approximately 45-60 minutes)**	**Patients in the intervention group will participate in 3 tele-consult sessions.**	☒Res ☐Routine

f. Will an interview script or questionnaire be used? ☒Yes ☐No

If Yes, attach a copy. **See Survey or questionnaire. All instruments were chosen to measure distinct constructs within the conceptual model. These instruments have been used in the pilot R21 and our previous studies and presented minimal burden to patient and family (Table 5).**

g. Will participants incur any costs as a result of their participation? ☐Yes ☒No

Table 5. Measures and Data Collection Schedule					
Construct	Instrument & Description	# of Items	T1 Baseline	T2 Day 7	T3 Day 30
Demographics	Demographic Questionnaire- Age, gender, race/ethnicity, marital status, religion, education, occupation, insurance status, financial status.	10	X		
Primary Aim					
Symptom Burden	**Edmonton Symptom Assessment Scale (ESAS)** - symptom intensity using visual analog	9	X	X	
Secondary Aims					
Patient and Caregiver QoL	**PROMIS Global Health -10**. Evaluates physical, social, and emotional health in healthy & chronically ill adults	10	X	X	
Family Satisfaction with Care	**FamCare** - family satisfaction with availability of care, physical & psychosocial care, information giving	20	X	X	
Patient Satisfaction with Care	**Feeling Heard and Understood** - Self-report quality measures for palliative care settings on Likert scale (1 item)	1	X	X	
Caregiver Burden	**Montgomery Borgatta Caregiver Burden Scale (MBCB)**-subscales objective, subjective, demand burden	14	X	X	
Resource Use	**Hospital admission, ED visit, hospice**	NA			X
Exploratory Aim					
Mediators	Consult recommendations implemented by Hospitalist & Pt	NA		X	

If Yes, describe the reason for and amount of each foreseeable cost. _____

h. Will participants be compensated? ☒Yes ☐No

If Yes, complete i-v.

Patient Payment:

i. Type: (e.g., cash, check, gift card, merchandise): **check**

ii. Amount or Value: **$40**

iii. Method (e.g., mail, at visit): **Delivered in person or mailed**

iv. Timing of Payments: (e.g., every visit, each month): **Following completion of questionnaires at Day 7 (T2).**

v. Maximum Amount of Compensation per Participant: **$40 check**

18. Benefits

Describe the potential benefits of the research. **The studies all take place in Health Professional Resource Shortage areas in the South. There are no palliative care physicians or staff in these areas who could provide palliative care services. Palliative care has been widely demonstrated to provide benefits to patients in terms of reduction of pain and other symptoms and enhancement of quality of life to patient and caregiver. Participants in the intervention group may benefit by talking to a palliative care doctor and receiving care that is not readily available in the hospital. The possibility of relief from pain and other symptoms may improve patient and caregiver quality of life. Participants may also experience benefit from talking with a palliative care physician who has been trained by the very same community members who participated in developing the program in how to understand talk to, and treat AA and W people in the rural South.**

19. Risks - in nontechnical, lay language

a. List the known risks for participants as a result of participation in the research. This should not include the minimal risk of loss of confidentiality. However, it should include any physical, psychological, social, economic, and/or legal risks. If there is a greater than minimal risk of loss of confidentiality describe why this is so. Do not list risks associated with the standard-of-care procedures.
NOTE: Risks included here should be included in the consent form or information sheet, as applicable.
This study has very low risk to participants. Risks that they potentially incur include:
i. Emotional distress: Talking about their illness and symptoms may cause emotional distress for patients or caregivers. Patient may get tired while answering the questions with the study coordinator. If a patient gets too tired, we can come back later in the day. Sometimes people feel embarrassed or uncomfortable when being asked questions so patients/caregivers can refuse to answer any question.
ii. Loss of confidentially: There is a chance that people not associated with the study will see patient/caregiver answers to questionnaires. Patient/caregiver name and other identifying information will be removed from study documents. Data will be kept in locked files in the study research offices at UAB. All data will be housed in a secure, password protected database at UAB.
iii. Burden and benefits: There is a risk related to being placed into a group by chance (i.e., randomization). Patients in the standard care group may not have the same benefits as patients in the intervention group.

b. Estimate the frequency, severity, and reversibility of each risk listed. **Very low risk**

c. Is this a therapeutic study or intervention? ☒Yes ☐No
If Yes, complete i.-iii.
i. Describe the standard of care in the setting where the research will be conducted: **Patients in the standard care group will receive inpatient care appropriate to their illness. This includes assessment and treatment by the admitting physician, along with any subspecialists that are consulted. The formulation and sharing of prognostic information and the engagement of patients and families in establishing goals of care will be according to the standards of the admitting physician and subspecialists.**
ii. Describe any other alternative treatments or interventions: **The patients in the therapeutic intervention group is IN ADDITION to their standard medical care. Recommendations made by the PC physician will be communicated to the hospitalist who will be the final determiner of patient treatment.**
iii. Describe any withholding of, delay in, or washout period for standard of care or alternative treatment that participants may be currently using: **There is no delay/washout period/withholding.**

d. Do you foresee that participants might need additional medical or psychological resources as a result of the research procedures/interventions? ☐Yes ☒No
If Yes, describe the provisions that have been made to make these resources available. _____

e. Do the benefits or knowledge to be gained outweigh the risks to participants?

☒Yes ☐No

If No, provide justification for performing the research: ______

20. Precautions/Minimization of Risks

a. Describe precautions that will be taken to avoid risks and the means for monitoring to detect risks.

Protection against Risk:

i. Distress to patient and/or caregiver about the potential life-limiting prognosis. Not all patients and/or family members will be aware of this. No reference will be made at any stage, in writing or verbally, to the patient and/or family member about prognosis or endo of life diagnosis. Reference will only be made (e.g., in terms of why this patient was selected for participation in the study) to the fact that the patient has an illness and is in hospital.

ii. Protection of data: We make many efforts to protect the identities of the participants and the confidentiality of the research data used in this study. This includes:

• The only research document with patient name on it will be the consent form. It will be stored in a locked file cabinet stored separately from our other study documents.

• For all other study documents, patients will be given a study identification number (not linked to any of the patient's personal numbers like phone number) that will be used instead of patient name. This means that no one, other than the statistician for the study and the study coordinator, can identify whom the data belongs.

• The only link between patient name and study data will be one document (called linking log) with patient name and study identification number. The linking log will be kept on a computer that is password protected, and in a locked office. Only the statistician will have access to this file. Once we complete analyzing the study results, this linking log will be destroyed.

If the protocol involves drugs or devices skip Items 20.b. and 20.c. and go to Item 21. Instead include this information in the Drug Review Sheet or Device Review Sheet, as applicable.

b. If hazards occur to an individual participant, describe (i) the criteria that will be used to decide whether that participant should be removed from the protocol; (ii) the procedure for removing such participants when necessary to protect their rights and welfare; and (iii) any special procedures, precautions, or follow-up that will be used to ensure the safety of other currently enrolled participants. **N/A, this study has minimal risk. Therefore, we anticipate no hazards**

c. If hazards occur that might make the risks of participation outweigh the benefits for all participants, describe (i) the criteria that will be used to stop or end the entire protocol and (ii) any special procedures, precautions, or follow-up that will be used to ensure the safety of currently enrolled participants. **N/A, this study has minimal risk. If over the course of the study hazards are identified the PIs will consult with co-investigators and the IRB in making any such decisions**

21. Informed Consent

a. Do you plan to obtain informed consent for this protocol? ☒Yes ☐No

If Yes, complete the items below.

If No, complete and include the Waiver of Informed Consent or Waiver of Authorization and Informed Consent, as applicable.

b. Do you plan to document informed consent (obtain signatures) for this protocol? ☒Yes ☐No

If Yes, complete the items below.

If No, complete the items below **and** include the Waiver of Informed Consent Documentation.

c. How will consent be obtained? **In person**

d. Who will conduct the consent interview? **Study coordinator (Study coordinators have not yet been identified, and will be added via PRAF when available).**

e. Who are the persons who will provide consent, permission, and/or assent? **Patient and caregiver**

f. What steps will be taken to minimize the possibility of coercion or undue influence?

i. Training will be provided to the study staff explaining the importance of patient/caregiver decisions to participate or not.

ii. Refusal to participate will not affect the patient's treatment in any way. This is also explained clearly in the consent form.

iii. Community members will explain how the intervention was developed and they will be trained in understanding that it is completely up to the patient and caregiver if they wish to participate or not.

g. What language will the prospective participant and the legally authorized representative understand? **English**

h. What language will be used to obtain consent? **English**

i. If any potential participants will be, or will have been, in a stressful, painful, or drugged condition before or during the consent process, describe the precautions proposed to overcome the effect of the condition on the consent process. If not, enter "None." **Only patients whose physicians give permission to approach will be included in the study.**

j. If any protocol-specific instruments will be used in the consenting process, such as supplemental handouts, videos, or websites, describe these here and provide a copy of each. If not, enter "None." **None**

k. How long will participants have between the time they are told about the protocol and the time they must decide whether to enroll? If not 24 hours or more, describe the proposed time interval and why the 24-hour minimum is neither feasible nor practical. **A 24 hour "think it over period" is feasible and practical for the study.**

22. Procedures to Protect Privacy

Describe how you will protect the privacy interest of the participants. Include how you will make sure others cannot overhear your conversation with potential participants and that individuals will not be publicly identified or embarrassed. **All consent and questionnaires will be completed in the privacy of the patient's room. If the patient is sharing a room, and can be moved, we will interview the patient/caregiver in a closed room. The PC consult will take place in a private office used specifically for this purpose. If the patient is too ill to be moved, the PC consult will take place at the bedside. Room door and/or patient curtain will be closed.**

23. Procedures to Maintain Confidentiality

a. Describe how you will store research data to maintain confidentiality (both paper records and electronic data), including how access is limited. If data will be stored electronically anywhere other than a server maintained centrally by UAB, identify the department and all computer systems used to store protocol-related data. **The Blinded Site Coordinators will be trained to collect patient and caregiver reported data in the hospital and by phone following discharge. It will be directly entered into the REDCap database that will be housed at UAB. REDCap is a secure web application for building and managing online surveys and databases. The statistician will build a suitable database with facilities for data collection and storage. All databases are secure, HIPAA compliant and password protected in both the front end and back end. Back end data will be housed on a secure drive with access limited to authorized research personnel only. REDCap supports a HIPAA best practice, and is a secure web-based application enabling multiple sites to seamlessly access, analyze and share data while maintaining the security and integrity of the database and provides automated export procedures for seamless data downloads to common statistical packages.**

b. Will any data from this protocol be given to any person, including the subject, or any group, including coordinating centers and sponsors? ☒Yes ☐No

If Yes, complete i-iii.

i. Who will receive the data? **Community Advisory Group**

ii. What data will be shared? **Study progress and final results of the study**
iii. How will the data be identified, coded, etc.? **ONLY grouped data will be provided. NO individual data will be shared. No identifiers will be included.**

24. Genomic Data Sharing (GDS)
Researchers who collect genomic data as part of a NIH grant funded after January 25, 2008 may be required to submit those data to a NIH database for broad scientific sharing. See Genomic Data Sharing in the IRB Guidebook for more information.

a. Does this protocol involve the proposed submission of genetic data into genomic repositories created to share genetic information for research purposes? ☐Yes ☒No

b. Will UAB be uploading the final genomic data to the central repository (e.g., dbGaP)? ☐Yes ☒No
If Yes to both a and b, submit a Detailed Data Sharing Plan to the IRB for review. This plan should include any known data use limitations and indicate whether aggregate-level data are appropriate for general research use. For guidance see the NIH Genomic Data Sharing Policy.

c. Submit a copy of the NIH Institutional Certification Form.
To determine which certification form to include, answer i-ii.
i. Was this protocol funded prior to January 25, 2015? ☐Yes ☐No
- **If yes,** and consent will be obtained, submit the Extramural Institutional Certification - Before January 25 - With Consent.
- **If yes,** and consent will not be obtained, submit the Extramural Institutional Certification - Before January 25 - Without Consent.

ii. Was this protocol funded after January 25, 2015? ☐Yes ☐No
- **If yes,** submit the Extramural Institutional Certification - After January 25.

25. Additional Information
In the space below, provide any additional information that you believe may help the IRB review the proposed research, or enter “None.” **None**

A. SIGNIFICANCE

A1. Palliative care consultations for inpatients have successfully identified unrecognized symptoms and unmet needs,[41-45] have been associated with lower ICU use,[22,24] fewer ICU deaths,[46] improved care processes, and higher rates of goals of care documentation.[47-49] The triple threat of rural geography, racial inequities, and older age hinders access to high quality palliative care for a significant proportion of Americans. In a state-by-state report card,[50] the Southeastern US, where a significant proportion of the population is rural and African American (AA), palliative care access was ranked the lowest in the nation. Rural patients with life-limiting illness are at very high risk of not receiving appropriate care due to a lack of health professionals (nearly two-thirds of rural US counties are designated health professional shortage areas),[2] long distances to treatment centers,[3] and limited palliative care clinical expertise.[4] Even when palliative and hospice services are available, AA, compared to W are more likely to receive medically-ineffective, poor quality and high cost care, due to general mistrust of health care providers and a fragmented health care system that is generally insensitive to cultural differences that can guide treatment choices.[51-56]

A2. AA and Ws end-of-life (EOL) cultural values differ. Despite proven effectiveness, numerous studies have shown that AAs underutilize palliative and hospice care.[48,49,57-60] Three reasons have been suggested:

1) A lack of exposure to hospice or palliative care information,[61-63]

2) AAs' values of EOL care differ. Historically EOL care has been rooted in W middle class cultural and religious values;[1,62] a very different frame of reference, value system, and life experience compared with many AA.[64] Where middle class W may emphasize individual choice; AA values support family-centered decision-making.[65] AAs' often value faith, spiritual beliefs and guidance of a spiritual leader,[62] especially as they cope with illness and make treatment decisions.[66,67] Yet physicians rarely ask patients about their spirituality.[68] AA's reliance on hope[69] and faith in God's healing power,[70] can be at odds with physicians' felt need to share a terminal prognosis.[57]

> *"I think a lot of times it's about us, and we want them there at all cost, and love has a lot to do with it because we don't want that parting, but I also say death and dying has a lot to do with our faith. And if we believe whatever the word say, if we believe that – I'm not saying that it's easy, but it has to do with – it has to do with our faith and our culture and our community at large. And just like J. was saying, you can remember, we went to the church for everything. Church was the leader of everything"*
> -AA focus group participant explaining how cultural beliefs influence desire for end-of-life care.

3) Historical and social factors including slavery, racism, medical experimentation and exploitation,[57,71] and ongoing racism and microaggression,[72] have left a deep-seated legacy of mistrust in the AA community.[73-75] A recent report [76] found that, AAs and Ws are "worlds apart" in their perceptions of racial equality and gaps in household income. This is even more strongly felt in the "Deep South" where slavery was promoted.[77] A recent study[78] found AAs are more likely than other racial groups to believe physicians don't care about them as individuals, and are less likely to trust their physicians' judgment and personal competence.

A3. A community-developed, culturally-based intervention can promote palliative care acceptability. Culture fundamentally shapes how individuals make meaning out of illness, suffering, and dying,[79] and strongly influences their responses to diagnosis, illness, and treatment preferences.[79-81] Consideration of the patient and their family's culture is essential in palliative care; lack of sensitivity to; lack of respect for cultural differences may compromise EOL care for minority patients.[1] Yet culturally appropriate models of care that take into consideration the diverse cultural preferences of seriously-ill rural patients and their family caregivers are not currently available in the US. There is an urgent need for research that emphasizes varying EOL care cultural preferences.[1,6,7,65,82-84] Community-based participatory research (CBPR), a public health method in which academia and the community form a joint partnership to address community issues, has been found to address health disparities[85,86] and result in demonstrable positive health outcomes.[87] CBPR builds on community strengths. The community is integral to all phases of the research, for the mutual benefit of all partners involved in the process, and in disseminating findings and knowledge to all partners.[88] Consumer input has been demonstrated to enhance both the quality and acceptability of interventions.[89] We believe that this proposal is the first to *employ this methodology to develop and test the efficacy of a culturally-based palliative care tele-consult program for hospitalized AA and W rural elders and their families.*

A4. A Tele-consult program for hospitalized AA and W can reduce symptom burden by improving access to high quality palliative care in rural, underserved areas. Over the last 20 years, tele-health, the remote delivery of health care and sharing of medical knowledge using telecommunication has been used to deliver health care to remote areas for a variety of illnesses.[90-96] Our teams were among the first to conduct large trials of palliative

care by tele-health that reduced symptom and caregiver burden and improved quality of life of seriously-ill patients in rural northern New England[22,24,26,27] and in the Deep South states of Alabama, Mississippi, Georgia, South Carolina.[9,10,13,20,97-99] There have been a few successful, locally-based initiatives to expand palliative expertise to rural areas through community partnerships,[100,101] our team has conducted the majority of these community-based-partnership initiatives to create this evidence-base in the US.

> *"He was of his right mind and he went out just like he wanted. And I mean, I can't ask for more than that that there was no more pain, no suffering or anything, except for, you know, I did the suffering, but anyway, he got what he wanted."*
>
> -Family Caregiver of an AA patient who was able to receive palliative care

B. INNOVATION

This innovative proposal will move the field forward in three ways:

(1) The first study to investigate the efficacy of a culturally-based palliative care Tele-consult program for inpatients that was developed using **CBPR** methods-that is it was developed *by* and *for* the population it will serve. Community Advisory Groups (CAGs) were involved over 24 months to guide initial development of the intervention. The original CAG will mentor local sites' CAGs in Phase I of our proposal. Once again the new local CAGs will consist of AA and W community members who represent the intervention end-users. Importantly, a CAG member will introduce the study to the patient prior to the study coordinator obtaining consent. If effective this model can be used to develop other culturally-based palliative care programs.

(2) We will apply tele-consult methods that have been used successfully in other health conditions, to improve palliative care accessibility in rural areas; however, the Tele-consult program can also be applied in any environment that limits seriously-ill patients' ability to receive expert palliative care. Our teams were the first to demonstrate the effectiveness of tele-health in improving rural patients and family caregivers' palliative care access and outcomes in areas with serious palliative care shortages. [22,24,26,27,98]

> *"But the bad thing is here, we're limited in this small town to where we can go. Both hospitals… you gotta go to one hospital or you gotta drive a long ways."*
>
> -Focus Group Participant

(3) This study focuses on the understudied, underserved southern rural population, with capacity for translation and dissemination to similar palliative care shortage areas and to other cultural groups.

C. APPROACH

C1 Overview of Design (see Figure 1)

Research question: This RCT was designed to answer the question: "Is a community-developed, culturally-based palliative care Tele-consult program efficacious in reducing hospitalized AA and W patients' symptom burden (primary aim) and improving patient and family caregiver quality of life, caregiver burden, and reducing resource utilization (secondary aims)? Our exploratory aim will be to explore potential mediators and moderators of Tele-consult effects.

FIG. I. STUDY DESIGN

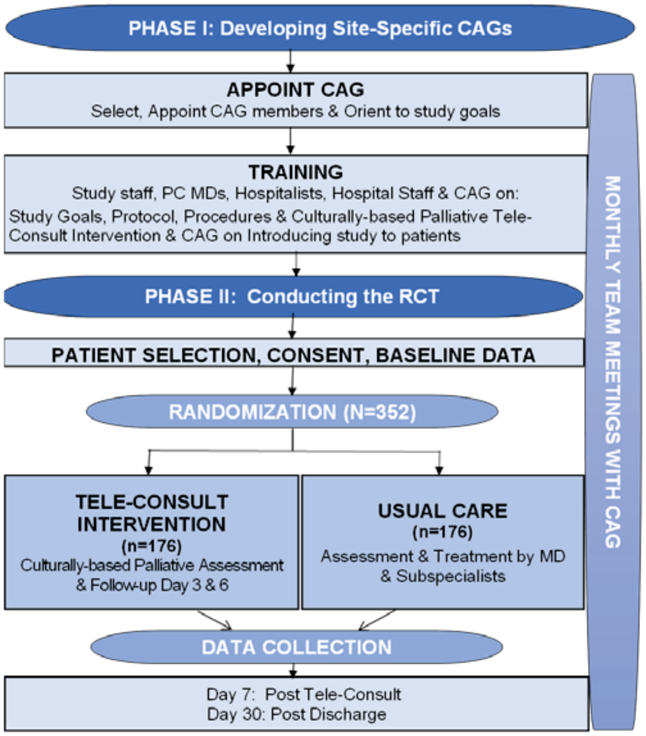

Design overview: The study consists of 2 phases (Figure 1). Phase I Convening & Soliciting CAG Participation in study design. This phase will consist of assembling AA and W community members at each study hospital (one each in South Carolina, Alabama, and Mississippi) to review study procedures and some will be trained to approach potential study participants. Phase II Conducting the RCT. In Phase II we will recruit 352 hospitalized AA and W adults over age 65 whose treating physician would not be surprised if they died within the next year and the patients' family/friend caregiver. Half of the participants at each hospital will be randomized to receive the Tele-consult program (initial consult and 2 follow up contacts). The Tele-consult program is designed to bring interdisciplinary palliative care expertise to patients receiving care in

hospitals that lack palliative care expertise. The other half of participants will be assigned to receive the usual care that is provided to hospitalized inpatients (e.g. hospitalists, social workers, discharge planners and other support services).

Assessments: A study coordinator at each site will administer assessments to the patient and their designated family/friend caregiver, in person or by telephone (if patients are discharged during the study period) at baseline (T1), Day 7 (T2) and Day 30 after hospital discharge (T3). (Study coordinators administering T2/T3 assessments will be blinded to study group). The time frame between T2 and T3 (Day 30) may vary depending on the length of time after the initial Tele-consult until discharge. The T2 assessment will capture the short term (main) effect of the Tele-consult intervention on the primary patient symptom outcome. The T3 assessment measures any sustained patient and caregiver effects and Tele-consult impact on resource use, primarily 30-day hospital readmission. Our decisions regarding the timing of these assessments are based on a recent study that found short term quality of life and symptom improvements on hospitalized hematology patients.[102] The T3 measures the standard 30-day hospital readmissions quality indicator.

Rigor and transparency. In accordance with NOT-OD-16-012, we discuss the following issues to maximize the rigor of the design: randomization (C6), blinding of data collectors (C5.1), transparent eligibility criteria (C5.1), treatment protocol fidelity monitoring (C7.2.2), and adjustment for multiple inferences (D.).

C2. Preliminary Studies Supporting the Scientific Premise

Overview. Our team has over a decade of experience developing culturally-based, patient-centered palliative care telehealth interventions, including the pilot study (1R21AG046772-01) that underlies the Tele-consult program. Our prior work has addressed issues of feasibility, acceptability, study design, measures, procedures, and efficacy making us confident that the proposed RCT will be successful.

C2.1 Engaging a CAG and using CBPR for intervention development. We used CBPR principles to engage a Community Advisory Group (CAG) over two phases. Phase 1: (a) We appointed a CAG comprised of 14 community members and leaders, equal numbers of AA and W, who had cared for and lost a loved one in the preceding year, and two members of hospital staff (senior nurse and hospital community liaison). (b) Based on AA CAG member input, we conducted separate AA and W focus groups to understand family members' preferences for EOL care and communication. Common and differing themes are illustrated in Tables 1-3.

C2.1.1 Developing the Culturally-Based Palliative Care Tele-Consult. In Phase 2, as shown in Figure 2, a second CAG, comprised of original CAG and new focus group members used a participatory process and met monthly for twenty-four months to develop the culturally-based Tele-consult guiding communication principles. Additionally, they strongly recommended that **a community member** who had played a role in program development, rather than a study coordinator be the patients' first contact to help bridge the distrust gap.

FIG. 2: PHASE 2 DEVELOPING THE CULTURALLY-BASED PC TELE-CONSULT PROGRAM

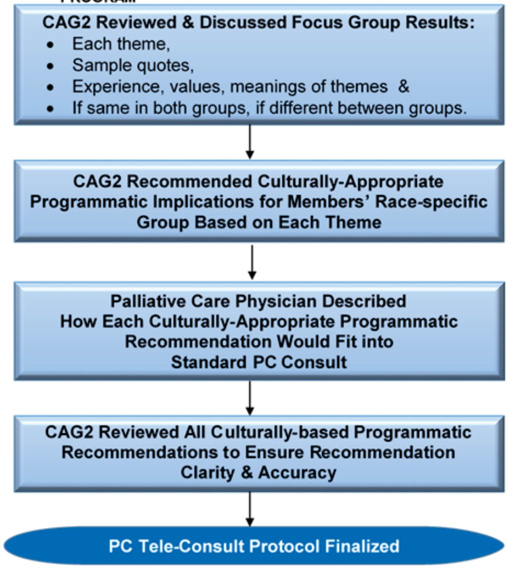

C2.1.2 Technology acceptability/feasibility: CAG members: Several focus group members in both racial groups initially felt uncomfortable speaking to a physician on a computer. However following a full demonstration, CAG members' comfort level increased. They made 3 recommendations enhance potential study patients' comfort with the technology: 1) CAG members who first meet the patient explain and de-mystify the Tele-Consult process. 2) CAG member share a visual schema, which they developed, with the patient; and 3) The palliative

physician begins the Tele-Consult by acknowledging the potential awkwardness of it, but that the physician

TABLE 1: CULTURALLY-BASED COMMUNICATIONS WHEN MEETING W/ AA & W RURAL SOUTHERN ELDERS & FAMILIES				
A. COMMUNICATION DURING CONSULT	RACE	UNDERLYING EXPERIENCES, CULTURAL VALUES, PERSPECTIVES		PROGRAMMATIC IMPLICATIONS RECOMMENDED BY COMMUNITY ADVISORY GROUP (CAG)
		AFRICAN AMERICAN	WHITE	
Communication Throughout PC Consult	W & AA	Experiences of rude behavior by a MD towards PT/family contributed to not wishing to talk with physician.		PC MD should always be courteous, never rude.
				PC MD should always respect patient/family confidentiality.
				If PT/family not responding, PC MD should ask if would like to reschedule.
Building Trust & Establishing Rapport	W & AA	Experiences of MDs showing lack of respect towards PT/family and not respecting confidentiality contributed to distrust in MDs.		PC MD must make an effort to get to know patient & family.
				Take time getting to know patient & family.
				During conversation, repeat back what you learned about PT/family.
				During conversation, also talk about something local.
	AA	Long history of distrust of MDs and hospitals based on history of medical mistreatment.	N/A	Steps towards reducing distrust:<br>(1) Meeting AA CAG;<br>(2) CAG member explaining how program was developed by community members who can vouch for it;<br>(3) CAG member introduces patient & family to Study Coordinator (SC).
Explaining Medical Information	W & AA	Experiences of MDs not explaining complicated terms or medications contributed to lack of understanding and frustration.		(1) PC MD should provide information in an easy to understand non-"medical" language.
				Explain slowly, clearly, one step at a time.
				Offer opportunity to ask questions.
				If PT/family don't understand, explain it differently. Take onus of responsibilty for clarity on self.

TABLE 2: CULTURALLY-BASED COMMUNICATIONS ABOUT PROGNOSIS, ADVANCED DIRECTIVE (AD) & RELIGION WHEN MEETING WITH AA & WHITE RURAL SOUTHERN ELDERS & FAMILIES				
2. DISCUSSING PROGNOSIS, ACP & RELIGION	RACE	UNDERLYING EXPERIENCES, CULTURAL VALUES & PERSPECTIVES		PROGRAMMATIC IMPLICATIONS RECOMMENDED BY CAG
		AFRICAN AMERICAN	WHITE	
Discussing Prognosis	AA	(1) Only God knows when person will die.<br>(2) Hope is key concept in African American church.<br>(3) As long as patient is alive, always possibility of a miracle.	N/A	*Never* tell PT/family prognosis, or ask if wants to know prognosis.
				If PT/family asks you about prognosis: Follow all 5 steps below:<br>(1) Never be blunt; (2) Never tell PT they are dying; (3) Never put time and date on prognosis (always state as estimated range); (4) Explain (in very simple terms) what is happening in body; (5) Stress that you're only the doctor. When the patient will die is in God's hands. (If PC MD, not comfortable saying, "in God's hands", say "in the hands of a HIgher Power.")
		Deep appreciation of MDs who bring spirituality & God into conversations on death and illness	N/A	(1) If PT/family are religious, PC MD can say, "I see that you are a spiritual person, we are doing the best we can and it's in God's hands."<br>(2) If PC MD feels comfortable, ask if you can pray with PT/family.
	W	NA	Discussing or not discussing prognosis depends upon PT/ family wishes.	(1) Determine with delicate sensitivity if PT/family want to know and if so, what they want to know; (2) Follow the PT/family lead & respond to their wishes; (3) Never be blunt; (4) Offer PT/family opportunity to provide input; (4) Offer PT/family opportunity to provide input; (5) Explain that though there may not be options to treat disease, there are options about where the PT can be -- home or elsewhere; (6) Allow PT/family to make own decisions.
Discussing Advance Care Directive	AA	(1) No need to have it in writing;<br>(2) We know in our hearts what is right;<br>(3) She/he told us what she/he wanted and that's what we'll do.	N/A	(1) If AD not in chart (highly likely), ask PT/family what their wishes are.<br>(2) If PT unable to communicate, ask if they had communicated their wishes to family, what they were & how you can help make sure they're followed.<br>(3) Confirm that PT/family don't want the AD in chart. (Remember: Distrust in written plan very high)
Discussing Religion	AA & W	Religion is a comfort for both patients and caregivers.		Ask about church membership. "Can you tell me about your home church?"
				If PT/family tell you about their church, acknowledge the importance of their religion as a comfort.
	AA	(1) The church is the center of our life and the Pastor is center of the church.<br>(2) When a patient is transitioning, there will always be a Pastor there, we let the Pastors know. We don't let anyone transition alone.	NA	(1) Ask patient/family about their pastor (name, church).<br>(2) Determine if they have had recent contact with pastor.<br>(3) If PT is about to transition, don't ask if wants you to contact pastor (pastor has been contacted).
	W	NA	Church members provide support.	(1) Ask about church membership. "Can you tell me about your home church?"<br>(2) Determine if they have spoken to their pastor, church members.<br>(3) If they haven't, ask if it would be helpful for you to contact someone from the church for them.

reassures the patient/family that once they get acquainted, most patients feel like they are in the same room.

<u>C2.1.3 Technology acceptability/feasibility: Hospital staff & hospitalists</u>: The Study Coordinator and the remote palliative physician met regularly and participated in meetings with hospitalists, nurses, and care coordinators to explain study goals and share eligibility criteria and participate in their meetings. All but one of 14 hospitalists were supportive of the study. We used these meetings to identify important protocol changes that led to successful implementation. Physician-physician contact nearly doubled the patient referrals (e.g. from 8 referrals in 15 weeks to 11 referrals in 7 weeks), and improved physician communication. Because of unanticipated delay in the palliative physician receiving his South Carolina license, we were only able to recruit patients for 4 months.

TABLE 3: CULTURALLY-BASED COMMUNICATIONS ABOUT NURSING HOMES, HOSPICE & OTHER SERVICES WHEN MEETING WITH AA & WHITE RURAL SOUTHERN ELDERS & FAMILIES

3. DISCUSSING NURSING HOMES, HOME HOSPICE & OTHER	RACE	UNDERLYING EXPERIENCES, CULTURAL VAULES & PERSPECTIVES		PROGRAMMATIC IMPLICATIONS OF CAG RECOMMENDATIONS
		AFRICAN AMERICAN	WHITE	
Discussing Nursing Homes	W	N/A	(1) Difficult to take care of loved one at home alone. (2) Guilt about placing loved one in nursing home.	Help CG process their feelings about putting loved one in nursing home
	AA	(1) Family's responsibility to take care of loved one at home, no matter the sacrifice(s). (2) AAs have not had anyone to help them for generations. "We take care of ourselves"	N/A	Don't bring up nursing homes unless asked or unless PT is already resident of nursing home, or about to be discharged to a nursing home per hospitalist orders.
				If loved one is going to nursing home, provide support to family.
Discussing Home Hospice	W & AA	Hospice = death		(1) Don't use word 'hospice'. (2) Ask where PT would like to be, & where would be most comfortable. (3) Focus on helpful things that home health can provide to PT/family.
				If home hospice is brought up by family, explain that it is 'extra' help for PT/family to help out during difficult time.
	AA	(1) We don't talk about death; (2) Family's responsibility to take care of loved one at home, no matter the sacrifice(s); (3) AAs have not had anyone to help them for generations. "We take care of ourselves" .	N/A	Don't raise issue or possibility of home "hospice".
				Ask if needs help with caring for PT at home, and if so, what kind of help. If is kind of care home hospice provides, explain that this is the type of care that home health/hospice provides.
				(1) Stress that home health/hospice are not there to take over; family is in charge of making all decisions & determining how things are done. (2) Ask if concerns about this kind of care at home; listen and discuss until all concerns are alleviated.
				(1) Following discussion, if PT/family wants home health/hospice, ask if they want you to recommend referral to this. (2) Stress that all decisions are up to PT/family. PC MD there to help, not to change way family takes care of loved one.
Discussing Access To Other Needed Community Resources	AA	(1) Significant lack of awareness of exisiting services (e.g. Transportation, hearing aids) & how to access. (2) Belief that community services "don't help us."	NA	(1) If PT/family require access to other resources, let them know there are services available (e.g. ramp in the house), that can be free or subsidized that social worker (SW) can share with them. Ask if want referral to SW. (2) Ask SW to explain that these services help all patients.

<u>C2.1.4 Technology acceptability/feasibility/fidelity: patients and caregivers</u>: Patients and families (especially AAs) appreciated being greeted by CAG members who explained the study and the community's role in developing it. All nine patients/family members completed the Tele-consult. The initial consult lasted 45-55 minutes. Two family members expressed, initial awkwardness that rapidly dissipated with palliative physician reassurance.

<u>C2.1.5 Technology issues that have been overcome for the proposed study</u>: During the pilot we identified a number of technology issues that were overcome and rectified for the current study: Wi-Fi connectivity, screen freezing, computer /software compatibility; staff interruptions during the consult. Our Tele-Health Medical Consultants have successfully addressed the technology issues (see Section C3.3).

<u>C2.1.6 Palliative physician training and protocol adherence/fidelity</u>: The palliative physician participated in all CAG meetings during the cultural adaptation and protocol development. He was trained in translating CAG experiences into culturally-based Tele-Consult communication practices. During the pilot, the study coordinator, who was present during the Tele-Consult, used a fidelity adherence check sheet to record protocol adherence. In all but one instance, the palliative physician adhered to the Tele-Consult cultural guidelines. Per protocol, this was discussed and no further deviations were noted.

<u>C2.1.7 Data Collection</u>: The study coordinator was able to collect all patient and family member data at all time-points in hospital or via phone follow up after discharge including chart reviews. One patient was lost to follow-up and additional procedures were implemented to prevent future losses.

<u>C2.1.8 Acceptability of Palliative Care by Telehealth</u>: Thousands of ENABLE patients and caregivers demonstrated acceptability and benefit from palliative care telephone coaching. A recent survey study of cancer caregivers indicated a preference for visualizing their providers via videoconferencing vs. phone.[37]

<u>C2.1.9 Collection of Measures</u>: All measures have been found to be acceptable in prior studies.[12-37]

<u>C2.1.10 Conducting Multi-site Study and Recruitment</u>: We have learned many valuable strategies that have allowed us to manage multi-site studies and successfully recruit the requisite patients for 3 palliative care RCTs, and multiple pilot studies. Dr. Bakitas' in progress RCT in patients with heart failure will likely finish recruitment of 380 subjects early and is 60% AA.[103]

C3 Research Team and Locations

C3.1 Research Team

Collaboration history: The multi-PIs, (Elk, Bakitas) are leading a research team with a long history of collaboration representing expertise in palliative care (Tucker, Hauser, Graham), developing models of culturally-based care using CBPR in rural and underserved communities (Elk, Bakitas, MK-S, Levkoff) multi-center RCTs (Bakitas, Elk), geriatrics (Levkoff), hospital medicine (Rhodes, Unnikrishnan, Sonaike) and telehealth (Wallace, McElligott).

C3.2 Locations: The U. of SC (Elk) will serve as the primary coordinating center for study operations and the UAB (Bakitas) site will serve as the primary coordinating center for tele-consult and clinical operations. The 3 study sites are rural hospitals in the Deep South. These hospitals were chosen as they represent the ultimate target for intervention dissemination--they have no access to palliative care, are relatively small with little chance to ever support an interdisciplinary palliative care team; over 15% of their patient population is over 65, and approximately one-fifth are living in poverty and are uninsured (see Environment). These hospitals are their communities' essential health care hub.

Aiken Regional Medical Center, Aiken, SC is a 245-bed acute care facility, owned and operated by a subsidiary of Universal Health Services, Inc. It serves a population of 165,000.

Russell Medical Center, Alexander City, AL: is an 80-bed, not-for profit acute care facility serving the needs of east Central Alabama.

Highland Community Hospital, Picayune MS: is a 60-bed, full-service hospital, serving the community for over 50 years, it is Pearl River County's only acute-care medical facility.

C3.3 Telehealth expertise/resources: Dr. Eric Wallace, UAB Telehealth Medical director, and Dr. James McElligott, MUSC, Telehealth Medical director, are co-leading the technical aspects of the Tele-consult intervention at all sites.

Aiken Regional Medical Center, SC has existing approved telehealth infrastructure that will be used for this proposal. Aiken is supported by the state-appropriated South Carolina Telehealth Alliance (SCTA), including procuring, installing and supporting telehealth equipment. SCTA ensures the equipment is appropriate for the intended use, is standards-based to facilitate an open-access network, and is implemented in coordination with local IT staff. SCTA will provide Aiken with ongoing IT and clinical support. All telehealth endpoints will be compatible with Cisco infrastructure as described for UAB & MI.

UAB will oversee the Russell and Highland Hospital Telehealth programs. These facilities will use Cisco Jabber, a videoconferencing platform in conjunction with Cisco videoconferencing infrastructure and secure servers located within the UAB firewall. All sites' physicians will undergo telehealth privilege and credentialing procedures which includes privacy and equipment training, and will be registered to the UAB Cisco Call Manager allowing access to the UAB telemedicine network for conferencing and documentation. All videoconferencing will be fully encrypted, HIPAA and Joint Commission compliant and in compliance with federal and regulatory requirements.

C4.The 2-Phase RCT Study Overview and Procedures

C4.1 Phase I: Developing Site-Specific CAGs:

Site-specific, community-based CAGs will be developed as integral members of the study team. Building on the R21 CBPR approach, a CAG is key to developing the final aspects of the study design and procedures. Potential CAG members (8, with equal numbers of AA and W) will be identified by hospital staff and community leaders, and invited to participate in study development. CAG members will include respected members of the community, and those who have recently lost a loved one and understand end of life care. CAG members have three key roles:

1. Informing study team about Community Culture: CAG members will meet with and be trained by the southern rural AA and W Beaufort, SC CAG members who developed this protocol. We anticipate that the local CAG members will appreciate and recognize how their involvement will be instrumental in creating a program that could benefit their community.

2. Enlisting CAG Participation in study design: CAG members will meet face-to-face with the study team initially and every three months to receive orientation and training in all proposed study procedures. Their input will be incorporated and the study team will regularly report on progress, and challenges, and will seek advice when problems arise. (No patient information will be discussed.)

3. Introducing Patients to the Study: CAG members will be the first to meet potential study participants (*prior to randomization*) to introduce potential participants to the study. As described above (C2.1.2) They will be

compensated for time and travel for each meeting they attend and each time they approach a patient to explain the study (see Budget Justification).

C4.2 Phase II: Conducting the RCT

C4.2.1 Study Sample: Eligibility Criteria and Justification

Patient Inclusion Criteria:
(1) AA or W;
(2) ≥ 65 years old; has a condition which fits into one of 3 illness paradigms -cancer, chronic progressive, frailty
(3) Clinician answers "no" to question: "Would you be surprised if this person died in the next 12 months?"
(4) Patient has a caregiver who has been involved in their care.
(5) Able to complete baseline interviews.

Patient Exclusion Criteria:
(1) Unable to complete baseline interviews;
(2) Currently receiving hospice care;
(3) No family member/caregiver.

To increase rigor and transparency according to NOT-OD-16-012, we address the following issues:

Biological variables of age and gender. In our pilot study, the intervention has been successfully used with older adults, both genders, and all racial/ethnic groups.

Inclusion/Exclusion criteria rationale - Illness paradigms: To enhance external study validity eligible patients will represent one of 3 illness trajectories as defined by Lunney et al.[104] This paradigm, rather than specific disease is commonly used by palliative care experts to target palliative care interventions and to guide goals of care discussions.

The "surprise" question has been found to be a reliable predictor of patients most likely to benefit from palliative care. It has a sensitivity of 61 to 84% and a specificity of 61 to 90% for identifying those who die in the subsequent year.[105,106]

Has a caregiver: We are interested in the impact of palliative care impact on caregivers. Patients with and without caregivers have differential issues relative to ability to be discharged and/or receive hospice care due to Medicare requirements. Receiving hospice care-Hospice provides home-based palliative care so providing a teleconsult would be a duplication of services.

Completion of baseline interviews: Since patient symptom burden is the primary outcome and is most accurately measured as self-report, patients must be able to complete a baseline measure.

C4.2.2 RCT Estimated Enrollment Rate: We anticipate a total enrollment of 352 participants in the study. Half of the patient participants (N=176) will be randomized to receive usual care appropriate to their illness and half (N=176) will receive the culturally-based Tele-consult in addition to their usual care.

Table. Recruitment Estimates by Hospital

Participating Hospital	no. beds	Annual Admissions	Observed Eligible Patients* (annualized)	Estimated Annual Consults**	Annual recruitment assuming 50% refusal (range of observed - estimated)
Aiken Hospital, SC	243	3767	264	188	132-94
Russell Hospital, AL	80	1506	240	109	54
Highland Hospital, MS	33	1200	296	51	148-25

*Hospitals estimated eligible patients based on study eligibility criteria
**CAPC estimates of consults based on 5-7% of annual admissions

C4.2.3 Justification of Enrollment Estimates: (*Table)* We have worked closely with the study hospitalists and administrators including doing a small pilot in which *actual* hospital admissions were reviewed for a month to identify volume and racial diversity of patients meeting eligible criteria.

C5. Recruitment and Enrollment Procedures:

C5.1 Recruitment: We will employ methods that we were successful in our prior pilot studies and RCTs.[22,24,26,27] Key to successful recruitment, and as described in our 3 study hospitals' leadership and chief hospitalists LOS, is engagement of the referring clinicians in the study from the outset. To this end, since our initial submission the PIs have been fully engaged with these sites and the chief hospitalists are now study co-investigators. They have been actively involved in the development of study procedures and are fully acquainted and in agreement with the study palliative care physicians, study team, and study goals. As described above, they have prospectively done a real-time review of admissions to test their eligibility estimates and consider their commitment of effort to make the study a success. Each site will have two part-time study coordinators: a) Coordinating Study Coordinator (CSC) will screen, recruit, obtain consent, and organize the tele-consult. CSCs will remain in daily contact with hospital staff and participate in team meetings to remind clinicians about

eligibility criteria. Team meeting attendance will allow the CSC to clarify the study goals and protocol and to strategize solutions if challenges arise. b) Blinded Study Coordinator (BSC) -The blinded study coordinator will collect all data following patient randomization.

C5.2 Consent: a) Approaching the patient and family: The CSC will screen admissions each weekday and will only approach potential study participants after confirming eligibility (e.g. “surprise question”) and getting approval from the treating clinician. b) CAG and CSC meet with patient and family: Following approval, the CSC will contact the CAG member “on-call” for the day to speak with the patient. The CAG member and CSC will approach the patient. The CAG member will introduce themselves and the CSC, and describe the study to the patient. The CAG will emphasize their role in developing the study and how this study was designed to help the community. If the patient indicates that he/she would like to participate, the CSC will provide an overview of the study, including a description of palliative care, the tele-consult equipment and process, (if randomized to that group), and randomization using a simple visual diagram. (These materials were developed by the R21 CAG and will be reviewed and adapted by each hospital-specific CAG). The CSC will then read the consent form to all patients (assuming no or limited literacy) unless the patient prefers to read it themselves. The family member is invited to participate and will sign a separate consent agreeing to complete questionnaires regardless of group assignment.
3. Baseline Data Collection**:** The CSC will assist the patient (and separately the family member) to complete the baseline questionnaires using an electronic tablet that connects directly to the RedCap database. Baseline data are collected prior to randomization, so study coordinator blinding is not necessary.

C6. Randomization
Scheme. The randomization scheme will be executed via RedCap, a clinical trials management software program we have used to facilitate randomization in other studies. Participants will be randomly assigned to group (1:1) using a computer-generated program overseen by Dr. Hardin. The randomization scheme will be stratified by site (SC, AL, MS) race (W, AA), and illness trajectory as described by Lunney et al (e.g., terminal illness, organ failure, frailty).
Process and blinding. As the Central Coordinating Site, USC will manage the randomization process. The USC project manager will be alerted to the assignment by RedCap and will trigger the local unblinded study coordinator to communicate assignment to the participant and initiate study protocol. All other members of the research team will remain blind to group assignment and participants will be instructed not to discuss their assignment with the local study coordinator collecting the outcome assessments.

C7. Intervention and Usual Care Conditions

C7.1 Theoretical Background of the Tele-consult Intervention

Figure 3. TELE-CONSULT CONCEPTUAL MODEL

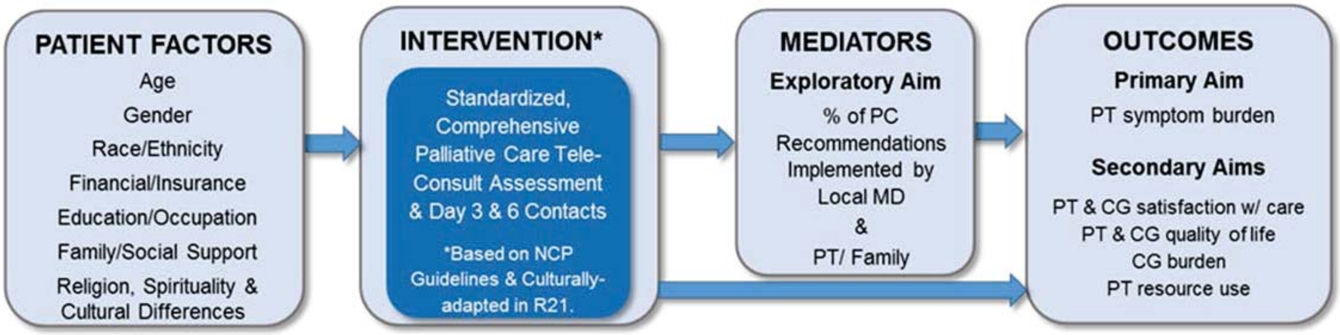

This investigator-developed conceptual model (*Figure 3)* guides the proposed study to examine the effects of a community-developed, culturally-based palliative care Tele-consult on patient symptom burden (primary outcome) and on patient and caregiver QoL, satisfaction with care, caregiver burden, and resource use (e.g. 30-day hospital readmissions/days, ICU, ED visits & hospice use) following discharge (secondary outcomes). The intervention comprises a standardized palliative care assessment based on the National Consensus Project for Quality Palliative Care framework that incorporates the 8 domains of providing comprehensive care.[38] Central to the Tele-consult intervention, based on our pilot R21 are the community-developed, culturally-based communication approaches, and follow up (Tables 1-3 and *C2. Preliminary Studies*). Recognition of, and respect for cultural differences form the basis of this unique tele-consult program. Study

palliative care physicians will receive training in these culturally-appropriate communication strategies. We will examine the mediating and moderating role of the local hospitalist and patient/family participant's implementation of the palliative physician's recommendations on patients' symptom burden and family member's care burden (exploratory analysis).

C7.2 Tele-consult Intervention

The Tele-consult intervention (Table 4) consists of ***3 interactions between the remote palliative care physician/interdisciplinary team (IDT) and the local patient/caregiver and hospitalist/treatment team***.

TABLE 4: Community-developed, Culturally-Based Inpatient Tele-consult Intervention

Contact #	When	With Whom	Method	Purpose
#1 Palliative Care Tele-Consult	As soon after randomization as possible	Patient (family) referring hospitalist & remote palliative clinician Local study coordinator Remote IDT Members	Secure tele-consult Remote IDT review eHR document	1. Conduct community-developed, culturally-based palliative assessment. 2. IDT review. 3. Develop recommendations & palliative care plan.
#2 Follow up Day 3	Within 3 days post consult	Remote & local clinicians	eHR document Telephone	1. Provide recommendations & care plan. 2. Respond to patient, family & remote team questions. 3. Identify community care team & initiate referrals as needed (including primary clinician/hospice provider if appropriate).
#3 Follow up Day 6	Within 6 days post consult	Remote Clinician Patient/family & local community care team members	Telephone	1. Reassess patient and care plan. 2. Assess adequacy of discharge plan or home experience. 3. Refer to community resources.

Contact #1 As soon after randomization as possible: The remote palliative care physician will conduct the community-developed, culturally-based Tele-consult using the communication methods that correspond to the expressed preferences of either AA or W or both rural southern community members (Tables 1-3). These recommended communication methods apply primarily to the palliative physician and other study team members (site coordinators and CAG members) who come into contact with the patient. All three groups will receive training in the specific aspects of this culturally-based communication protocol (see Training (C6.7.2.1).
Setting up Tele-Consult: The CSC will work closely with the patient/family, local team, and remote palliative care physician to determine a time suitable (within 48 hours on weekdays) to perform the Tele-consult. The CSC and the hospitalist/a member of the treatment team will be in the room with the patient and family. The CSC will set up the HIPAA protected telehealth laptop next to the patient's bed, introduce the patient to the remote palliative physician, and the tele-consult will proceed. All Tele-Consults are digitally-recorded within the secure portal. Most consults take 45-60 minutes. To ensure a comfortable conversation with minimal interruptions we have instituted several steps including checking with floor nurse, putting up signs on the door, and tasking the CSC with running interference in case of interruptions.
Post-Tele-Consult: Within 24 hours of conducting the Tele-consult, the remote palliative physician will seek an interdisciplinary team review, develop the plan, and make recommendations which may include immediate patient care (e.g., symptom relief) and continuing care, related to the hospital stay and following discharge. Recommendations may also include referrals to other supportive resources such as a hospital social worker or pastor. The written consult will be documented using a standardized note template that corresponds to the 8 NCP palliative care domains and will be provided via a secure HIPAA-compliant portal that will be part of the patients' inpatient medical record. As needed, verbal contact will also occur between the palliative care physician and the hospitalist staff.

Contact #2 Day 3 (approximately 24-48 hours after first contact). The CSC will arrange a follow up videoconference between the remote palliative care physician and patient if in hospital or via phone if the patient has been discharged. The purpose of this second contact is to determine how patient is progressing, if the plan was implemented, if hospital staff/patient has encountered any challenges or questions, and to determine who will be assuming the patient's care in the community. Documentation of the contact will be provided as described above.
Contact #3 Day 6 (post consult) (via videoconference if patient is in hospital, via phone if discharged.) To determine if patient is experiencing new or ongoing challenges, to confirm availability of community or hospice care as appropriate. Documentation of the contact will be provided as described above.

C7.2.1 Training and Supervision: The remote palliative care physicians and the study coordinator will convene in Columbia, SC to receive an 8-hour training in the culturally-based Tele-consult and follow up protocol. This will include meeting with the R21 CAG and study co-I, Dr. Josh Hauser. The standardized training will include all aspects of the communication strategies with special attention to "cultural competence training" using role-play and feedback sessions.
The co-PIs will train Project Manager and study coordinators in all study procedures based on a study manual developed during the R21 (in accordance with NOT-OD-17-035, the treatment protocol has not been included in the appendices). CAG members at 3 hospital sites will be trained by the co-PIs at their local facilities and through a videoconference meeting with the R21 CAG members. Training will cover how the program was designed and developed by the community and also the background behind cultural recommendations. R21 and local CAG members will receive compensation for their participation as outlined in the Budget Justification.

C7.2.2 Teleconsult Intervention Fidelity monitoring: During the study, a trained palliative care clinician not associated with the study will use a fidelity checklist similar to those used in our prior studies to monitor the palliative physicians' adherence to the study protocol by rating the digital audio recording of the Tele-consult sessions and the consult documentation. Fifteen percent of randomly selected participants will have all three of their protocolized contacts reviewed. If ratings are below the "satisfactory" rating on the scales, supervision will focus on necessary techniques to improve the score to satisfactory or higher.

C7.3 Usual Care: Patients randomized to usual care in each of the three participating hospitals will receive inpatient care appropriate to their illness. This includes assessment and treatment by the admitting physician, along with any subspecialists that are consulted. The formulation and sharing of prognostic information and the engagement of patients and families in establishing goals of care will be according to the standards of the admitting physician and subspecialists. Treating clinicians may include hospitalists, nurse practitioners, social workers, pastoral care, and occasionally, the patient's primary care practitioners who have hospital privileges. Usual Care monitoring: We will review usual care patients' medical records during hospitalization and for 30 days subsequent and will document the patients' use of any supportive care or medical services during that time. This will allow us to track practice, care and resource use patterns over time to examine temporal trends or changes and to identify any potential contamination, such as any increase in supportive care services, hospice, etc. over the course of the study.

C8.INSTRUMENTS and DATA COLLECTION AND MANAGEMENT

C8.1 Instruments: All instruments were chosen to measure distinct constructs within the conceptual model. These instruments have been used in the pilot R21 and our previous studies[22,24,26,27]and presented minimal burden to patient and family (Table 5).

C8.2 Data Collection and Management: The BSCs will be trained to collect baseline, Day 7 and Day 30 post discharge medical record, Tele-Consult documentation, and patient and caregiver reported data in the hospital and by phone following discharge in a study-specific RedCap database that will be housed at USC. REdCap is a secure web application for building and managing online surveys and databases. It is HIPPA compliant and password protected in both the front end and back end (See Facilities/Resources). The database will track the participants' progress through recruitment, enrollment and all study procedures. The database comprises:
a) Patient and Family Member Contact, Consent, and Demographics: These forms will include patient and family contact information, study status (enrolled, declined, etc.), demographics.
b) Patient and Family Member-reported Outcome Measures for each data collection time point (Table 5): Patient symptom burden (Primary Aim) and patient/ caregiver-reported responses (Secondary Aims).
c) Resource Use: hospital readmissions (# hospital/ICU days), emergency department (ED) visits, and hospice days during the 30 days following

Table 5. Measures and Data Collection Schedule

Construct	Instrument & Description	# of Items	T1 Baseline	T2 Day 7	T3 Day 30
Demographics	Demographic Questionnaire- Age, gender, race/ethnicity, marital status, religion, education, occupation, insurance status, financial status.	10	X		
Primary Aim					
Symptom Burden	**Edmonton Symptom Assessment Scale (ESAS)** - symptom intensity using visual analog	9	X	X	
Secondary Aims					
Patient and Caregiver QoL	**PROMIS Global Health -10**. Evaluates physical, social, and emotional health in healthy & chronically ill adults	10	X	X	
Family Satisfaction with Care	**FamCare** - family satisfaction with availability of care, physical & psychosocial care, information giving	20	X	X	
Patient Satisfaction with Care	**Feeling Heard and Understood** - Self-report quality measures for palliative care settings on Likert scale (1 item)	1	X	X	
Caregiver Burden	**Montgomery Borgatta Caregiver Burden Scale (MBCB)**-subscales objective, subjective, demand burden	14	X	X	
Resource Use	**Hospital admission, ED visit, hospice**	NA			X
Exploratory Aim					
Mediators	Consult recommendations implemented by Hospitalist & Pt	NA		X	

discharge will be collected via eHR and patient/family report (Secondary Aims).
d) eHR and Palliative Physician Tele-consult Note Documentation and Recommendations: Pertinent clinical documentation & remote palliative care physician recommendations and the extent to which they were implemented by the treating clinician, patient/family (Exploratory Aim).

D. STATISTICAL CONSIDERATIONS:

D.1 Sample Size: Assuming a uniform accrual of patients, we will enroll 352 patients stratified by site and race using a block randomization method into two groups (176 per treatment group). This sample size is similar to a study led by Bakitas et al. which enrolled n=322 patients and which had sufficient power to detect and report meaningful results[23] in measures such as quality of life, symptom intensity, and depression scores. Our sample size will result in 80% power at 0.025 (to adjust for multiple comparisons in interaction models of Aim 2) to detect a 0.33 standardized effect; for reference, 0.20 is a small standardized effect size and 0.50 is a medium standardized effect size,[107] and we include additional information on sample sizes in Table 6. Even if we fall short of enrollment by as much as 20%, we will still be powered to detect a standardized effect between 0.33 and 0.37. Distinguishing standardized effects in specific terms of measures in this study, we will have 80% power to detect a difference of 3.6 in average scores of QoL between groups where the Bakitas et al study observed a difference of 4.1 in average scores of QoL. Other differences are similarly comparable for our planned sample size.

Table 6: Sample Size Calculation

Standardized effect size	Required sample per treatment group
0.20 (small)	380
0.30	176
0.40	98
0.50 (medium)	63

D.2 Statistical Analyses: Demographic variables (e.g., age, sex, race, disease) will be summarized and compared across treatment groups using t-tests and nonparametric Wilcoxon signed rank tests for continuous measures (age), binomial tests (sex, race), and chi-square statistics for categorical variables (e.g., marital status, education). All demographics and stratification variables found to differ across treatment groups will be included in regression models (referred to as the vector Z_i in the regression models to follow); however, race will be included in the final models separately from the vector Z_i vector. Tabulations of binary and categorical variables will be presented for all measures and broken down by treatment group. Age will be summarized by mean and standard deviation for all participants, and broken down by treatment group.

Primary Aim 1: Determine whether a community-developed, culturally-based palliative care tele-consultation program leads to lower symptom burden in hospitalized African American and White older adults with a life-limiting illness. *H1: Intervention patient participants receiving a* culturally-based palliative care tele-consultation program *will experience lower symptom burden 7 days after the consult.*

Scores for symptom burden (measured by ESAS) and outcomes for Aim 2 will be assessed for change in regression models for which the baseline score will be utilized as a covariate. The generalized linear model (GLM) framework given by: $y_i = g^{-1}(\eta) = g^{-1}(\beta_0 + Trt_i\beta_1 + (Trt * Race)_i\beta_2 + Z_i\beta_3 + Baseline_i\beta_4)$ where y_i is the follow up outcome of interest, Z_i is a vector of covariates for which models must be adjusted, $Baseline_i$ is the baseline outcome score, and g is an invertible link function equating the condition mean of the outcome to the linear predictor η. For each measure, the i refers to the participant. In these GLMs, we are interested in whether there is a difference in the outcomes for treatment groups, and whether there is a differential impact of treatment for each race. Respectively, these differences will be assessed by the Wald tests of $H_0: \beta_1 = 0$ and $H_0: \beta_2 = 0$. The latter comparison will be evaluated first and if not found to be significant, a model without this term will be estimated prior to interpretation of the hypothesis test of $H_o\ \beta_1 = 0$. GLMs will utilize canonical link functions for outcomes: log link for count outcomes, identity link for continuous outcomes, and logit link for binary outcomes. We will also estimate log-links for binary outcomes so that exponentiated estimated coefficients may be interpreted as estimated risk ratios.

Aim 2: ***Secondary Aim:*** Determine whether a culturally-based palliative care Tele-consult program results in higher patient and caregiver quality of life, care satisfaction, and lower caregiver burden at Day 7 post-consultation, and lower resource use (hospital re-admission, emergency visits) 30-days post-discharge.

***H2**: Intervention participants and their caregivers receiving a culturally-based palliative care Tele-consult program will experience higher patient and caregiver quality of life, care satisfaction, lower caregiver burden at Day 7 post-consultation, and lower resource use (e.g., hospital admission, emergency visits) at 30 days after discharge.*

Scores for measures (patient satisfaction with care, caregiver satisfaction with care, patient QoL, caregiver QoL, Resource Use (hospital admissions-measured from the day of patient's hospital discharge until 30 days-later and number of emergency room visits) will be analyzed with the strategy described for Aim 1. For resource outcomes (readmissions and emergency room visits), the model will be given by: $y_i = g^{-1}(\eta) =$

$g^{-1}(\beta_0 + Trt_i\beta_1 + (Trt * Race)_i\beta_2 + Z_i\beta_3 + Baseline_i\beta_4 + Exposure_i)$ where g, y_i, Z_i, and $Baseline_i$ are the same as described above for Aim 1 and $Exposure_i$ is the number of days under observation. A False Discovery Rate (FDR) approach will be used to control for multiple inferences in the same body of data.

Exploratory Aims: Mediation analysis will follow the approach of Kohler, Karlson, and Breen.[108] This approach is an alternative to the usual Baron and Kenny[109] approach that improves results for nonlinear models. Possible binary mediators include the implementation level of palliative care physician recommendations by (1) the treating clinician, and (2) the patient/family. Using this approach, we will infer results that are decomposed into direct and indirect effects. If there is no evidence of mediation, only direct effects will be estimated in appropriate models.

E. POTENTIAL PROBLEMS AND ALTERNATIVE STRATEGIES

We have anticipated potential problems and solutions to the following:
1. Recruitment: Based on the numbers of patients and the penetration rate, we anticipate sufficient numbers of eligible patients for our study. The main barrier to recruitment we identified in pilot study was that hospitalists not providing permission to approach patients whom they considered not “ready” for palliative care. However, once the PC physician built a relationship with these treating clinicians and directly communicated with them about a potential patient, they recognized the potential benefits of PC at all stages of illness. We have taken 3 steps: (a) Invited the chief hospitalist from each hospital to be Co-Is on the research team; all have enthusiastically agreed; (b) the hospitalist/member of treatment team will participate in the first PC contact, and (c) the PC physician will make telephonic contact with the hospitalist a day or two after first consultation to discuss patient progress. These additional steps will ensure hospitalist participation in both patient referral and in ensuring continuity of care. The CSC will meet daily with other hospital staff, attend meetings of nurses or care coordinators, remind them of study criteria and thank them for referrals. At the ongoing Research Team meetings, the PIs, the Project Manager, SCs and CAG members will monitor recruitment and jointly problem solve.
2. Usual Care Contamination: is possible since treating clinicians will be treating patients in both groups, more likely in small hospitals. However: (a) Our study is not directed at changing care provider behavior. Prior studies have shown that simply exposing physicians to new practices and providing education are not sufficient to change physician behavior[110] so while control group contamination due to treating clinician behavior change is possible, it is extremely unlikely. (b) PC interventions are multi-faceted and individually-directed so that it is unlikely that a clinician will have exposure to all the components. (c) Because the hospitalists will now be members of the research team, they are more likely to adhere to the study’s requirements.
3. Feasibility: Feasibility of patient and family acceptance of participating in a PC consult study were confirmed in the pilot study. Concern had been expressed by the CAG that AA patients and family would not want to participate. Their recommendation, and our implementation of CAG members of the same race being the first to approach patients and share how the community developed the program, eased concerns. We anticipate that this same principle of CAG members reaching out to potential patients will lead to patients wishing to participate in the study. Dr. Bakitas has over 20 years of experience in conducting RCTs. She will be responsible for ensuring that all aspects of the study adhere to state-of-the art RCT guidelines, and she and Dr. Elk will problem-solve issues that arise together.
4. Non-participation bias: Non-participant bias will be monitored by collecting demographic data (race, sex) on those who decline and comparing it to patients who participate.
5. Respondent bias: Post-randomization data will be collected only by blinded study coordinators.

F. STUDY TIMELINE

	YR1				YR2				YR3				YR4				YR5			
ACTIVITY	Q1	Q2	Q3	Q4	Q1	Q2	Q3	Q4	Q1	Q2	Q3	Q4	Q1	Q2	Q3	Q4	Q1	Q2	Q3	Q4
Hire, train staff, IRB approval																				
Establish CAG, provide orientation &training																				
Build relationships w hospital staff, orient to study & eligibility criteria																				
CAG quarterly meetings																				
PC physician training in Culturally-based tele-consult																				
Patient enrolment																				
Data Collection																				
Data Analysis																				
Progress Reports/Final Reports																				
Presentations and Publications																				
