# Supplementary material for: Palliative Video Consultation and Symptom Distress Among Rural Inpatients: A Randomized Clinical Trial
Source: JAMA Netw Open. 2025 Jul 9;8(7):e2519426. doi: 10.1001/jamanetworkopen.2025.19426 (PMC12242707; doi:10.1001/jamanetworkopen.2025.19426)
Supplement: Supplement 2. — eAppendix 1. EMR Screening for Medical Conditions eTable 1. Study Sites eTable 2. Culturally Based Consultation Guidelines Linked to NCP Consultation Guidelines eAppendix 2. Palliative Care Consultation Documentation Template eAppendix 3. Community Tele-Pal Videoconsultation Fidelity Monitoring Process eTable 3. Characteristics of Patients Who Consented vs Declined eTable 4. Caregiver Baseline Demographics and Outcomes eTable 5. Comparison of Demographics of Intervention Patients Who Completed and Did Not Complete Videoconsultation eTable 6. Patient Satisfaction With Videoconsultation Technology and Process eTable 7. ESAS Total Symptom Distress, Intervention vs Usual Care, Adjusted for Baseline eTable 8. Caregiver Burden, QOL, and FAMCARE From Baseline to 30 Days, Intervention vs Usual Care eTable 9. Caregiver Heard and Understood From Baseline to 30 Days, Intervention vs Usual Care eFigure 1. Patient Satisfaction With Technology eFigure 2. Patients Feeling Heard and Understood [file jamanetwopen-e2519426-s002.pdf]

# Supplementary Online Content

Bakitas MA, Gazaway S, Underwood F, et al. Palliative videoconsultation and symptom distress among rural inpatients: a randomized clinical trial. *JAMA Netw Open*. 2025;8(7):e2519426. doi:10.1001/jamanetworkopen.2025.19426

**eAppendix 1.** EMR Screening for Medical Conditions

**eTable 1.** Study Sites

**eTable 2.** Culturally Based Consultation Guidelines Linked to NCP Consultation Guidelines

**eAppendix 2.** Palliative Care Consultation Documentation Template

**eAppendix 3.** Community Tele-Pal Videoconsultation Fidelity Monitoring Process

**eTable 3.** Characteristics of Patients Who Consented vs Declined

**eTable 4.** Caregiver Baseline Demographics and Outcomes

**eTable 5.** Comparison of Demographics of Intervention Patients Who Completed and Did Not Complete Videoconsultation

**eTable 6.** Patient Satisfaction With Videoconsultation Technology and Process

**eTable 7.** ESAS Total Symptom Distress, Intervention vs Usual Care, Adjusted for Baseline

**eTable 8.** Caregiver Burden, QOL, and FAMCARE From Baseline to 30 Days, Intervention vs Usual Care

**eTable 9.** Caregiver Heard and Understood From Baseline to 30 Days, Intervention vs Usual Care

**eFigure 1.** Patient Satisfaction With Technology

**eFigure 2.** Patients Feeling Heard and Understood

This supplementary material has been provided by the authors to give readers additional information about their work.

© 2025 Bakitas MA et al. *JAMA Network Open*.

## eAppendix 1

## EMR Screening for Medical Conditions

### Dementia/Alzheimer's Disease Exclusion Criteria

1) DEMENTIA (any: Alzheimer's, Vascular, Lewy Body, Frontotemporal)—Exclusion
***Patients with any diagnosis of dementia, are not eligible. HOWEVER, a diagnosis of Mild Cognitive Impairment is NOT an exclusion. These patients can proceed to next stage (in-person screening) if they are otherwise eligible. If "memory loss noted" or diagnosis unclear, choose "No". in-person screen includes cognitive screen (Callahan Six-Item Screener).

Hints: Any dementia: Senile dementia, Alzheimer's, Vascular dementia, Frontotemporal dementia, Dementia with Lewy bodies. May also see diagnoses associated with dementia like Parkinson's disease with dementia.

- Applicable diagnoses
  - Vascular dementia
  - Dementia in other diseases classified elsewhere
  - Unspecified dementia
  - Alzheimer's disease
  - Other degenerative diseases of nervous system, not elsewhere classified
    - Frontotemporal dementia
    - Pick's disease
    - Other frontotemporal dementia
    - Senile degeneration of brain, not elsewhere classified
    - Other specified degenerative diseases of nervous system
    - Dementia with Lewy bodies

### CHRONIC ILLNESS INCLUSION CRITERIA

1) CANCER

Hint: Metastatic cancer has spread from original organ to another site. Does NOT include skin cancer. If patient has diagnosis of cancer in problem list, may need to check last PCP note or oncology note to determine stage.

- Metastatic Cancer of Solid Organs (Stage IV)
- Hematologic malignancies: These are malignancies of blood and lymphoid systems. These include:
  - Lymphomas
  - Leukemias
  - Multiple myeloma
  - Myelodysplastic syndrome
  - Malignant neoplasms of lymphoid, hematopoietic and related tissue
  - Other neoplasms of uncertain behavior of lymphoid, hematopoietic and related



© 2025 Bakitas MA et al. *JAMA Network Open*.

tissue

2) **CARDIAC DISEASE**

- Coronary Artery Disease (CAD)
- Peripherial artery disease
- Carotid artery disease
- Ischemic heart disease
- Angina pectoris
- Acute myocardial infarction
- Subsequent ST elevation (STEMI) and non-ST elevation (NSTEMI) myocardial infarction
- Other acute ischemic heart disease
- Chronic ischemic heart disease
- Peripheral vascular disease
- Atherosclerosis of native arteries of the extremities
- Other peripheral vascular diseases
- Rheumatic heart disease
- Heart failure
- Congestive heart failure
- Deep vein thrombosis
- Chronic high blood pressure

3) **PULMONARY DISEASE**

| Hint: These are chronic lower respiratory diseases. May include the following: Chronic bronchitis, COPD, asthma, emphysema, bronchiectasis |
|---|

- Chronic Obstructive Pulmonary Disease (COPD)
- Bronchitis, not specified as acute or chronic (NOTE: Acute bronchitis does not meet this eligibility criterion; will need to find evidence of chronic disease).
- Simple and mucopurulent chronic bronchitis
- Unspecified chronic bronchitis
- Emphysema
- Other chronic obstructive pulmonary disease
- Pulmonary embolism
- Asthma
- Bronchiectasis
- interstitial pulmonary disease
- interstitial lung disease (ILD)
- pulmonary fibrosis
- interstitial pneumonitis
- cryptogenic organizing pneumonia
- idiopathic interstitial pneumonia
- Other interstitial pulmonary diseases (ALL)

4) **NEURO-DEGENERATIVE DISEASE**

- Parkinson's disease



© 2025 Bakitas MA et al. *JAMA Network Open*.

- Amyotrophic lateral sclerosis (ALS/Lou Gehrig's). In both cases, Advanced Parkinson's and ALS, patients not likely to be able to physically take part in study.
- Huntington's disease
- Spinal muscular atrophy
- Motor neuron disease

5) RENAL DISEASE
    - Chronic kidney disease (CKD)
    - End-stage renal disease (ESRD)
    - Unspecified kidney failure
    - Type 1 diabetes mellitus with circulatory complications
        - Type 1 diabetes mellitus with diabetic peripheral angiopathy without gangrene
        - Type 1 diabetes mellitus with diabetic peripheral angiopathy with gangrene
        - Type 1 diabetes mellitus with other circulatory complications
    - Type 1 diabetes mellitus with kidney complications
        - Type 1 diabetes mellitus with diabetic nephropathy
        - Type 1 diabetes mellitus with diabetic chronic kidney disease
        - Type 1 diabetes mellitus with other diabetic kidney complication
    - Type 2 diabetes mellitus with circulatory complications
        - Type 2 diabetes mellitus with diabetic peripheral angiopathy without gangrene
        - Type 2 diabetes mellitus with diabetic peripheral angiopathy with gangrene
        - Type 2 diabetes mellitus with other circulatory complications
    - Type 2 diabetes mellitus with kidney complications
        - Type 2 diabetes mellitus with diabetic nephropathy
        - Type 2 diabetes mellitus with diabetic chronic kidney disease
        - Type 2 diabetes mellitus with other diabetic kidney complication

6) STROKE

7) SEPSIS

8) HEPATIC DISEASE

| |
|---|
| **Hint:** Can be liver disease from any of the causes listed below—fibrosis, sclerosis, cirrhosis, hepatic failure, liver failure |

- Alcoholic liver disease
    - Alcoholic fibrosis and sclerosis of liver
    - Alcoholic cirrhosis of liver
    - Alcoholic hepatic failure
- Toxic liver disease
    - Toxic liver disease with hepatic necrosis
    - Toxic liver disease with fibrosis and cirrhosis of liver
- Hepatic failure, not elsewhere classified
    - Chronic hepatic failure
    - Hepatic failure, unspecified

Version date: 4/20/2020

- Fibrosis and cirrhosis of liver (ANY)
- Chronic viral hepatitis
- Fatty liver disease

© 2025 Bakitas MA et al. *JAMA Network Open*.

| eTable 1. Study Sites | |
|---|---|
| **Study Site** | **Description** |
| **Russell Medical Center**<br><br>**Alexander City, AL**: | 80-bed not-for-profit, general medical/surgical acute care facility serving the needs of east central Alabama.<br><br>The medical staff includes 67 physicians and 135 total providers offering a wide spectrum of services. T<br><br>The facility receives approximately 25,000 emergency room visits, admits over 3000 patients and performs over 4000 inpatient and outpatient surgeries each year.<br><br>The facility offers a strong program of community health education services through health screenings, support groups, childbirth classes, self-help programs, and athletic trainers to multiple sports teams in its service area. |
| **Aiken Regional Hospital**<br><br>**Aiken, SC** | 245-bed for-profit acute care facility offering a comprehensive range of specialties and services in east central South Carolina including Aiken and surrounding communities.<br><br>The medical staff consists of more than 120 multi-specialty physicians, and a team of 230 volunteers.<br><br>The facility professionals offer a wide spectrum of more than 50 services including behavioral health and cancer care.<br><br>It receives more than 42,000 emergency room visits and performs approximately 9000 surgeries each year.<br><br>The facility offers numerous educational opportunities including classes, seminars, and support groups. |
| **Anderson Regional Medical Center (ARMC)**<br><br>**Meridian, MS** | 400- bed not-for-profit healthcare facility, serving East Mississippi and West Alabama.<br><br>One of the largest hospitals in the region, offering a full spectrum of 35 services, including cardiology, oncology, orthopedics, emergency care, and women's health provided by over 200 physicians<br><br>The facility receives over 39,000 emergency room visits each year.<br><br>Beyond its clinical services, ARMC plays an active role in community outreach; organizing various health education programs, screenings, and wellness initiatives aimed at promoting public health and improving access to care for underserved populations. |
| **Highland Community Hospital***<br><br>**Picayune, MS** | 60-bed not-for-profit hospital and affiliated clinics provides access to a broad range of services in Southwest Mississippi including Picayune MS and the surrounding areas.<br><br>Over100 physicians are affiliated with the facility.<br><br>The facility also provides significant community outreach which includes classes and events covering different themes, continuing education, screenings, and support groups. |

© 2025 Bakitas MA et al. *JAMA Network Open*.

**eTable 2 Culturally Based Consultation Guidelines Linked to National Consensus Project (NCP) Consult Guidelines**

| **NCP Guidelines* (*Numbering listed per NCP guidelines)** | **Culturally Based Guidelines** | |
|---|---|---|
| | **African American** | **White** |
| | **Understand Distrust (AA)** | |
| | Lack of trust of medical system and care. Recognize and respect that there are historical reasons for this. Work to establish trust. | |
| | <u>**Reduce Distrust**</u>:<br>African American patients/caregivers are more likely to trust African American members of their community.<br>1. All African American patients/caregivers will first meet an African American CAG member who will introduce them to the study (but not review consent).<br>2. If patient/family agree, CAG member will introduce them to the Study Coordinator who will review the consent. | Although lack of trust was not a concern, White patients and caregivers will first meet a CAG member (W or AA) who will introduce them to the study (but not review consent). |
| | **Enhance Trust and Address Telehealth** | |
| | 1. PC physician is not in same facility, pt/family need to have some indication that he/she is a clinician (doctor.) Wear White coat | |
| | 2. Meeting pt/family via telehealth, acknowledge at the beginning of the session that this is not the same as sitting next to one another. | |
| **d. A thorough review of:** 1. Medical records; ii. Relevant lab results | | |
| **e. A review of**: i. Medical history; ii. Therapies; iii. Recommended treatments and iv. Prognosis | | |

© 2025 Bakitas MA et al. *JAMA Network Open*.

| | | |
|---|---|---|
| **f. Identification of:** i. Comorbid medical; ii. Cognitive; iii. Psychiatric disorders | | |
| **g. A medication reconciliation** including over the counter meds | | |
| | **Address Patient and Family:** | |
| | Do not call patient by first name unless invited to do so. | |
| | Never be rude, always be courteous; Always respect patient confidentiality and never share prognosis in a public space, and not in front of non-family members. | |
| | 1. Introduce self (PC physician), then asks patient and caregiver and all else in room to introduce selves. Hospital staff and study coordinator last. | |
| | 2. Acknowledge tele-health medium. | |
| | **Establish Rapport:** | |
| | Get to know the patient, establish rapport | |
| | Take and make time to get to know the patient and the family. | |
| | Learn something the patient's family, e.g. patient's past occupation, where he/she has lived. Repeat it back and converse about it. | |
| | Bring up something local, (e.g. About local geography, local history) to indicate that you know about the area. | |
| **h. Social determinants of health including**: i. Financial vulnerability, housing, nutrition, safety. | **Recognize financial vulnerability:** | |
| | There are many in the African American community and some in the White Community who experience financial hardship. Recognize many experience substantial financial difficulties and the realities this brings. E.g. have realistic expectations; recognize that some things that we may take for granted, e.g. having A/C is not available forall. | |

© 2025 Bakitas MA et al. *JAMA Network Open*.

| | | |
|---|---|---|
| | AA community don't ask and don't know what's available. There is a need for community members to be aware of community resources. (The group developed a brochure specifically aimed at AA to bring awareness of services to AAs. Used AA visuals and large font). | |
| **j. Patient and family emotional and spiritual concerns**, including previous exposure to trauma | **Understand Role of Religion and Church:** | |
| | Pastors are the key to helping us understand prognosis & impending death. If prognosis is to be discussed, suggest that they may want to invite their pastors to the discussion of prognosis. Then ask name of pastor and tell them you'd welcome them to the meeting. | |
| | Religion is the source of all comfort, a key value, and it's the perspective from which African Americans view the world. Therefore, in all PC physician interactions with African American patients recognize and respect that thisis an INTEGRAL part of all that is said and done. | Church members are a source of support for patients and family members. If patient and/or family members need support, ask if a church member can assist. Then ask for name of church member and discuss how they can provide support. |
| **l. Patient and family needs related to** : i. Anticipatory Grief; ii. Loss and Bereavement including assessment of family risk for prolonged grief disorder | **Understand Death and Dying (AA)** | |
| | Death is not discussed in African American church, nor in our homes. Recognize that and approach this topic (death, impending death, possibility of death) with caution. | |
| | No African American person dies alone. If they have no one, a pastor will come and sit with them so that they are not alone during the transition. | |
| **b. Determination of** i. Decision making capacityOR ii. Identification of the person with legal decision making authority | **Understand Family Will Take Care of Loved One (AA)** | |

© 2025 Bakitas MA et al. *JAMA Network Open*.

| | | |
|---|---|---|
| **i. Social and cultural factors and caregiving support including**:<br>i. Caregiver willingness & capacity to meet patient needs | African American families take care of their loved ones themselves in their homes. Even if there is sacrifice, one or other family member will always be there to care for loved one. | |
| **k. The ability of the patient, family and care providers to**: i. Communicate with one another effectively: consideration of language, literacy, hearing and cultural norms | *See also: Understanding Death and Dying* | |
| **a. Pt and family understanding of:** i. Serious illness | **Understand Talking about Prognosis** | |
| | 1. Ask patient/family if want to know prognosis; | 1. Sensitively determine if patient/family want to know about prognosis. |
| | 2. Never be blunt. | 2. Honor their decision (i.e., if don't want to know, don't discuss and vice versa). |
| | 3. Never tell patient they are dying. | 3. Be a part of their journey. |
| | 4. If family asks prognosis, give it in range only (never give date or time). | |
| | 5. Explain reasons for what's happening in the body very simply (and don't use any medical terms). | |
| | 6. Offer opportunity for patient and family to ask questions. If family doesn't understand, explain it differently. It's the physician's responsibility to make sure he/she is clear and helps patient/family to understand. | |
| | 7. If patient/family is religious (highly likely), physician can say, "I can see that you're a spiritual person, we're doing the best that we can and it's in God's hands." | |

© 2025 Bakitas MA et al. *JAMA Network Open*.

| | | |
|---|---|---|
| | 8. *<u>Always</u>* add that it's in God's hands/God decides. If physician not comfortable saying, "God", say, "it's in the hands of a higher power." | |
| | 9. If physician is comfortable, ask if you can pray with the patient/family. | |
| **a. Pt and family understanding of:** ii. goals of care, iii. Treatment preferences; iv. AD if available. | **Understand Goals of Care, Treatment Preferences & Advance Directives** | |
| | 1. When discussing Advance Care Directive, many patients/family confuse this with Power of Attorney, DNR and will. Ask what documents (if any) they have. | |
| | Recognize that Care instructions are given verbally to family member(s). There is very low likelihood of ACD but may have DNR and will. | 1. Ask if patient had any document of patient wishesin writing (don't specify which kind.) |
| | 1. If patient is unable to communicate: Ask if loved one shared instructions/directions of what they wanted for care with a family member. Ask who the family member is. | 2. Ask if they have been asked to complete any documents, which. If they have any questions about these, clarify very simply. |
| | 2. When palliative care doc speaks to family member, ask what the patient wanted in terms of care. | 3. If they have a written document, ask what these specified and ask if the patient has the same wishes or if they have changed, and how are these being followed in the hospital. |
| | | 4. If patient does not have AD, ask if they know what patient wanted in terms of care. |
| | | 5. If has no AD, ask if would like to complete one. |
| **Post-discharge plans** | **Understand Perceptions of Hospice** | |
| | *See also: Family Will Take Care of Loved One* | |

© 2025 Bakitas MA et al. *JAMA Network Open*.

| | | |
|---|---|---|
| | 1. Never mention the word hospice and don't raise the issue UNLESS the patient/caregiver raises the issue of hospice or expresses concern about burden of care OR asksabout hospice. | 1. Assess how patient and family feel about hospice but do not use the word, "hospice." Use "home health." |
| | 2. Ask which family members are helping to take care of patient at home and if they are, what kind of care they are providing. If it is the kind of care home hospice provides, explain that this is the type of care that home health provides. | 2. Whatever their response, acknowledge & respect their feelings/attitudes. |
| | 3. Ask if there are any specific concerns (e.g. cleaning a port, bathing a patient with an open wound) about the family providing care, and discuss until all concerns are alleviated. | 3. If open to it, talk about this is a helpful way to take care of the family at home. |
| | 4. Make sure to emphasize that they are NOT there to take over; the family is the one who decides what and how it is done. | 4. Make sure to emphasize that this is an offer of help and assistance. |
| | 5. If open to it, talk about this is a helpful way to take care of the family at home. | |
| | 6. Ask if they have any concerns about this kind of homehelp. If yes, discuss until concerns are alleviated. | |
| | 7. Whatever their response, acknowledge & respect their feelings/attitudes. | |
| | 8. If, following this discussion, patient/family wants home health/hospice, ask if they want you to make a recommendation for a referral to it. | |

© 2025 Bakitas MA et al. *JAMA Network Open*.

| | | |
|---|---|---|
| | 9. Stress that all decisions are up to the patient/family. The Palliative Care physician is here to help, NOT to change the way family takes care of loved one. | |
| | **Understand Perceptions of Nursing Homes** | |
| | 1. If patient is in the nursing home, or family/patient bringsit up, palliative care doc can discuss nursing home referral. If not, don't raise it. | If patient is in nursing home, help family deal with guilt about needing to place loved one in nursing home. |
| | 2. If loved one is going to nursing home, provide support to family. | |
| **c. Physical examination/ ASK about**: i. Identification of current symptoms; ii. Functional status | **Explanation of Medications** | |
| | 1. Explain why pain medication is needed, especially the administration of morphine and its dosing (and why it varies & more may be administered than family expect. | |
| | 2. If there is concern about lack of consciousness raised, explain balance between lack of pain and lack of consciousness. | |
| | 3. If concern about getting more morphine than was originally scheduled is raised, explain dose is flexible based on patient response. | |
| | 4. If concern about addiction is raised, explain that addiction is not an issue and why not. | |
| | 5. If fear of overdosing is raised (with potential to enhance death), address concern and ease fear. | |
| | 6. Explain clearly, simply in non-medical language. | |

© 2025 Bakitas MA et al. *JAMA Network Open*.

# eAppendix 2 Palliative Care Consultation Documentation Template

(for EMR integration)

**Hospital Name** **[AUTOPOPULATE, if possible]**

**Date of consult:** **[AUTOPOPULATE, if possible]**

**Patient Name/DOB/Gender/Race/MR#** **[AUTOPOPULATE, if possible]**

**Referral Source/Provider:** **[AUTOPOPULATE, if possible – if considered an "order"]**

**Reason for PC Consultation per referral source:**

**[If possible, have drop-down list with these options: (check all that apply)**

- **Symptom Management**
- **Support/Coping**
- **Goals of Care/Advance Care Planning**
- **Interdisciplinary referral(s)**
- **Local Resources / Community Care Medical / Other Support Communication**
- **Hospice / Home Services**
- **Other**

**History of Present Illness:**

**Past Medical/Surgical History:** **[AUTOPOPULATE, if possible]**

**Medications Review and Allergies:** **[AUTOPOPULATE, if possible]**

**Physical ROS/Cognition/Functional status:**

**Social History/Assessment:**

**Support Systems/Family concerns:**

**Spirituality/Beliefs:**

**Physical Exam-Limited:** **[AUTOPOPULATE Vital signs, Weight, Inputs/Outputs (including bowel movements) if possible]**

**Lab/Diagnostic studies/Records Review Highlights:** **[DO NOT AUTOPOPULATE]**

**Goals of Care / Advance Care Planning Assessment:**

**Global Assessment Statement:**

**Recommendations/Plan:**

**A. Symptom Management**

**B. Support / Coping**

**C. Goals of Care / Advance Care Planning (inc. AD, Proxy, DNR, POLST, etc.)**

**D. Interdisciplinary Referrals (PT, OT, Spiritual, Counselor, Social Work, etc.)**

**E. Local Resources / Community Care Medical / Other Support / Communication**

**F. Hospice / Home Services**

**G. Other**

**Transition/Discharge Plans (if known):**

© 2025 Bakitas MA et al. *JAMA Network Open*.

**eAppendix 3 Community Tele-Pal Videoconsultation Fidelity Monitoring Process**

The National Institutes of Health recommended that fidelity strategies be used to ensure reliability and validity of intervention delivery. Initial fidelity monitoring was completed after each clinician's first 3 consultations. Following that, monitoring of 10% of consultations was completed every 4 months.

1. All clinical research coordinators (CRCs) and study staff (CE, FU) underwent 30 hours of orientation and training overseen by the co-investigator (SG) and PI (MB) to conduct fidelity monitoring reviews on the Videoconsult protocol. This training included reviewing case studies to provide guidance on how to apply and properly score the Checklist, listening to videoconsultation recordings, and completing the REDCap Fidelity Monitoring Checklist (attached) based on the Videoconsultation protocol.
2. The Checklist was used to determine and record whether videoconsultation protocol concepts were present or omitted. Individually completed Checklist documents were compared to determine inter-rater agreement between team members.
3. To become grounded in the consultations, the first three recordings of each PC specialist's videoconsultation visits were reviewed and rated independently by two different team members. Following independent ratings, the checklists were compared, and discrepancies were discussed with the study PI (MB) and co-investigator (SG).
4. Videoconsultation fidelity monitoring standards were based on race (Black or White).For example, only individuals who identified as AA/Black noted the importance of inviting a religious leader to a meeting on prognosis; thus, for AA patients, this criterion would be marked Yes if it was present in the audio recording of the consult. However, for White patients, this criterion was marked nonapplicable (NA). Yes and NA were scored as "1". If a criterion that should be present was omitted, then the checklist item was marked as "0".

1. Calculating the Fidelity Score: To calculate the overall percentage of adherence, each Fidelity criterion (n=58) rated as Yes or NA, receive a score of one (1) and if the criterion that should have been present was not (No) then the score was zero (0). Hence, a total score of 58 was perfect or complete adherence to the protocol. Thus actual adherence scores were divided by the maximum score of 58 resulting in the adherence rate.

RESULTS:

2. Yearly, if gaps in protocol delivery were identified, all four clinicians were provided an update on areas for improvement.; If a clinician needed more direct reorientation to the protocol, PIs completed this task individually
3. A total of 9 (10%) of the 85 consults were reviewed for protocol fidelity per our pre-specified adherence monitoring goal. Scores ranged from 46 to 58. The average score of 52.4 resulting in a 90% adherence rate.

© 2025 Bakitas MA et al. *JAMA Network Open*.

# Fidelity Check (Culturally-Based PC Consult)

Please complete the Fidelity Checklist for the intervention patient below. You will listen to the digital audio recording saved in Box for this participant (Study ID: [a_screeningconsent_arm_1][record_id]) to complete the checklist.

**Fidelity Checklist for Culturally-Based Palliative Care Tele-Consult**

**Study ID: [a_screeningconsent_arm_1][record_id]**
**Palliative Care tele-consult date: [contact_1_pc_consu_arm_1][pc_consult_date]**

| | |
|---|---|
| During the Telehealth consult, the Palliative Care physician...wore a white coat. | ◯ Yes<br>◯ No<br>◯ Unable to determine<br>(Listen for this to be mentioned in the introduction, at the beginning of the recording.) |
| During the Telehealth consult, the Palliative Care physician...acknowledged telehealth. | ◯ Yes<br>◯ No<br>(Acknowledged that it's not the same as face-to-face.) |
| During the Telehealth consult, the Palliative Care physician...recognized/respected distrust. | ◯ Yes<br>◯ No |

**[Address Patient/Family, Establish Rapport, Family Care/Social History]**
**During the telehealth consult, the Palliative Care physician...**

| | Yes | No |
|---|---|---|
| Addressed patient by last name or title. | ◯ | ◯ |
| Made introductions in correct order: PC physician first, then PC physican asks pt/cg and all else in room to introduce themselves. Hospital staff and study coordinator last. | ◯ | ◯ |
| Began to establish rapport; got to know the patient. | ◯ | ◯ |
| Made additional time to get to know family. | ◯ | ◯ |
| Talked about something specific to the family. | ◯ | ◯ |
| Discussed something local. | ◯ | ◯ |
| Recognized that family will be there for the patient and care for them at home. Began with that assumption. | ◯ | ◯ |



© 2025 Bakitas MA et al. *JAMA Network Open*.

**[Illness Understanding/Prognosis Discussion]**
**During the Telehealth consult, the Palliative Care physician...**

| | Yes | No | Not applicable |
|---|---|---|---|
| Asked patient/family if they want to know prognosis. | ○ | ○ | ○ |
| Sensitively determined if patient/family wanted to know about prognosis. | ○ | ○ | ○ |
| Was not blunt during illness understanding/prognosis discussion. | ○ | ○ | ○ |
| Honored patient/family decision during illness understanding/prognosis discussion. | ○ | ○ | ○ |
| Never told the patient that they are dying. | ○ | ○ | ○ |
| PC physician was a part of their (patient/family's) journey. | ○ | ○ | ○ |
| Family asked about prognosis, Palliative Care physician gave it in a range (never a date or time). | ○ | ○ | ○ |
| Explained what's happening in body very simply (didn't use medical terms). | ○ | ○ | ○ |
| Offered opportunity for patient and family to ask questions. If family doesn't understand, explained in a different way. | ○ | ○ | ○ |
| If patient/family is religious, physician said "I can see that you're a spiritual person, we're doing the best that we can and it's in God's hands." | ○ | ○ | ○ |
| Physician said: "it's in God's hands/God decides." If physician not comfortable saying, "God," says, "it's in the hands of a higher power." | ○ | ○ | ○ |
| If physician comfortable, asked if he/she could pray with the patient/family. | ○ | ○ | ○ |



© 2025 Bakitas MA et al. *JAMA Network Open*.

**[Goals of Care, Treatment Preferences, & Advance Care Planning]**
**During the Telehealth consult, the Palliative Care physician...**

| | Yes | No | Not applicable |
|---|---|---|---|
| Recognized legal documents (i.e. an Advance Directive) are confusing. | ○ | ○ | ○ |
| Recognized care instructions given verbally to family. | ○ | ○ | ○ |
| Asked if patient has any documents of wishes (i.e. an Advance Directive). | ○ | ○ | ○ |
| Asked if patient has shared care instructions with family (and who). | ○ | ○ | ○ |
| Asked if patient has been asked to complete an Advance Directive. Clarified if any questions. | ○ | ○ | ○ |
| Asked family what care patient wants (or wanted). | ○ | ○ | ○ |
| If patient has an Advance Directive, PC physician asked: What it specified? Have they changed? Has hospital followed them? | ○ | ○ | ○ |
| If no Advance Directive, PC physician asked family if they know what care the patient wants. | ○ | ○ | ○ |
| If no Advance Directive, PC physician asked if patient wants to complete one. | ○ | ○ | ○ |

**[Role of Religion and Church]**
**During Telehealth consult, the Palliative Care physician...**

| | Yes | No | Not applicable |
|---|---|---|---|
| Asked about the patient's religious leader (pastor, minister, etc.). | ○ | ○ | ○ |
| Invited religious leader (pastor, etc.) to meeting on prognosis. | ○ | ○ | ○ |
| Recognized importance of religion: source of comfort, knowledge, a guide for all things. | ○ | ○ | ○ |



© 2025 Bakitas MA et al. *JAMA Network Open*.

| | | | |
|---|---|---|---|
| Recognized church members as source of support. | ○ | ○ | ○ |
| When support needed, asked if church member can assist; asked name of church member and discussed how they can provide support. | ○ | ○ | ○ |

| | |
|---|---|
| [Financial vulnerability] Recognized patient/family may experience substantial financial difficulties, with harsh/challenging realities. | ○ Yes<br>○ No<br>○ Not applicable |
| [Death and Dying] Approached possibility of death with great care/caution. | ○ Yes<br>○ No<br>○ Not applicable |

**[Home Services]**

**During the Telehealth consult, the Palliative Care physician...**

| | Yes | No | Not applicable |
|---|---|---|---|
| Didn't use 'hospice'; didn't raise it UNLESS the patient/caregiver raises it or expresses concern about burden of care OR asks about hospice. | ○ | ○ | ○ |
| Assessed how patient and family feel about hospice but do not use the word, "hospice." Use "home services." | ○ | ○ | ○ |
| Asked which family members are helping to take care of patient (and how). If it is the kind of care home health provides, explained that this is the type of care that home health provides. | ○ | ○ | ○ |
| Asked if there are any specific concerns (ex: cleaning a port, bathing a patient with an open wound) about family providing care. Discussed until all concerns alleviated. | ○ | ○ | ○ |
| If patient/family open to it [home services], talked about this is being a helpful way to take care of the family at home. | ○ | ○ | ○ |



© 2025 Bakitas MA et al. *JAMA Network Open*.

| | | | |
|---|---|---|---|
| Emphasized that home services NOT there to take over; the family is in charge. | ○ | ○ | ○ |
| Emphasized that this is an offer of help and assistance. | ○ | ○ | ○ |
| Asked if have any concerns about this kind of home help. Address concerns. | ○ | ○ | ○ |
| Acknowledged & respected their feelings/attitudes towards home services. | ○ | ○ | ○ |
| Patient/family wanted home services, PC physician asked if patient/family wanted him/her to make a recommendation for referral to it. | ○ | ○ | ○ |
| Stressed that all decisions are up to the patient/family. PC physician only there to help. | ○ | ○ | ○ |

**[Nursing Homes]**

**During the Telehealth consult, the Palliative Care physician...**

| | Yes | No | Not applicable |
|---|---|---|---|
| Only discussed nursing home referral if patient and/or family raised the issue. | ○ | ○ | ○ |
| If the patient is in a nursing home, helped family deal with guilt. | ○ | ○ | ○ |
| If loved one is going to nursing home, provided support to family. | ○ | ○ | ○ |

**[Medications/Symptoms]**

**During the Telehealth consult, the Palliative Care physician...**

| | Yes | No | Not applicable |
|---|---|---|---|
| Explained why pain medications needed, especially the administration of morphine and its dosing (and why it varies & more may be administered than the family expects). | ○ | ○ | ○ |



© 2025 Bakitas MA et al. *JAMA Network Open*.

| | | | |
|---|---|---|---|
| If concern about lack of consciousness raised: PC physician explained balance between lack of pain and lack of consciousness. | ○ | ○ | ○ |
| If concern about getting more morphine than was originally scheduled: PC physician explained dose is flexible based on patient response. | ○ | ○ | ○ |
| If concern about addiction is raised, PC physician explained that addiction is not an issue and why not. | ○ | ○ | ○ |
| If fear of overdosing is raised, PC physician addressed concern(s) and eased fear(s). | ○ | ○ | ○ |
| Explained clearly; simply in non-medical language. | ○ | ○ | ○ |

| | |
|---|---|
| Fidelity checklist completed by: | ○ AL CSC<br>○ AL BSC<br>○ AL Coordinator Other<br>○ MS CSC<br>○ MS BSC<br>○ MS Coordinator Other<br>○ SC CSC<br>○ SC BSC<br>○ SC Coordinator Other<br>○ Other study team member<br>(CSC=Coordinating Study Coordinator; BSC=Blinded Study Coordinator) |
| Other coordinator/study team member who completed fidelity checklist: | ______________________________<br>(Last Name, First Name) |
| Date fidelity checklist completed: | ______________________________ |



© 2025 Bakitas MA et al. *JAMA Network Open*.

**eTable 3:** **Characteristics of Participants Consented versus Declined**

| Characteristic | **Overall** N = 696[a] | Participants, No. (%) | | p-value[b] |
|---|---|---|---|---|
| | | **Consented** N = 209 | **Declined** N = 487 | |
| Age, mean (SD), years | 73.9 (9.1) | 73.3 (8.3) | 74.2 (9.4) | 0.3 |
| Gender | | | | 0.2 |
| Male | 320.0 (46.0%) | 89.0 (42.6%) | 231.0 (47.4%) | |
| Female | 376.0 (54.0%) | 120.0 (57.4%) | 256.0 (52.6%) | |
| Race | | | | 0.4 |
| White or Caucasian | 481.0 (69.1%) | 151.0 (72.2%) | 330.0 (67.8%) | |
| Black or African American | 212.0 (30.5%) | 58.0 (27.8%) | 154.0 (31.6%) | |
| Medical Conditon | | | | |
| Cancer | 205.0 (29.5%) | 60.0 (28.7%) | 145.0 (29.8%) | 0.8 |
| Cardiac disease | 573.0 (82.3%) | 169.0 (80.9%) | 404.0 (83.0%) | 0.5 |
| Pulmonary disease | 237.0 (34.1%) | 81.0 (38.8%) | 156.0 (32.0%) | 0.086 |
| Neurodegenerative disease | 33.0 (4.7%) | 11.0 (5.3%) | 22.0 (4.5%) | 0.7 |
| Renal disease | 313.0 (45.0%) | 100.0 (47.8%) | 213.0 (43.7%) | 0.3 |
| Stroke | 85.0 (12.2%) | 29.0 (13.9%) | 56.0 (11.5%) | 0.4 |
| Sepsis | 46.0 (6.6%) | 19.0 (9.1%) | 27.0 (5.5%) | 0.084 |
| Hepatic disease | 39.0 (5.6%) | 11.0 (5.3%) | 28.0 (5.7%) | 0.8 |
| Other | 100.0 (14.4%) | 26.0 (12.4%) | 74.0 (15.2%) | 0.3 |
| Cancer site | | | | 0.8 |
| Bladder | 13.0 (6.3%) | 4.0 (6.7%) | 9.0 (6.2%) | |
| Breast | 35.0 (17.1%) | 10.0 (16.7%) | 25.0 (17.2%) | |
| Cervix | 1.0 (0.5%) | 0.0 (0.0%) | 1.0 (0.7%) | |
| Colorectal | 22.0 (10.7%) | 9.0 (15.0%) | 13.0 (9.0%) | |
| Esophagus | 1.0 (0.5%) | 0.0 (0.0%) | 1.0 (0.7%) | |
| Head & neck (mouth, nose, throat, etc.) | 4.0 (2.0%) | 1.0 (1.7%) | 3.0 (2.1%) | |
| Kidney (renal) | 14.0 (6.8%) | 5.0 (8.3%) | 9.0 (6.2%) | |
| Liver (hepatic) | 4.0 (2.0%) | 1.0 (1.7%) | 3.0 (2.1%) | |
| Lung | 21.0 (10.2%) | 6.0 (10.0%) | 15.0 (10.3%) | |
| Ovary | 2.0 (1.0%) | 0.0 (0.0%) | 2.0 (1.4%) | |
| Pancreas | 8.0 (3.9%) | 4.0 (6.7%) | 4.0 (2.8%) | |
| Prostate | 40.0 (19.5%) | 7.0 (11.7%) | 33.0 (22.8%) | |
| Stomach | 2.0 (1.0%) | 0.0 (0.0%) | 2.0 (1.4%) | |
| Other | 38.0 (18.5%) | 13.0 (21.7%) | 25.0 (17.2%) | |
| CAB contact | 579.0 (83.4%) | 176.0 (84.2%) | 403.0 (83.1%) | 0.7 |
| Missing | 2 | 0 | 2 | |
| CAB contact method | | | | <0.001 |
| In-person | 395.0 (68.2%) | 77.0 (43.8%) | 318.0 (78.9%) | |
| Zoom | 7.0 (1.2%) | 1.0 (0.6%) | 6.0 (1.5%) | |
| Pre-recorded video | 177.0 (30.6%) | 98.0 (55.7%) | 79.0 (19.6%) | |
| Missing | 117 | 33 | 84 | |

© 2025 Bakitas MA et al. *JAMA Network Open*.

**eTable 3:** **Characteristics of Participants Consented versus Declined**

| | | Participants, No. (%) | | |
|---|---|---|---|---|
| **Characteristic** | **Overall** N = 696[a] | **Consented** N = 209 | **Declined** N = 487 | **p-value**[b] |

Abbreviations: CAB, Community advisory board; NA, not applicable; SIS, Six Item Screener.

[a]Mean (SD); n (%)

[b]Wilcoxon rank sum test; Pearson's Chi-squared test; Fisher's Exact Test for Count Data with simulated p-value (based on 2000 replicates)

© 2025 Bakitas MA et al. *JAMA Network Open*.

| **eTable4 Caregiver Baseline Demographics and Outcomes** | **N (%)** | | |
|---|---|---|---|
| **Characteristic** | **Intervention**, N = 105 | **Usual Care**, N = 104 | **Effect Size**[a] |
| | | | |
| **Age** | 61.5 (14.6) | 58.6 (15.5) | 0.19 |
| **Gender** | | | 0.11 |
| Male | 22.0 (21.0%) | 29.0 (27.9%) | |
| Female | 83.0 (79.0%) | 72.0 (69.2%) | |
| *Missing or no response* | *0.0 (0.0%)* | *3.0 (2.9%)* | |
| **Race** | | | 0.10 |
| White or Caucasian | 76.0 (72.4%) | 73.0 (70.2%) | |
| Black or African American | 29.0 (27.6%) | 27.0 (26.0%) | |
| *Missing or no response* | *0.0 (0.0%)* | *4.0 (3.8%)* | |
| **Marital status** | | | 0.00 |
| Married or living with partner | 74.0 (70.5%) | 70.0 (67.3%) | |
| Never married | 14.0 (13.3%) | 13.0 (12.5%) | |
| Divorced or separated | 12.0 (11.4%) | 14.0 (13.5%) | |
| Widowed | 5.0 (4.8%) | 4.0 (3.8%) | |
| *Missing or no response* | *0.0 (0.0%)* | *3.0 (2.9%)* | |
| **Education** | | | 0.00 |
| Less than high school graduate | 8.0 (7.6%) | 15.0 (14.4%) | |
| High school graduate or GED | 34.0 (32.4%) | 29.0 (27.9%) | |
| Some college or technical school | 30.0 (28.6%) | 29.0 (27.9%) | |
| College graduate or above | 32.0 (30.5%) | 28.0 (26.9%) | |
| *Missing or no response* | *1.0 (1.0%)* | *3.0 (2.9%)* | |
| **Employment** | | | 0.11 |
| Retired | 46.0 (43.8%) | 37.0 (35.6%) | |
| Full or part time | 41.0 (39.0%) | 36.0 (34.6%) | |
| Not employed | 17.0 (16.2%) | 27.0 (26.0%) | |
| *Missing or no response* | *1.0 (1.0%)* | *4.0 (3.8%)* | |
| **Relationship** | | | 0.16 |
| Spouse/Partner | 57.0 (54.3%) | 39.0 (37.5%) | |
| Parent | 1.0 (1.0%) | 2.0 (1.9%) | |
| Other | 47.0 (44.8%) | 60.0 (57.7%) | |
| *Missing or no response* | *0.0 (0.0%)* | *3.0 (2.9%)* | |
| **Lives with patient** | | | 0.09 |
| Yes | 74.0 (70.5%) | 61.0 (58.7%) | |
| No | 30.0 (28.6%) | 40.0 (38.5%) | |
| Missing or no response | 1.0 (1.0%) | 3.0 (2.9%) | |

© 2025 Bakitas MA et al. *JAMA Network Open*.

| eTable4 Caregiver Baseline Demographics and Outcomes | N (%) | | |
|---|---|---|---|
| **Characteristic** | **Intervention**, N = 105 | **Usual Care**, N = 104 | **Effect Size**[a] |
| **Caregiver-reported outcomes, mean (SD)** | | | |
| Primary | | | |
| Montgomery-Borgatta Caregiver Burden Scale | | | |
| MBCB Objective Burden[c] | 19.5 (3.8) | 19.9 (3.2) | -0.09 |
| *Missing* | *2* | *7* | |
| MBCB Subjective Burden[d] | 13.4 (2.4) | 13.2 (3.1) | 0.09 |
| *Missing* | *2* | *6* | |
| MBCB Demand Burden[e] | 12.1 (2.1) | 11.7 (2.5) | 0.18 |
| *Missing* | *9* | *16* | |
| Secondary | | | |
| PROMIS Global[b] | | | |
| PROMIS Physical Health T Score | 48.5 (8.4) | 47.6 (9.7) | 0.10 |
| *Missing* | *1* | *3* | |
| PROMIS Mental Health T Score | 50.0 (6.4) | 47.7 (8.2) | 0.32 |
| *Missing* | *3* | *3* | |
| FAMCARE | | | |
| Patients comfort | | | 0.08 |
| Very satisfied | 30.0 (28.8%) | 16.0 (15.8%) | |
| Satisfied | 47.0 (45.2%) | 61.0 (60.4%) | |
| Undecided | 11.0 (10.6%) | 11.0 (10.9%) | |
| Dissatisfied | 12.0 (11.5%) | 9.0 (8.9%) | |
| Very dissatisfied | 1.0 (1.0%) | 1.0 (1.0%) | |
| Not applicable | 3.0 (2.9%) | 3.0 (3.0%) | |
| *Missing* | *1* | *3* | |
| Condition and progress explained | | | 0.00 |
| Very satisfied | 21.0 (20.2%) | 15.0 (14.9%) | |
| Satisfied | 46.0 (44.2%) | 48.0 (47.5%) | |
| Undecided | 17.0 (16.3%) | 12.0 (11.9%) | |
| Dissatisfied | 11.0 (10.6%) | 15.0 (14.9%) | |
| Very dissatisfied | 3.0 (2.9%) | 1.0 (1.0%) | |
| Not applicable | 6.0 (5.8%) | 10.0 (9.9%) | |
| *Missing* | *1* | *3* | |
| Information on side effects | | | 0.00 |
| Very satisfied | 17.0 (16.3%) | 10.0 (9.9%) | |
| Satisfied | 57.0 (54.8%) | 55.0 (54.5%) | |
| Undecided | 11.0 (10.6%) | 15.0 (14.9%) | |
| Dissatisfied | 12.0 (11.5%) | 11.0 (10.9%) | |

© 2025 Bakitas MA et al. *JAMA Network Open*.

| eTable4 Caregiver Baseline Demographics and Outcomes | N (%) | | |
|---|---|---|---|
| **Characteristic** | **Intervention**, N = 105 | **Usual Care**, N = 104 | **Effect Size**[a] |
| Very dissatisfied | 2.0 (1.9%) | 2.0 (2.0%) | |
| Not applicable | 5.0 (4.8%) | 8.0 (7.9%) | |
| *Missing* | *1* | *3* | |
| Respect for dignity | | | 0.00 |
| Very satisfied | 35.0 (33.7%) | 22.0 (21.8%) | |
| Satisfied | 54.0 (51.9%) | 61.0 (60.4%) | |
| Undecided | 7.0 (6.7%) | 8.0 (7.9%) | |
| Dissatisfied | 4.0 (3.8%) | 3.0 (3.0%) | |
| Very dissatisfied | 1.0 (1.0%) | 1.0 (1.0%) | |
| Not applicable | 3.0 (2.9%) | 6.0 (5.9%) | |
| *Missing* | *1* | *3* | |
| Meetings with care team | | | 0.00 |
| Very satisfied | 13.0 (12.7%) | 16.0 (15.8%) | |
| Satisfied | 43.0 (42.2%) | 42.0 (41.6%) | |
| Undecided | 17.0 (16.7%) | 12.0 (11.9%) | |
| Dissatisfied | 15.0 (14.7%) | 14.0 (13.9%) | |
| Very dissatisfied | 1.0 (1.0%) | 3.0 (3.0%) | |
| Not applicable | 13.0 (12.7%) | 14.0 (13.9%) | |
| *Missing* | *3* | *3* | |
| Speed of treated | | | 0.00 |
| Very satisfied | 21.0 (20.2%) | 15.0 (14.9%) | |
| Satisfied | 49.0 (47.1%) | 45.0 (44.6%) | |
| Undecided | 11.0 (10.6%) | 19.0 (18.8%) | |
| Dissatisfied | 16.0 (15.4%) | 15.0 (14.9%) | |
| Very dissatisfied | 5.0 (4.8%) | 4.0 (4.0%) | |
| Not applicable | 2.0 (1.9%) | 3.0 (3.0%) | |
| *Missing* | *1* | *3* | |
| Attention to symptom description | | | 0.16 |
| Very satisfied | 29.0 (27.9%) | 12.0 (11.9%) | |
| Satisfied | 50.0 (48.1%) | 57.0 (56.4%) | |
| Undecided | 8.0 (7.7%) | 11.0 (10.9%) | |
| Dissatisfied | 12.0 (11.5%) | 10.0 (9.9%) | |
| Very dissatisfied | 3.0 (2.9%) | 6.0 (5.9%) | |
| Not applicable | 2.0 (1.9%) | 5.0 (5.0%) | |
| *Missing* | *1* | *3* | |
| Physical needs met | | | 0.06 |
| Very satisfied | 24.0 (23.1%) | 14.0 (13.9%) | |

© 2025 Bakitas MA et al. *JAMA Network Open*.

| eTable4 Caregiver Baseline Demographics and Outcomes | N (%) | | |
|---|---|---|---|
| **Characteristic** | **Intervention**, N = 105 | **Usual Care**, N = 104 | **Effect Size**[a] |
| Satisfied | 59.0 (56.7%) | 64.0 (63.4%) | |
| Undecided | 7.0 (6.7%) | 12.0 (11.9%) | |
| Dissatisfied | 10.0 (9.6%) | 7.0 (6.9%) | |
| Very dissatisfied | 1.0 (1.0%) | 0.0 (0.0%) | |
| Not applicable | 3.0 (2.9%) | 4.0 (4.0%) | |
| *Missing* | *1* | *3* | |
| Availability of care team | | | 0.00 |
| Very satisfied | 20.0 (19.2%) | 19.0 (18.8%) | |
| Satisfied | 53.0 (51.0%) | 49.0 (48.5%) | |
| Undecided | 12.0 (11.5%) | 8.0 (7.9%) | |
| Dissatisfied | 12.0 (11.5%) | 18.0 (17.8%) | |
| Very dissatisfied | 3.0 (2.9%) | 1.0 (1.0%) | |
| Not applicable | 4.0 (3.8%) | 6.0 (5.9%) | |
| *Missing* | *1* | *3* | |
| Emotional support to family | | | 0.00 |
| Very satisfied | 18.0 (17.3%) | 14.0 (13.9%) | |
| Satisfied | 48.0 (46.2%) | 53.0 (52.5%) | |
| Undecided | 10.0 (9.6%) | 5.0 (5.0%) | |
| Dissatisfied | 9.0 (8.7%) | 13.0 (12.9%) | |
| Very dissatisfied | 4.0 (3.8%) | 2.0 (2.0%) | |
| Not applicable | 15.0 (14.4%) | 14.0 (13.9%) | |
| *Missing* | *1* | *3* | |
| Practical assistance | | | 0.08 |
| Very satisfied | 17.0 (16.3%) | 16.0 (15.8%) | |
| Satisfied | 39.0 (37.5%) | 44.0 (43.6%) | |
| Undecided | 5.0 (4.8%) | 7.0 (6.9%) | |
| Dissatisfied | 3.0 (2.9%) | 4.0 (4.0%) | |
| Very dissatisfied | 1.0 (1.0%) | 5.0 (5.0%) | |
| Not applicable | 39.0 (37.5%) | 25.0 (24.8%) | |
| *Missing* | *1* | *3* | |
| Doctors attention to symptoms | | | 0.07 |
| Very satisfied | 28.0 (26.9%) | 16.0 (15.8%) | |
| Satisfied | 53.0 (51.0%) | 59.0 (58.4%) | |
| Undecided | 6.0 (5.8%) | 6.0 (5.9%) | |
| Dissatisfied | 11.0 (10.6%) | 10.0 (9.9%) | |
| Very dissatisfied | 3.0 (2.9%) | 2.0 (2.0%) | |
| Not applicable | 3.0 (2.9%) | 8.0 (7.9%) | |

© 2025 Bakitas MA et al. *JAMA Network Open*.

| eTable4 Caregiver Baseline Demographics and Outcomes | N (%) | | |
|---|---|---|---|
| **Characteristic** | **Intervention**, N = 105 | **Usual Care**, N = 104 | **Effect Size**[a] |
| *Missing* | *1* | *3* | |
| Family is included | | | 0.19 |
| Very satisfied | 30.0 (28.8%) | 15.0 (14.9%) | |
| Satisfied | 52.0 (50.0%) | 53.0 (52.5%) | |
| Undecided | 10.0 (9.6%) | 6.0 (5.9%) | |
| Dissatisfied | 8.0 (7.7%) | 13.0 (12.9%) | |
| Very dissatisfied | 1.0 (1.0%) | 3.0 (3.0%) | |
| Not applicable | 3.0 (2.9%) | 11.0 (10.9%) | |
| *Missing* | *1* | *3* | |
| Information about managing symptoms | | | 0.00 |
| Very satisfied | 12.0 (11.5%) | 14.0 (14.0%) | |
| Satisfied | 53.0 (51.0%) | 50.0 (50.0%) | |
| Undecided | 15.0 (14.4%) | 9.0 (9.0%) | |
| Dissatisfied | 10.0 (9.6%) | 12.0 (12.0%) | |
| Very dissatisfied | 1.0 (1.0%) | 3.0 (3.0%) | |
| Not applicable | 13.0 (12.5%) | 12.0 (12.0%) | |
| *Missing* | *1* | *4* | |
| Effectiveness of care team | | | 0.00 |
| Very satisfied | 23.0 (22.1%) | 13.0 (12.9%) | |
| Satisfied | 55.0 (52.9%) | 60.0 (59.4%) | |
| Undecided | 9.0 (8.7%) | 11.0 (10.9%) | |
| Dissatisfied | 11.0 (10.6%) | 10.0 (9.9%) | |
| Very dissatisfied | 2.0 (1.9%) | 1.0 (1.0%) | |
| Not applicable | 4.0 (3.8%) | 6.0 (5.9%) | |
| *Missing* | *1* | *3* | |
| Response to changes | | | 0.00 |
| Very satisfied | 18.0 (17.3%) | 16.0 (15.8%) | |
| Satisfied | 59.0 (56.7%) | 57.0 (56.4%) | |
| Undecided | 12.0 (11.5%) | 9.0 (8.9%) | |
| Dissatisfied | 9.0 (8.7%) | 12.0 (11.9%) | |
| Very dissatisfied | 2.0 (1.9%) | 0.0 (0.0%) | |
| Not applicable | 4.0 (3.8%) | 7.0 (6.9%) | |
| *Missing* | *1* | *3* | |
| Emotional support to patient | | | 0.07 |
| Very satisfied | 22.0 (21.2%) | 13.0 (13.0%) | |
| Satisfied | 57.0 (54.8%) | 59.0 (59.0%) | |
| Undecided | 9.0 (8.7%) | 10.0 (10.0%) | |

© 2025 Bakitas MA et al. *JAMA Network Open*.

| eTable4 Caregiver Baseline Demographics and Outcomes | N (%) | | |
|---|---|---|---|
| **Characteristic** | **Intervention**, N = 105 | **Usual Care**, N = 104 | **Effect Size**[a] |
| Dissatisfied | 9.0 (8.7%) | 8.0 (8.0%) | |
| Very dissatisfied | 2.0 (1.9%) | 0.0 (0.0%) | |
| Not applicable | 5.0 (4.8%) | 10.0 (10.0%) | |
| *Missing* | *1* | *4* | |
| Montgomery-Borgatta Caregiver Burden Scale | | | |
| MBCB Objective Burden[c] | 19.5 (3.8) | 19.9 (3.2) | -0.09 |
| *Missing* | *2* | *7* | |
| MBCB Subjective Burden[d] | 13.4 (2.4) | 13.2 (3.1) | 0.09 |
| *Missing* | *2* | *6* | |
| MBCB Demand Burden[e] | 12.1 (2.1) | 11.7 (2.5) | 0.18 |
| *Missing* | *9* | *16* | |
| Heard and understood | | | 0.11 |
| Completely | 43.0 (41.7%) | 37.0 (36.6%) | |
| Quite a bit | 30.0 (29.1%) | 19.0 (18.8%) | |
| Moderately | 7.0 (6.8%) | 13.0 (12.9%) | |
| Slightly | 10.0 (9.7%) | 9.0 (8.9%) | |
| Not at all | 4.0 (3.9%) | 8.0 (7.9%) | |
| Not applicable (ex: no communication w/ doctor/nurse/hospital staff) | 9.0 (8.7%) | 15.0 (14.9%) | |
| *Missing* | *2* | *3* | |

Abbreviations: MBCB, Montgomery-Borgatta Caregiver Burden Scale; NA, not applicable; PROMIS, Patient-Reported Outcomes Measurement System.

[a]For continuous variables, effect size is Cohen's d (between-group mean difference divided by pooled standard deviation), with 0.2 being a small effect size, 0.5 medium effect size, and 0.8 large effect size. For categorical variables, effect size is Cramer's v, with less than 0.2 being a weak association, 0.2 to 0.6 moderate association, and greater than 0.6 strong association.

[b]The PROMIS Global Physical and Mental health mean (SD) score is 50 (10); higher scores indicate better health.

[c]The MBCB Objective score ranges from 6 to 30; higher scores indicate greater burden.

[d]The MBCB Subjective score ranges from 4 to 20; higher scores indicate greater burden.

[e]The MBCB Demand score ranges from 4 to 20; higher scores indicate greater burden.

© 2025 Bakitas MA et al. *JAMA Network Open*.

| eTable 5 Comparison of Demographics of Intervention Patients who Completed and Did not Complete Videoconsultation | | | | | |
|---|---|---|---|---|---|
| | | Participants, No. (%) | | | |
| **Characteristic** | **Overall N=105[a]** | **Completed** (n = 85) | **Did Not Complete** (n = 20) | **Effect Size[b]** | **P Value[c]** |
| **Age, mean (SD), years** | 72.9 (8.7) | 73.2 (8.7) | 71.4 (8.9) | 0.21 | 0.3 |
| **Gender** | | | | 0.00 | 0.7 |
| Male | 52.0 (49.5%) | 43.0 (50.6%) | 9.0 (45.0%) | | |
| Female | 53.0 (50.5%) | 42.0 (49.4%) | 11.0 (55.0%) | | |
| **Race** | | | | 0.00 | 0.4 |
| White or Caucasian | 75.0 (71.4%) | 59.0 (69.4%) | 16.0 (80.0%) | | |
| Black or African American | 30.0 (28.6%) | 26.0 (30.6%) | 4.0 (20.0%) | | |
| **Marital Status** | | | | 0.16 | 0.2 |
| Married | 60.0 (57.1%) | 50.0 (58.8%) | 10.0 (50.0%) | | |
| Widowed | 24.0 (22.9%) | 21.0 (24.7%) | 3.0 (15.0%) | | |
| Divorced | 11.0 (10.5%) | 7.0 (8.2%) | 4.0 (20.0%) | | |
| Never married | 6.0 (5.7%) | 5.0 (5.9%) | 1.0 (5.0%) | | |
| Living with a partner | 3.0 (2.9%) | 1.0 (1.2%) | 2.0 (10.0%) | | |
| Separated | 1.0 (1.0%) | 1.0 (1.2%) | 0.0 (0.0%) | | |
| **Education** | | | | 0.05 | 0.5 |
| 8th grade or less | 2.0 (1.9%) | 2.0 (2.4%) | 0.0 (0.0%) | | |
| Some high school | 29.0 (27.6%) | 20.0 (23.5%) | 9.0 (45.0%) | | |
| High school graduate or GED | 30.0 (28.6%) | 25.0 (29.4%) | 5.0 (25.0%) | | |
| Some college or technical school | 25.0 (23.8%) | 22.0 (25.9%) | 3.0 (15.0%) | | |
| College graduate | 14.0 (13.3%) | 11.0 (12.9%) | 3.0 (15.0%) | | |
| Graduate degree | 5.0 (4.8%) | 5.0 (5.9%) | 0.0 (0.0%) | | |
| **Employment** | | | | 0.14 | 0.3 |
| Retired | 74.0 (70.5%) | 61.0 (71.8%) | 13.0 (65.0%) | | |
| Unemployed (disability) | 18.0 (17.1%) | 14.0 (16.5%) | 4.0 (20.0%) | | |
| Full time | 7.0 (6.7%) | 5.0 (5.9%) | 2.0 (10.0%) | | |
| Part time | 5.0 (4.8%) | 5.0 (5.9%) | 0.0 (0.0%) | | |
| Homemaker | 1.0 (1.0%) | 0.0 (0.0%) | 1.0 (5.0%) | | |
| Other | 0.0 (0.0%) | 0.0 (0.0%) | 0.0 (0.0%) | | |
| **Religious preference** | | | | 0.00 | 0.6 |
| Protestant | 87.0 (82.9%) | 69.0 (81.2%) | 18.0 (90.0%) | | |
| Catholic | 6.0 (5.7%) | 6.0 (7.1%) | 0.0 (0.0%) | | |
| Jewish | 1.0 (1.0%) | 1.0 (1.2%) | 0.0 (0.0%) | | |
| Other | 7.0 (6.7%) | 5.0 (5.9%) | 2.0 (10.0%) | | |
| None | 4.0 (3.8%) | 4.0 (4.7%) | 0.0 (0.0%) | | |
| **Charlson Comorbidity Index Score** | 4.6 (2.7) | 4.4 (2.8) | 5.4 (2.5) | -0.33 | 0.091 |
| **Patient-reported outcomes, mean (SD)** | | | | | |
| Primary | | | | | |
| ESAS Total Distress[d] | 36.2 (16.8) | 36.8 (17.3) | 33.9 (14.7) | 0.17 | 0.5 |
| Missing | 2 | 2 | 0 | | |
| Secondary | | | | | |

© 2025 Bakitas MA et al. *JAMA Network Open*.

**eTable 5 Comparison of Demographics of Intervention Patients who Completed and Did not Complete Videoconsultation**

| Characteristic | Participants, No. (%) | | | | |
|---|---|---|---|---|---|
| | **Overall N=105**[a] | **Completed** (n = 85) | **Did Not Complete** (n = 20) | **Effect Size**[b] | **P Value**[c] |
| PROMIS Global[e] | | | | | |
| Physical health T score | 35.1 (8.0) | 34.5 (7.7) | 37.8 (8.9) | -0.42 | 0.14 |
| Mental health T score | 45.5 (7.7) | 45.2 (7.0) | 46.9 (10.0) | -0.22 | 0.8 |
| Missing | 1 | 1 | 0 | | |
| Exploratory | | | | | |
| Heard and understood | | | | 0.24 | 0.10 |
| Completely | 54.0 (51.4%) | 46.0 (54.1%) | 8.0 (40.0%) | | |
| Quite a bit | 26.0 (24.8%) | 19.0 (22.4%) | 7.0 (35.0%) | | |
| Moderately | 15.0 (14.3%) | 13.0 (15.3%) | 2.0 (10.0%) | | |
| Slightly | 7.0 (6.7%) | 6.0 (7.1%) | 1.0 (5.0%) | | |
| Not at all | 2.0 (1.9%) | 0.0 (0.0%) | 2.0 (10.0%) | | |
| Not applicable (ex: no communication w/ doctors/nurses/hospital staff) | 1.0 (0.5%) | 1.0 (1.0%) | 1.0 (1.2%) | | |
| Secondary, resource use, mean (SD) | | | | | |
| Emergency department visits, last 30 days | | | | 0.17 | 0.14 |
| 0 | 78.0 (88.6%) | 64.0 (90.1%) | 14.0 (82.4%) | | |
| 1 | 6.0 (6.8%) | 3.0 (4.2%) | 3.0 (17.6%) | | |
| 2 or more | 4.0 (4.5%) | 4.0 (5.6%) | 0.0 (0.0%) | | |
| Missing | 17 | 14 | 3 | | |
| Hospital readmissions, last 30 days | | | | 0.20 | 0.071 |
| 0 | 78.0 (86.7%) | 64.0 (88.9%) | 14.0 (77.8%) | | |
| 1 | 8.0 (8.9%) | 4.0 (5.6%) | 4.0 (22.2%) | | |
| 2 or more | 4.0 (4.4%) | 4.0 (5.6%) | 0.0 (0.0%) | | |
| Missing | 15 | 13 | 2 | | |

Abbreviations: ESAS, Edmonton Symptom Assessment System; GED, General Educational Development test; NA, not applicable; PROMIS, Patient-Reported Outcomes Measurement System.

[a]For continuous variables, effect size is Cohen's d (between-group mean difference divided by pooled standard deviation), with 0.2 being a small effect size, 0.5 medium effect size, and 0.8 large effect size. For categorical variables, effect size is Cramer's v, with less than 0.2 being a weak association, 0.2 to 0.6 moderate association, and greater than 0.6 strong association.

[b]The ESAS score ranges from 0 to 90; higher scores indicate worse symptoms.

[c]The PROMIS Global Physical and Mental health mean (SD) score is 50 (10); higher scores indicate better health.

© 2025 Bakitas MA et al. *JAMA Network Open*.

**eTable 6 Patient Satisfaction With The Videoconsultation Technology And Process**

| Telehealth Technology Acceptance Survey (First Contact) Technology Acceptance (N=85) | Strongly Agree | | Agree | | Somewhat Agree | | Somewhat Disagree | | Disagree | | Strongly Disagree | | Don't Know/Refuse | |
|---|---|---|---|---|---|---|---|---|---|---|---|---|---|---|
| | n | % | n | % | n | % | n | % | n | % | n | % | n | % |
| I liked being able to talk to the palliative care provider on a video call. | 38 | 44.7% | 45 | 52.9% | 2 | 2.4% | 0 | 0.0% | 0 | 0.0% | 0 | 0.0% | 0 | 0.0% |
| I am comfortable talking to my care provider on a video call in the future. | 39 | 45.9% | 41 | 48.2% | 2 | 2.4% | 2 | 2.4% | 0 | 0.0% | 1 | 1.2% | 0 | 0.0% |
| I could easily talk to the care provider using the telehealth system (cart or iPad). | 37 | 43.5% | 39 | 45.9% | 7 | 8.2% | 1 | 1.2% | 1 | 1.2% | 0 | 0.0% | 0 | 0.0% |
| I could hear the care provider clearly using the telehealth system (cart or iPad). | 41 | 48.2% | 37 | 43.5% | 5 | 5.9% | 1 | 1.2% | 1 | 1.2% | 0 | 0.0% | 0 | 0.0% |
| I felt I was able to express myself effectively | 37 | 43.5% | 42 | 49.4% | 6 | 7.1% | 0 | 0.0% | 0 | 0.0% | 0 | 0.0% | 0 | 0.0% |
| Using the telehealth system, I could see the clinician as well as if we met in person | 34 | 40.0% | 39 | 45.9% | 7 | 8.2% | 0 | 0.0% | 2 | 2.4% | 0 | 0.0% | 1 | 1.2% |
| I felt comfortable communicating with the palliative care provider using the telehealth system | 39 | 45.9% | 41 | 48.2% | 4 | 4.7% | 1 | 1.2% | 0 | 0.0% | 0 | 0.0% | 0 | 0.0% |
| Telehealth is an acceptable way to receive healthcare services | 31 | 36.5% | 47 | 55.3% | 5 | 5.9% | 0 | 0.0% | 2 | 2.4% | 0 | 0.0% | 0 | 0.0% |
| I would use telehealth services again | 34 | 40.0% | 44 | 51.8% | 5 | 5.9% | 1 | 1.2% | 1 | 1.2% | 0 | 0.0% | 0 | 0.0% |
| Overall, I am satisfied with this telehealth program | 38 | 44.7% | 45 | 52.9% | 1 | 1.2% | 0 | 0.0% | 1 | 1.2% | 0 | 0.0% | 0 | 0.0% |

© 2025 Bakitas MA et al. *JAMA Network Open*.

**Primary Outcome – Sensitivity Analysis***

**eTable 7: ESAS Total Symptom Distress (Intervention vs Usual Care)- Adjusted for Baseline**

| | Videoconsultation | | Usual Care | | Between-group difference in scores[a] | | |
|---|---|---|---|---|---|---|---|
| Outcome, No. of days after baseline | Participants, No. | Score, mean (SE) | Participants, No. | Score, mean (SE) | Mean (SE) | Effect size | P value |
| ESAS Total Distress[b] | | | | | | | |
| 7 | 94 | 24.5 (1.3) | 96 | 28.5 (1.3) | -4 (1.8) | 0.326 (0.035 to 0.616) | 0.02 |
| 30 | 87 | 20.5 (1.3) | 91 | 23.9 (1.3) | -3.4 (1.9) | 0.272 (-0.027 to 0.571) | 0.07 |

Abbreviations: ESAS, Edmonton Symptom Assessment System; NA, not applicable.

[a]Intervention minus usual care group; change between groups was calculated as least square mean difference for follow-up (days 7 and 30) adjusted for baseline; P values are from the time by group interaction term in mixed models; effect size was calculated as Westfall's d for the time by group interaction, with 0.2 being a small effect size, 0.5 medium effect size, and 0.8 large effect size.

[b]The ESAS score ranges from 0 to 90, with higher scores indicating worse symptoms.

*We also performed a sensitivity analysis that compared the estimated marginal means between videoconsultation and usual care groups at day 7 and 30, adjusting for baseline values as a covariate, which is an alternative method of formulating the mixed model.

© 2025 Bakitas MA et al. *JAMA Network Open*.

## eTable 8. Caregiver Burden, QOL, and FAMCARE From Baseline to 30 Days (Intervention vs Usual Care)

| | Intervention | | | Usual Care | | | Between-group difference in change from baseline[a] | | |
|---|---|---|---|---|---|---|---|---|---|
| Outcome, No. of days after baseline | Participants, No. | Score, mean (SE) | Change from baseline, mean (SE) | Participants, No. | Score, mean (SE) | Change from baseline, mean (SE) | Mean (SE) | Effect size | P value |
| **Montgomery-Borgatta Caregiver Burden Scale** | | | | | | | | | |
| Objective | | | | | | | | | |
| 0 | 103 | 19.5 (0.3) | NA | 97 | 19.9 (0.3) | NA | NA | NA | NA |
| 7 | 88 | 19.1 (0.3) | -0.5 (0.4) | 93 | 19.4 (0.3) | -0.5 (0.4) | 0 (0.5) | 0 | 0.98 |
| 30 | 86 | 19.4 (0.3) | -0.1 (0.4) | 91 | 19.1 (0.3) | -0.8 (0.4) | 0.7 (0.5) | -0.13 | 0.21 |
| Subjective | | | | | | | | | |
| 0 | 103 | 13.4 (0.3) | NA | 98 | 13.1 (0.3) | NA | NA | NA | NA |
| 7 | 78 | 11.9 (0.3) | -1.5 (0.3) | 85 | 11.6 (0.3) | -1.5 (0.3) | 0 (0.5) | 0 | 0.97 |
| 30 | 72 | 11.8 (0.3) | -1.6 (0.3) | 87 | 12.2 (0.3) | -0.9 (0.3) | -0.7 (0.5) | 0.16 | 0.14 |
| Demand | | | | | | | | | |
| 0 | 96 | 12.1 (0.3) | NA | 88 | 11.8 (0.3) | NA | NA | NA | NA |
| 7 | 72 | 11 (0.3) | -1.2 (0.3) | 78 | 10.8 (0.3) | -1 (0.3) | -0.2 (0.4) | 0.05 | 0.65 |
| 30 | 68 | 11.3 (0.3) | -0.8 (0.3) | 79 | 11.3 (0.3) | -0.5 (0.3) | -0.3 (0.4) | 0.08 | 0.46 |
| **PROMIS global physical health T-score** | | | | | | | | | |
| 0 | 104 | 48.5 (0.9) | NA | 101 | 47.6 (0.9) | NA | NA | NA | NA |
| 7 | 94 | 50.9 (0.9) | 2.4 (0.8) | 95 | 49.4 (0.9) | 1.8 (0.8) | 0.5 (1.2) | -0.05 | 0.66 |
| 30 | 90 | 50.5 (0.9) | 1.9 (0.8) | 89 | 49.4 (0.9) | 1.8 (0.9) | 0.1 (1.2) | -0.01 | 0.91 |
| **PROMIS global mental health T-score[b]** | | | | | | | | | |
| 0 | 102 | 49.2 (0.5) | NA | 101 | 48.7 (0.5) | NA | NA | NA | NA |
| 7 | 94 | 48.8 (0.5) | -0.3 (0.6) | 95 | 49.3 (0.5) | 0.6 (0.6) | -0.9 (0.9) | 0.11 | 0.3 |
| 30 | 88 | 48.2 (0.5) | -1 (0.6) | 91 | 48.5 (0.5) | -0.1 (0.6) | -0.8 (0.9) | 0.09 | 0.36 |

Abbreviations: NA, not applicable

[a]Intervention minus usual care group; change between groups was calculated as least square mean difference for follow-up (days 7 and 30) minus baseline; P values are from the time by group interaction term in mixed models; effect size for continuous variables was calculated as Westfall's d for the time by group interaction, with 0.2 being a small effect size, 0.5 medium effect size, and 0.8 large effect size.

[b]Adjusted for baseline PROMIS global mental t-score

© 2025 Bakitas MA et al. *JAMA Network Open*.

| Outcome<br>No. of days after baseline | Rating | Intervention | | | Usual Care | | | Between-group difference in change from baseline[a] | | |
|---|---|---|---|---|---|---|---|---|---|---|
| | | Participants, No. | %, mean (SE) | Change from baseline, mean (SE) | Participants, No. | %, mean (SE) | Change from baseline, mean (SE) | Mean (SE) | Effect size | P value |
| **FAMCARE** | | | | | | | | | | |
| Patients comfort | | | | | | | | | | |
| 0 | Very satisfied/Satisfied | 101 | 76.2 (4.2) | NA | 98 | 78.6 (4.1) | NA | NA | NA | NA |
| | Undecided/Dissatisfied/Very dissatisfied | | 23.8 (4.2) | NA | | 21.4 (4.1) | NA | NA | | NA |
| 7 | Very satisfied/Satisfied | 91 | 85.7 (3.7) | 9.5 (5.6) | 92 | 84.8 (3.7) | 6.2 (5.6) | 3.3 (7.9) | 0.03 | 0.68 |
| | Undecided/Dissatisfied/Very dissatisfied | | 14.3 (3.7) | -9.5 (5.6) | | 15.2 (3.7) | -6.2 (5.6) | -3.3 (7.9) | | 0.68 |
| 30 | Very satisfied/Satisfied | 87 | 94.3 (2.5) | 18 (4.9) | 90 | 90 (3.2) | 11.4 (5.2) | 6.6 (7.2) | 0.09 | 0.36 |
| | Undecided/Dissatisfied/Very dissatisfied | | 5.7 (2.5) | -18 (4.9) | | 10 (3.2) | -11.4 (5.2) | -6.6 (7.2) | | 0.36 |
| Condition and progress explained | | | | | | | | | | |
| 0 | Very satisfied/Satisfied | 98 | 70.8 (5.7) | NA | 91 | 71.9 (5.8) | NA | NA | NA | NA |
| | Undecided/Dissatisfied/Very dissatisfied | | 29.2 (5.7) | NA | | 28.1 (5.8) | NA | NA | | NA |
| 7 | Very satisfied/Satisfied | 88 | 80.6 (5) | 9.8 (6.7) | 89 | 85.4 (4.2) | 13.5 (6.4) | -3.7 (9.3) | -0.05 | 0.69 |
| | Undecided/Dissatisfied/Very dissatisfied | | 19.4 (5) | -9.8 (6.7) | | 14.6 (4.2) | -13.5 (6.4) | 3.7 (9.3) | | 0.69 |
| 30 | Very satisfied/Satisfied | 85 | 90.6 (3.3) | 19.8 (6.1) | 89 | 92.7 (2.8) | 20.8 (6) | -0.9 (8.5) | -0.03 | 0.91 |
| | Undecided/Dissatisfied/Very dissatisfied | | 9.4 (3.3) | -19.8 (6.1) | | 7.3 (2.8) | -20.8 (6) | 0.9 (8.5) | | 0.91 |
| Information on side effects | | | | | | | | | | |
| 0 | Very satisfied/Satisfied | 99 | 77 (5) | NA | 93 | 72.8 (5.7) | NA | NA | NA | NA |
| | Undecided/Dissatisfied/Very dissatisfied | | 23 (5) | NA | | 27.2 (5.7) | NA | NA | | NA |

© 2025 Bakitas MA et al. *JAMA Network Open*.

| Outcome<br>No. of days after baseline | Rating | Intervention | | | Usual Care | | | Between-group difference in change from baseline[a] | | |
|---|---|---|---|---|---|---|---|---|---|---|
| | | Participants, No. | %, mean (SE) | Change from baseline, mean (SE) | Participants, No. | %, mean (SE) | Change from baseline, mean (SE) | Mean (SE) | Effect size | P value |
| 7 | Very satisfied/Satisfied | 86 | 87.2 (3.9) | 10.2 (5.8) | 88 | 78.5 (5.2) | 5.7 (6.8) | 4.5 (8.9) | 0.06 | 0.61 |
| | Undecided/Dissatisfied/Very dissatisfied | | 12.8 (3.9) | -10.2 (5.8) | | 21.5 (5.2) | -5.7 (6.8) | -4.5 (8.9) | | 0.61 |
| 30 | Very satisfied/Satisfied | 85 | 91.3 (3.2) | 14.3 (5.5) | 88 | 86.4 (4.1) | 13.6 (6.3) | 0.7 (8.4) | 0.04 | 0.93 |
| | Undecided/Dissatisfied/Very dissatisfied | | 8.7 (3.2) | -14.3 (5.5) | | 13.6 (4.1) | -13.6 (6.3) | -0.7 (8.4) | | 0.93 |
| Respect for dignity | | | | | | | | | | |
| 0 | Very satisfied/Satisfied | 101 | 88.1 (3.2) | NA | 95 | 87.4 (3.4) | NA | NA | NA | NA |
| | Undecided/Dissatisfied/Very dissatisfied | | 11.9 (3.2) | NA | | 12.6 (3.4) | NA | NA | | NA |
| 7 | Very satisfied/Satisfied | 89 | 84.3 (3.9) | -3.8 (5) | 89 | 88.8 (3.3) | 1.4 (4.8) | -5.2 (6.9) | -0.06 | 0.45 |
| | Undecided/Dissatisfied/Very dissatisfied | | 15.7 (3.9) | 3.8 (5) | | 11.2 (3.3) | -1.4 (4.8) | 5.2 (6.9) | | 0.45 |
| 30 | Very satisfied/Satisfied | 86 | 91.9 (2.9) | 3.7 (4.4) | 89 | 93.3 (2.7) | 5.9 (4.3) | -2.1 (6.1) | -0.03 | 0.73 |
| | Undecided/Dissatisfied/Very dissatisfied | | 8.1 (2.9) | -3.7 (4.4) | | 6.7 (2.7) | -5.9 (4.3) | 2.1 (6.1) | | 0.73 |
| Meetings with care team | | | | | | | | | | |
| 0 | Very satisfied/Satisfied | 89 | 64.4 (6.2) | NA | 87 | 68.2 (6.1) | NA | NA | NA | NA |
| | Undecided/Dissatisfied/Very dissatisfied | | 35.6 (6.2) | NA | | 31.8 (6.1) | NA | NA | | NA |
| 7 | Very satisfied/Satisfied | 83 | 85.7 (4.3) | 21.4 (6.9) | 84 | 83 (4.6) | 14.8 (6.9) | 6.5 (9.8) | 0.06 | 0.5 |
| | Undecided/Dissatisfied/Very dissatisfied | | 14.3 (4.3) | -21.4 (6.9) | | 17 (4.6) | -14.8 (6.9) | -6.5 (9.8) | | 0.5 |
| 30 | Very satisfied/Satisfied | 81 | 87.2 (4) | 22.9 (6.8) | 85 | 89.7 (3.5) | 21.5 (6.5) | 1.4 (9.4) | -0.01 | 0.89 |
| | Undecided/Dissatisfied/Very dissatisfied | | 12.8 (4) | -22.9 (6.8) | | 10.3 (3.5) | -21.5 (6.5) | -1.4 (9.4) | | 0.89 |

© 2025 Bakitas MA et al. *JAMA Network Open*.

| Outcome<br>No. of days after baseline | Rating | Intervention | | | Usual Care | | | Between-group difference in change from baseline[a] | | |
|---|---|---|---|---|---|---|---|---|---|---|
| | | Participants, No. | %, mean (SE) | Change from baseline, mean (SE) | Participants, No. | %, mean (SE) | Change from baseline, mean (SE) | Mean (SE) | Effect size | P value |
| Speed of treated | | | | | | | | | | |
| 0 | Very satisfied/Satisfied | 102 | 71 (5.7) | NA | 98 | 62.4 (6.3) | NA | NA | NA | NA |
| | Undecided/Dissatisfied/Very dissatisfied | | 29 (5.7) | NA | | 37.6 (6.3) | NA | NA | | NA |
| 7 | Very satisfied/Satisfied | 90 | 78.8 (5.3) | 7.8 (6.8) | 89 | 81.7 (4.8) | 19.3 (7) | -11.5 (9.7) | -0.09 | 0.24 |
| | Undecided/Dissatisfied/Very dissatisfied | | 21.2 (5.3) | -7.8 (6.8) | | 18.3 (4.8) | -19.3 (7) | 11.5 (9.7) | | 0.24 |
| 30 | Very satisfied/Satisfied | 87 | 91.6 (3.1) | 20.6 (6) | 89 | 87.1 (3.9) | 24.6 (6.7) | -4.1 (9) | 0.01 | 0.65 |
| | Undecided/Dissatisfied/Very dissatisfied | | 8.4 (3.1) | -20.6 (6) | | 12.9 (3.9) | -24.6 (6.7) | 4.1 (9) | | 0.65 |
| Attention to symptom description | | | | | | | | | | |
| 0 | Very satisfied/Satisfied | 102 | 80.6 (4.8) | NA | 96 | 74.8 (5.6) | NA | NA | NA | NA |
| | Undecided/Dissatisfied/Very dissatisfied | | 19.4 (4.8) | NA | | 25.2 (5.6) | NA | NA | | NA |
| 7 | Very satisfied/Satisfied | 91 | 82.4 (4.8) | 1.8 (6) | 89 | 84.1 (4.5) | 9.3 (6.3) | -7.6 (8.7) | -0.07 | 0.38 |
| | Undecided/Dissatisfied/Very dissatisfied | | 17.6 (4.8) | -1.8 (6) | | 15.9 (4.5) | -9.3 (6.3) | 7.6 (8.7) | | 0.38 |
| 30 | Very satisfied/Satisfied | 86 | 92.8 (2.8) | 12.2 (5.1) | 88 | 94.1 (2.5) | 19.2 (5.7) | -7 (7.7) | -0.07 | 0.36 |
| | Undecided/Dissatisfied/Very dissatisfied | | 7.2 (2.8) | -12.2 (5.1) | | 5.9 (2.5) | -19.2 (5.7) | 7 (7.7) | | 0.36 |
| Physical needs met | | | | | | | | | | |
| 0 | Very satisfied/Satisfied | 101 | 84.8 (4.1) | NA | 97 | 83.1 (4.4) | NA | NA | NA | NA |
| | Undecided/Dissatisfied/Very dissatisfied | | 15.2 (4.1) | NA | | 16.9 (4.4) | NA | NA | | NA |
| 7 | Very satisfied/Satisfied | 89 | 85.1 (4.2) | 0.3 (5.3) | 91 | 85.3 (4.2) | 2.2 (5.4) | -1.9 (7.6) | -0.02 | 0.8 |

© 2025 Bakitas MA et al. *JAMA Network Open*.

| Outcome No. of days after baseline | Rating | Intervention | | | Usual Care | | | Between-group difference in change from baseline[a] | | |
|---|---|---|---|---|---|---|---|---|---|---|
| | | Participants, No. | %, mean (SE) | Change from baseline, mean (SE) | Participants, No. | %, mean (SE) | Change from baseline, mean (SE) | Mean (SE) | Effect size | P value |
| | Undecided/Dissatisfied/Very dissatisfied | | 14.9 (4.2) | -0.3 (5.3) | | 14.7 (4.2) | -2.2 (5.4) | 1.9 (7.6) | | 0.8 |
| 30 | Very satisfied/Satisfied | 87 | 92.8 (2.8) | 8.1 (4.6) | 89 | 93.4 (2.6) | 10.3 (4.7) | -2.2 (6.6) | -0.03 | 0.73 |
| | Undecided/Dissatisfied/Very dissatisfied | | 7.2 (2.8) | -8.1 (4.6) | | 6.6 (2.6) | -10.3 (4.7) | 2.2 (6.6) | | 0.73 |
| Availability of care team | | | | | | | | | | |
| 0 | Very satisfied/Satisfied | 100 | 75.5 (5.2) | NA | 95 | 73.9 (5.5) | NA | NA | NA | NA |
| | Undecided/Dissatisfied/Very dissatisfied | | 24.5 (5.2) | NA | | 26.1 (5.5) | NA | NA | | NA |
| 7 | Very satisfied/Satisfied | 87 | 85.4 (4.3) | 9.9 (6) | 89 | 87.7 (3.8) | 13.9 (6.1) | -4 (8.5) | -0.04 | 0.64 |
| | Undecided/Dissatisfied/Very dissatisfied | | 14.6 (4.3) | -9.9 (6) | | 12.3 (3.8) | -13.9 (6.1) | 4 (8.5) | | 0.64 |
| 30 | Very satisfied/Satisfied | 84 | 90.8 (3.3) | 15.3 (5.6) | 88 | 94.5 (2.4) | 20.6 (5.7) | -5.3 (8) | -0.08 | 0.5 |
| | Undecided/Dissatisfied/Very dissatisfied | | 9.2 (3.3) | -15.3 (5.6) | | 5.5 (2.4) | -20.6 (5.7) | 5.3 (8) | | 0.5 |
| Emotional support to family | | | | | | | | | | |
| 0 | Very satisfied/Satisfied | 89 | 76 (5.3) | NA | 87 | 79.4 (4.9) | NA | NA | NA | NA |
| | Undecided/Dissatisfied/Very dissatisfied | | 24 (5.3) | NA | | 20.6 (4.9) | NA | NA | | NA |
| 7 | Very satisfied/Satisfied | 85 | 81.2 (4.8) | 5.2 (6.5) | 86 | 85 (4.2) | 5.6 (5.9) | -0.4 (8.8) | -0.01 | 0.97 |
| | Undecided/Dissatisfied/Very dissatisfied | | 18.8 (4.8) | -5.2 (6.5) | | 15 (4.2) | -5.6 (5.9) | 0.4 (8.8) | | 0.97 |
| 30 | Very satisfied/Satisfied | 85 | 90.9 (3.3) | 14.9 (5.8) | 86 | 91.3 (3.1) | 11.9 (5.4) | 3 (7.9) | 0.02 | 0.71 |
| | Undecided/Dissatisfied/Very dissatisfied | | 9.1 (3.3) | -14.9 (5.8) | | 8.7 (3.1) | -11.9 (5.4) | -3 (7.9) | | 0.71 |

© 2025 Bakitas MA et al. *JAMA Network Open*.

| Outcome No. of days after baseline | Rating | Intervention | | | Usual Care | | | Between-group difference in change from baseline[a] | | |
|---|---|---|---|---|---|---|---|---|---|---|
| | | Participants, No. | %, mean (SE) | Change from baseline, mean (SE) | Participants, No. | %, mean (SE) | Change from baseline, mean (SE) | Mean (SE) | Effect size | P value |
| Practical assistance | | | | | | | | | | |
| 0 | Very satisfied/Satisfied | 65 | 86.2 (4.3) | NA | 76 | 78.9 (4.7) | NA | NA | NA | NA |
| | Undecided/Dissatisfied/Very dissatisfied | | 13.8 (4.3) | NA | | 21.1 (4.7) | NA | NA | | NA |
| 7 | Very satisfied/Satisfied | 65 | 80 (5) | -6.2 (6.6) | 67 | 85.1 (4.4) | 6.1 (6.4) | -12.3 (9.2) | -0.13 | 0.18 |
| | Undecided/Dissatisfied/Very dissatisfied | | 20 (5) | 6.2 (6.6) | | 14.9 (4.4) | -6.1 (6.4) | 12.3 (9.2) | | 0.18 |
| 30 | Very satisfied/Satisfied | 58 | 84.5 (4.8) | -1.7 (6.4) | 68 | 85.3 (4.3) | 6.3 (6.3) | -8 (9) | -0.09 | 0.37 |
| | Undecided/Dissatisfied/Very dissatisfied | | 15.5 (4.8) | 1.7 (6.4) | | 14.7 (4.3) | -6.3 (6.3) | 8 (9) | | 0.37 |
| Doctors attention to symptoms | | | | | | | | | | |
| 0 | Very satisfied/Satisfied | 101 | 80.2 (4) | NA | 93 | 80.6 (4.1) | NA | NA | NA | NA |
| | Undecided/Dissatisfied/Very dissatisfied | | 19.8 (4) | NA | | 19.4 (4.1) | NA | NA | | NA |
| 7 | Very satisfied/Satisfied | 89 | 84.3 (3.9) | 4.1 (5.5) | 87 | 85.1 (3.8) | 4.4 (5.6) | -0.3 (7.9) | -0.01 | 0.97 |
| | Undecided/Dissatisfied/Very dissatisfied | | 15.7 (3.9) | -4.1 (5.5) | | 14.9 (3.8) | -4.4 (5.6) | 0.3 (7.9) | | 0.97 |
| 30 | Very satisfied/Satisfied | 84 | 92.9 (2.8) | 12.7 (4.9) | 87 | 88.5 (3.4) | 7.9 (5.3) | 4.8 (7.2) | 0.07 | 0.51 |
| | Undecided/Dissatisfied/Very dissatisfied | | 7.1 (2.8) | -12.7 (4.9) | | 11.5 (3.4) | -7.9 (5.3) | -4.8 (7.2) | | 0.51 |
| Family is included | | | | | | | | | | |
| 0 | Very satisfied/Satisfied | 101 | 81.2 (3.9) | NA | 90 | 75.6 (4.5) | NA | NA | NA | NA |
| | Undecided/Dissatisfied/Very dissatisfied | | 18.8 (3.9) | NA | | 24.4 (4.5) | NA | NA | | NA |
| 7 | Very satisfied/Satisfied | 89 | 85.4 (3.7) | 4.2 (5.4) | 89 | 86.5 (3.6) | 11 (5.8) | -6.8 (7.9) | -0.07 | 0.39 |

© 2025 Bakitas MA et al. *JAMA Network Open*.

| Outcome No. of days after baseline | Rating | Intervention | | | Usual Care | | | Between-group difference in change from baseline[a] | | |
|---|---|---|---|---|---|---|---|---|---|---|
| | | Participants, No. | %, mean (SE) | Change from baseline, mean (SE) | Participants, No. | %, mean (SE) | Change from baseline, mean (SE) | Mean (SE) | Effect size | P value |
| | Undecided/Dissatisfied/Very dissatisfied | | 14.6 (3.7) | -4.2 (5.4) | | 13.5 (3.6) | -11 (5.8) | 6.8 (7.9) | | 0.39 |
| 30 | Very satisfied/Satisfied | 83 | 89.2 (3.4) | 8 (5.2) | 88 | 92 (2.9) | 16.5 (5.4) | -8.5 (7.5) | -0.09 | 0.25 |
| | Undecided/Dissatisfied/Very dissatisfied | | 10.8 (3.4) | -8 (5.2) | | 8 (2.9) | -16.5 (5.4) | 8.5 (7.5) | | 0.25 |
| Information about managing symptoms | | | | | | | | | | |
| 0 | Very satisfied/Satisfied | 79 | 68.6 (6.1) | NA | 74 | 69.6 (6.3) | NA | NA | NA | NA |
| | Undecided/Dissatisfied/Very dissatisfied | | 31.4 (6.1) | NA | | 30.4 (6.3) | NA | NA | | NA |
| 7 | Very satisfied/Satisfied | 68 | 83.8 (4.9) | 15.2 (7.3) | 70 | 81.2 (5.2) | 11.6 (7.5) | 3.6 (10.5) | 0.04 | 0.73 |
| | Undecided/Dissatisfied/Very dissatisfied | | 16.2 (4.9) | -15.2 (7.3) | | 18.8 (5.2) | -11.6 (7.5) | -3.6 (10.5) | | 0.73 |
| 30 | Very satisfied/Satisfied | 63 | 84.9 (4.9) | 16.2 (7.3) | 80 | 92 (3) | 22.4 (6.6) | -6.2 (9.9) | -0.1 | 0.53 |
| | Undecided/Dissatisfied/Very dissatisfied | | 15.1 (4.9) | -16.2 (7.3) | | 8 (3) | -22.4 (6.6) | 6.2 (9.9) | | 0.53 |
| Effectiveness of care team | | | | | | | | | | |
| 0 | Very satisfied/Satisfied | 100 | 81.4 (4.7) | NA | 95 | 78.9 (5.1) | NA | NA | NA | NA |
| | Undecided/Dissatisfied/Very dissatisfied | | 18.6 (4.7) | NA | | 21.1 (5.1) | NA | NA | | NA |
| 7 | Very satisfied/Satisfied | 90 | 83.6 (4.6) | 2.1 (5.8) | 89 | 90.7 (3.3) | 11.7 (5.4) | -9.6 (7.9) | -0.12 | 0.23 |
| | Undecided/Dissatisfied/Very dissatisfied | | 16.4 (4.6) | -2.1 (5.8) | | 9.3 (3.3) | -11.7 (5.4) | 9.6 (7.9) | | 0.23 |
| 30 | Very satisfied/Satisfied | 85 | 91.7 (3.1) | 10.3 (5.1) | 89 | 92.4 (2.9) | 13.4 (5.4) | -3.2 (7.4) | -0.03 | 0.67 |
| | Undecided/Dissatisfied/Very dissatisfied | | 8.3 (3.1) | -10.3 (5.1) | | 7.6 (2.9) | -13.4 (5.4) | 3.2 (7.4) | | 0.67 |

© 2025 Bakitas MA et al. *JAMA Network Open*.

| Outcome No. of days after baseline | Rating | Intervention | | | Usual Care | | | Between-group difference in change from baseline[a] | | |
|---|---|---|---|---|---|---|---|---|---|---|
| | | Participants, No. | %, mean (SE) | Change from baseline, mean (SE) | Participants, No. | %, mean (SE) | Change from baseline, mean (SE) | Mean (SE) | Effect size | P value |
| Response to changes | | | | | | | | | | |
| 0 | Very satisfied/Satisfied | 100 | 77 (4.2) | NA | 94 | 77.7 (4.3) | NA | NA | NA | NA |
| | Undecided/Dissatisfied/Very dissatisfied | | 23 (4.2) | NA | | 22.3 (4.3) | NA | NA | | NA |
| 7 | Very satisfied/Satisfied | 87 | 83.9 (3.9) | 6.9 (5.8) | 86 | 89.5 (3.3) | 11.9 (5.4) | -5 (7.9) | -0.07 | 0.53 |
| | Undecided/Dissatisfied/Very dissatisfied | | 16.1 (3.9) | -6.9 (5.8) | | 10.5 (3.3) | -11.9 (5.4) | 5 (7.9) | | 0.53 |
| 30 | Very satisfied/Satisfied | 83 | 92.8 (2.8) | 15.8 (5.1) | 88 | 92 (2.9) | 14.4 (5.2) | 1.4 (7.3) | 0.02 | 0.85 |
| | Undecided/Dissatisfied/Very dissatisfied | | 7.2 (2.8) | -15.8 (5.1) | | 8 (2.9) | -14.4 (5.2) | -1.4 (7.3) | | 0.85 |
| Emotional support to patient | | | | | | | | | | |
| 0 | Very satisfied/Satisfied | 99 | 79.8 (4) | NA | 90 | 80 (4.2) | NA | NA | NA | NA |
| | Undecided/Dissatisfied/Very dissatisfied | | 20.2 (4) | NA | | 20 (4.2) | NA | NA | | NA |
| 7 | Very satisfied/Satisfied | 87 | 89.7 (3.3) | 9.9 (5.2) | 88 | 87.5 (3.5) | 7.5 (5.5) | 2.4 (7.6) | 0.03 | 0.76 |
| | Undecided/Dissatisfied/Very dissatisfied | | 10.3 (3.3) | -9.9 (5.2) | | 12.5 (3.5) | -7.5 (5.5) | -2.4 (7.6) | | 0.76 |
| 30 | Very satisfied/Satisfied | 83 | 89.2 (3.4) | 9.4 (5.3) | 88 | 90.9 (3.1) | 10.9 (5.2) | -1.6 (7.4) | -0.03 | 0.83 |

Abbreviations: NA, not applicable

[a]Intervention minus usual care group; change between groups was calculated as least square mean difference for follow-up (days 7 and 30) minus baseline; P values are from the time by group interaction term in mixed models; effect size for continuous variables was calculated as Westfall's d for the time by group interaction, with 0.2 being a small effect size, 0.5 medium effect size, and 0.8 large effect size.

© 2025 Bakitas MA et al. *JAMA Network Open*.

**eTable 9. Caregiver Heard and Understood From Baseline to 30 Days (Intervention vs Usual Care)**

| Time after baseline, days | Rating | Intervention | | | Usual Care | | | Between-group difference in change from baseline[a] | |
|---|---|---|---|---|---|---|---|---|---|
| | | Participants, No. | %, mean (SE) | Change from baseline, mean (SE) | Participants, No. | %, mean (SE) | Change from baseline, mean (SE) | Mean (SE) | P value |
| 0 | Completely/Quite a bit | 94 | 77.7 (4.3) | NA | 86 | 65.1 (5.1) | NA | NA | NA |
| | Moderately/Slightly/Not at all | | 22.3 (4.3) | NA | | 34.9 (5.1) | NA | NA | NA |
| 7 | Completely/Quite a bit | 90 | 88.9 (3.3) | 11.2 (5.4) | 89 | 88.8 (3.3) | 23.6 (6.1) | -12.4 (8.2) | 0.13 |
| | Moderately/Slightly/Not at all | | 11.1 (3.3) | -11.2 (5.4) | | 11.2 (3.3) | -23.6 (6.1) | 12.4 (8.2) | 0.13 |
| 30 | Completely/Quite a bit | 86 | 88.4 (3.5) | 10.7 (5.5) | 89 | 91 (3) | 25.9 (6) | -15.2 (8.1) | 0.06 |
| | Moderately/Slightly/Not at all | | 11.6 (3.5) | -10.7 (5.5) | | 9 (3) | -25.9 (6) | 15.2 (8.1) | 0.06 |

Abbreviation: NA, not applicable.

[a]Intervention minus usual care group; change between groups was calculated as least square mean difference for follow-up (days 7 and 30) minus baseline; P values are from the time by group interaction term in mixed models; effect size was calculated as Westfall's d for the time by group interaction.

© 2025 Bakitas MA et al. *JAMA Network Open*.

eFigure 1 Patient Satisfaction with Technology

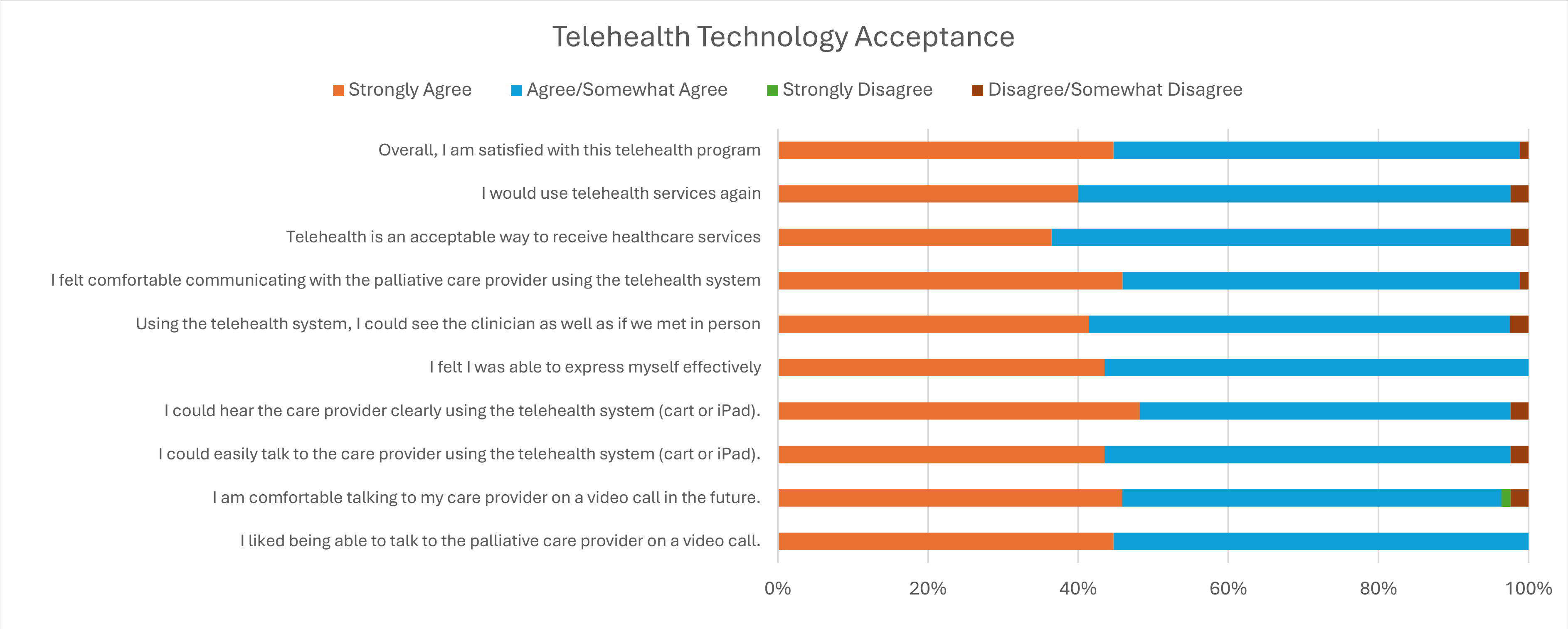


© 2025 Bakitas MA et al. *JAMA Network Open*.

**eFigure 2 Patients Feeling Heard and Understood**

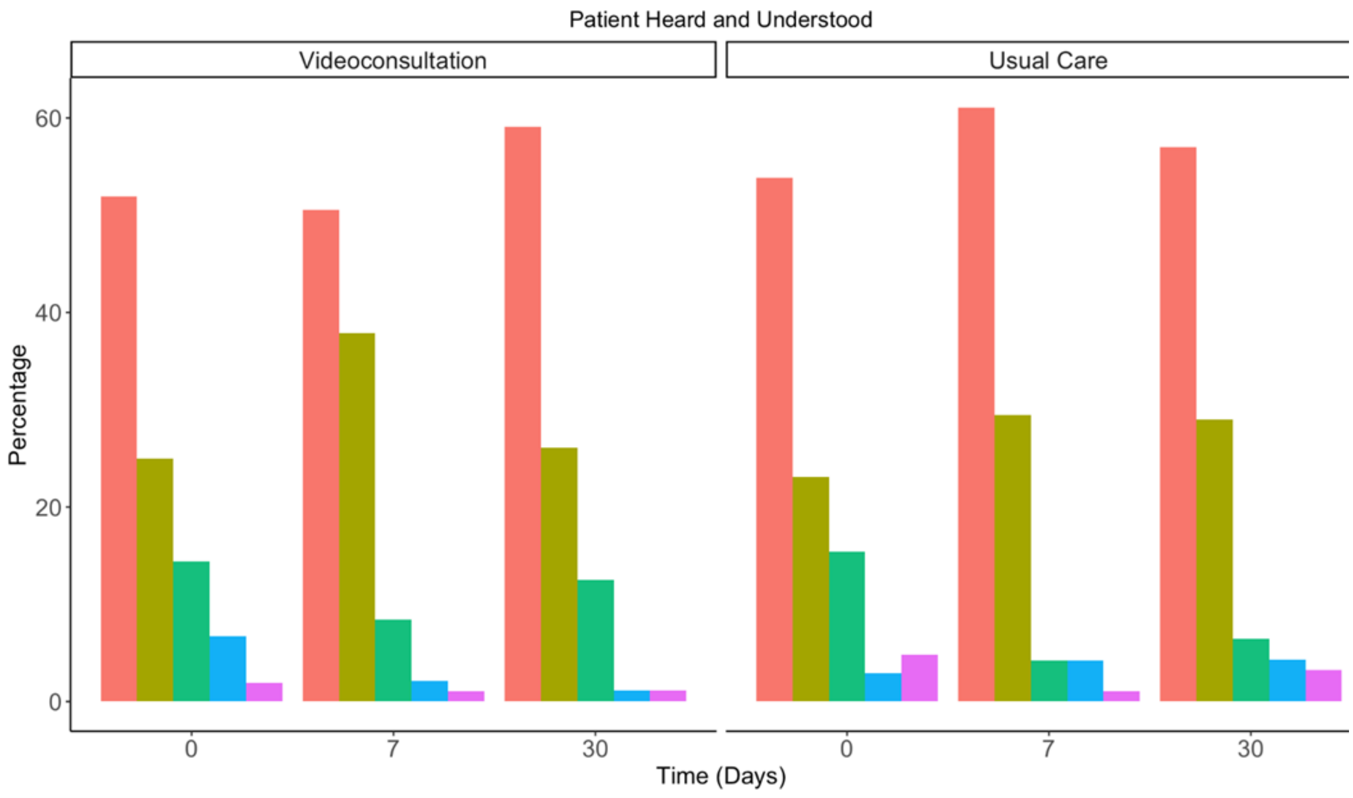


© 2025 Bakitas MA et al. *JAMA Network Open*.
