# Supplementary material for: Palliative Video Consultation and Symptom Distress Among Rural Inpatients: A Randomized Clinical Trial
Source: JAMA Netw Open. 2025 Jul 9;8(7):e2519426. doi: 10.1001/jamanetworkopen.2025.19426 (PMC12242707; doi:10.1001/jamanetworkopen.2025.19426)
Supplement: Supplement 3. — Data Sharing Statement [file jamanetwopen-e2519426-s003.pdf]

Data Sharing Statement

Bakitas. Palliative Video Consultation and Symptom Distress Among Rural Inpatients. *JAMA Netw Open*. Published July 09, 2025. doi:10.1001/jamanetworkopen.2025.19426

Data
Additional Information: Trial Registration: ClinicalTrials.gov Identifier: NCT03767517
Data available: Yes
Data types: Deidentified participant data, Data dictionary
How to access data: Data will be made available with an approved protocol from the corresponding author
When available: With publication

Supporting Documents
Document types: Informed consent form
How to access documents: mbakitas@uab.edu
When available: With publication

Additional Information
Who can access the data: Researchers requesting data with an approved protocol
Types of analyses: for meta-analyses, or other approved protocol analysis
Mechanisms of data availability: after approval of a proposal with a signed data access agreement
Any additional restrictions: NA
